# Test-retest reliability of a computer-assisted self-administered questionnaire on early life exposure in a nasopharyngeal carcinoma case-control study

**DOI:** 10.1038/s41598-018-25046-y

**Published:** 2018-05-04

**Authors:** Zhi-Ming Mai, Jia-Huang Lin, Shing-Chun Chiang, Roger Kai-Cheong Ngan, Dora Lai-Wan Kwong, Wai-Tong Ng, Alice Wan-Ying Ng, Kam-Tong Yuen, Kai-Ming Ip, Yap-Hang Chan, Anne Wing-Mui Lee, Sai-Yin Ho, Maria Li Lung, Tai-Hing Lam

**Affiliations:** 1 School of Public Health, The University of Hong Kong, Hong Kong S.A.R., China; 2 Centre for Nasopharyngeal Carcinoma Research (CNPCR), Research Grants Council Area of Excellence Scheme, The University of Hong Kong, Hong Kong S.A.R., China; 3 Department of Medicine, Queen Mary Hospital, The University of Hong Kong, Hong Kong S.A.R., China; 4 Department of Clinical Oncology, Queen Mary Hospital, The University of Hong Kong, Hong Kong S.A.R., China; 5 Department of Clinical Oncology, Queen Elizabeth Hospital, Hong Kong S.A.R., China; 6 Department of Clinical Oncology, Pamela Youde Nethersole Eastern Hospital, Hong Kong S.A.R., China; 7 Department of Clinical Oncology, Tuen Mun Hospital, Hong Kong S.A.R., China; 8 Department of Clinical Oncology, Princess Margaret Hospital, Hong Kong S.A.R., China

## Abstract

We evaluated the reliability of early life nasopharyngeal carcinoma (NPC) aetiology factors in the questionnaire of an NPC case-control study in Hong Kong during 2014–2017. 140 subjects aged 18+ completed the same computer-assisted questionnaire twice, separated by at least 2 weeks. The questionnaire included most known NPC aetiology factors and the present analysis focused on early life exposure. Test-retest reliability of all the 285 questionnaire items was assessed in all subjects and in 5 subgroups defined by cases/controls, sex, time between 1^st^ and 2^nd^ questionnaire (2–29/≥30 weeks), education (secondary or less/postsecondary), and age (25–44/45–59/60+ years) at the first questionnaire. The reliability of items on dietary habits, body figure, skin tone and sun exposure in early life periods (age 6–12 and 13–18) was moderate-to-almost perfect, and most other items had fair-to-substantial reliability in all life periods (age 6–12, 13–18 and 19–30, and 10 years ago). Differences in reliability by strata of the 5 subgroups were only observed in a few items. This study is the first to report the reliability of an NPC questionnaire, and make the questionnaire available online. Overall, our questionnaire had acceptable reliability, suggesting that previous NPC study results on the same risk factors would have similar reliability.

## Introduction

The aetiology of nasopharyngeal carcinoma (NPC) remains largely unclear. Multiple factors are implicated, including Epstein-Barr virus (EBV) infection, genetic susceptibility and environmental factors^1^. EBV infection is ubiquitous worldwide while NPC remains a rare cancer in most parts of the world^2^. Genetic predisposition is clearly associated with NPC, but the decline in risks for NPC observed in subsequent generations of Chinese migrants^3–6^ strongly suggests that the role of environmental exposure is crucial. The most commonly used method to measure environmental exposure is through self-reports in questionnaires and interviews.

NPC incidence in Southern China increases with age, and decreases after the peak at age 40–59^7^, highlighting the importance of early life exposure. Indeed, previous case-control studies observed that salted fish consumption during childhood seems more strongly related to NPC risk than adulthood exposure^8–14^. To understand the role of early life exposure, the ideal would be a population-based birth cohort study with repeated measures of the exposure, and follow-up of the subjects in a few decades for NPC outcomes. Given that cohort studies are not feasible, retrospective case-control studies with high-quality data on early life exposure remain essential for research on NPC and other rare cancers. Nevertheless, information on early childhood exposure collected by interview and questionnaire relying on adult recall is subject to recall errors (random and systematic). Studies of reliability can inform researchers about such errors. The reliability of a survey instrument is most commonly assessed using the test-retest method, comparing responses given in the second survey with those in the first^15^. However, the reliability of questionnaires used in case-control studies were rarely reported. No evidence of test-retest reliability of NPC questionnaires was found. Such evidence is especially important on early life exposure, which would have greater error than adulthood exposure.

We evaluated the reliability of early life NPC aetiology factors in the questionnaire of an NPC case-control study in Hong Kong.

## Results

Seventy subjects in Queen Mary Hospital (QMH, 69% of NPC cases and 80% of non-NPC hospital controls of all eligible subjects), and 70 in Queen Elizabeth Hospital (QEH, 51% and 58%) were included (Supplementary Fig. s1), with an average of 31.4 (standard deviation: 18.7) weeks and 29.5 (standard deviation: 19.0) weeks between the two interviews, respectively.

**Figure 1:**
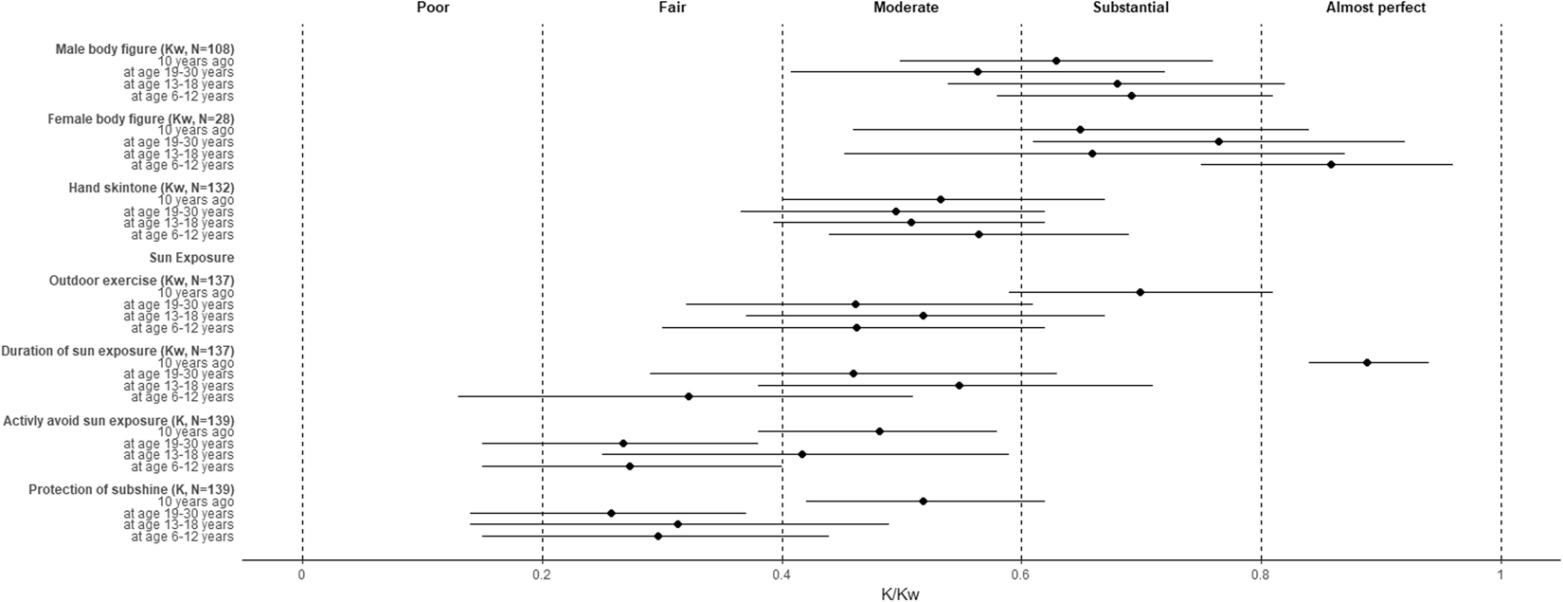
Coefficients (95% confidence interval) for body figure, skin tone and sun exposure at different life periods.

Figure 1 shows that for body figures and hand skin tone, similar weighted Kappa coefficients across life periods were observed. In all life periods (age 6–12, 13–18 and 19–30, and 10 years ago), moderate-to-almost perfect reliability coefficients for body figures were found (0.70, 0.69, 0.56 and 0.63 for males, and 0.86, 0.66, 0.76 and 0.65 for females, respectively). For hand skin tone, moderate reliability coefficients were observed in all life periods (0.56, 0.51, 0.50 and 0.53, respectively).

However, for sun exposure, reliability coefficients differed by life periods (Fig. 1). The reliability coefficient for duration of sun exposure 10 years ago was 0.89, which was higher than those in earlier periods (age 19–30: 0.46, age 13–19: 0.55 and age 6–12: 0.32) (Fisher Z transformation p < 0.01 with Bonferonni correction). For “actively avoid sun exposure” and “protection from sunshine” (never/ever), moderate reliability coefficients were found for those 10 years ago (0.48 and 0.52). However, the coefficients were lower for age 19–30 (0.27 and 0.42), age 13–19 (0.27 and 0.26) and age 6–12 (0.31 and 0.30).

Table 1 shows moderate-to-substantial reliability coefficients (0.4–0.8) for most food frequency questionnaire (FFQ) items at age 6–12 and 13–18, and the coefficients were generally higher for frequency than portion size items. The reliability coefficients of FFQ at age 6–12 and 13–18 were similar (Table 2). For salted fish consumption, most reliability coefficients were substantial-to-almost perfect (0.6–1.0) in both periods.

**Table 1:** Reliability coefficients (95% confidence intervals: 95% CI) for food frequency questionnaire in early-life periods, including late childhood (6–12 years) and adolescence (13–18 years).

FFQ at age 6–12 years		Coefficients (N, methods^‡^), 95% CIs
All meat	Frequency	**0.67** (137), 0.52–0.77
	Portion	**0.46** (131), 0.23–0.61
Animal liver	Frequency	**0.69** (138), 0.57–0.78
	Portion	**0.20** (99), -0.19, 0.46^#^
All fish	Frequency	**0.67** (138), 0.54–0.77
	Portion	**0.46** (136), 0.24–0.61
All fruits	Frequency	**0.75** (138), 0.66–0.82
	Portion	**0.56** (119), 0.36–0.69
All vegetables	Frequency	**0.75** (139), 0.64–0.83
	Portion	**0.61** (136), 0.45–0.72
Fresh milk	Frequency	**0.70** (139), 0.57–0.79
	Portion	**0.65** (70), 0.44–0.79
Powdered milk	Frequency	**0.51** (138), 0.31–0.65
	Portion	**0.40** (30), -0.28, 0.72^#^
Soybean milk	Frequency	**0.73** (139), 0.63–0.81
	Portion	**0.51** (82), 0.24–0.69
Type of salted fish		**0.28** (140, K), 0.18–0.38
Mouldy salted fish	Frequency	**0.77** (33), 0.53–0.89
	Portion	**0.85** (33), 0.70–0.93
Firmed salted fish	Frequency	**0.84** (29), 0.65–0.92
	Portion	**0.71** (29), 0.37–0.86
Other types of salted fish	Frequency	**0.94** (9), 0.75–0.99
	Portion	**0.92** (8), 0.64–0.98
Preserved meat	Frequency	**0.65** (137), 0.52–0.75
	Portion	**0.67** (110), 0.52–0.78
Preserved egg	Frequency	**0.62** (139), 0.47–0.73
	Portion	**0.64** (111), 0.47–0.75
Preserved vegetable	Frequency	**0.67** (138), 0.54–0.77
	Portion	**0.54** (98), 0.31–0.69
Preserved fruit	Frequency	**0.61** (138), 0.50–0.71
	Portion	**0.61** (81), 0.39–0.75
**FFQ at age 13–18 years**		
Red meat	Frequency	**0.48** (139), 0.35–0.60
	Portion	**0.39** (138), 0.23–0.52
Poultry	Frequency	**0.55** (139), 0.42–0.66
	Portion	**0.44** (131), 0.29–0.57
Animal liver	Frequency	**0.57** (139), 0.44–0.67
	Portion	**0.46** (102), 0.29–0.60
Oily fish	Frequency	**0.60** (139), 0.48–0.70
	Portion	**0.53** (79), 0.36–0.67
Non-oily fish	Frequency	**0.56** (139), 0.43–0.66
	Portion	**0.26** (115), 0.08–0.42^†^
Shellfish	Frequency	**0.61** (139), 0.49–0.70
	Portion	**0.40** (114), 0.23–0.54
Leafy green vegetable	Frequency	**0.44** (138), 0.29–0.56
	Portion	**0.44** (137), 0.29–0.57
Other vegetables	Frequency	**0.38** (140), 0.22–0.51
	Portion	**0.60** (129), 0.47–0.70
Carrot	Frequency	**0.64** (140), 0.53–0.72
	Portion	**0.41** (108), 0.24–0.55
Tomato	Frequency	**0.53** (139), 0.40–0.64
	Portion	**0.42** (124), 0.26–0.55
Citrus fruit	Frequency	**0.59** (139), 0.47–0.69
	Portion	**0.48** (127), 0.33–0.60
Other fruits	Frequency	**0.55** (140), 0.42–0.65
	Portion	**0.38** (130), 0.22–0.52
Fresh milk	Frequency	**0.74** (139), 0.65–0.80
	Portion	**0.53** (79), 0.35–0.67
Powdered milk	Frequency	**0.44** (139), 0.30–0.57
	Portion	**0.35** (30), 0.01–0.63^†^
Dairy products	Frequency	**0.64** (140), 0.53–0.73
	Portion	**0.41** (106), 0.24–0.55
Egg	Frequency	**0.57** (140), 0.45–0.67
	Portion	**0.29** (132), 0.12–0.44
Tofu	Frequency	**0.59** (140), 0.47–0.69
	Portion	**0.45** (126), 0.30–0.58
Soybean milk	Frequency	**0.63** (137), 0.51–0.72
	Portion	**0.44** (96), 0.27–0.59
Bean curd	Frequency	**0.68** (139), 0.58–0.76
	Portion	**0.36** (112), 0.19–0.51
Types of salted fish		**0.31** (139, K), 0.21–0.41
Mouldy salted fish	Frequency	**0.85** (26), 0.68–0.93
	Portion	**0.69** (26), 0.43–0.85
Firmed salted fish	Frequency	**0.64** (36), 0.40–0.80
	Portion	**0.56** (34), 0.28–0.76
Other types of salted fish	Frequency	**0.94** (13), 0.81–0.98
	Portion	**0.38** (13), -0.22, 0.76^#^
Preserved seafood	Frequency	**0.57** (139), 0.45–0.67
	Portion	**0.45** (112), 0.29–0.59
Preserved vegetable	Frequency	**0.59** (139), 0.47–0.69
	Portion	**0.47** (114), 0.31–0.60
Preserved fruit	Frequency	**0.66** (140), 0.55–0.74
	Portion	**0.38** (90), 0.19–0.55
Preserved egg	Frequency	**0.62** (139), 0.51–0.72
	Portion	**0.48** (112), 0.33–0.61
Preserved meat	Frequency	**0.50** (139), 0.37–0.62
	Portion	**0.49** (113), 0.34–0.62
Processed meat	Frequency	**0.65** (140), 0.55–0.74
	Portion	**0.42** (100), 0.24–0.57
Condiments	Frequency	**0.40** (139), 0.25–0.53
	Portion	**0.28** (125), 0.11–0.43
Green/white tea	Frequency	**0.49** (140), 0.36–0.61
	Portion	**0.34** (77), 0.13–0.52
Oolong tea	Frequency	**0.64** (140), 0.53–0.73
	Portion	**0.08** (70), -0.15, 0.30^#^
Red/black tea	Frequency	**0.63** (140), 0.52–0.72
	Portion	**0.28** (88), 0.08–0.47^†^
Cantonese-style milk tea	Frequency	**0.70** (140), 0.60–0.77
	Portion	**0.23** (70), -0.01, 0.44^#^
Coffee	Frequency	**0.67** (139), 0.57–0.75
	Portion	**0.39** (44), 0.11–0.62
Chinese herbal tea	Frequency	**0.63** (139), 0.52–0.72
	Portion	**0.37** (109), 0.20–0.52

FFQ: food frequency questionnaire. Frequency: Never/Less than once a month/Once a month/1–3 per week/4–6 per week/1–2 per day/3+ per day). Portion: Small/Medium/Large. K: Cohen’s kappa. ^‡^Intra-class correlation coefficient was used in most items, unless otherwise stated. Coefficients: 0 to 0.2, poor; 0.2 to <0.4, fair; 0.4 to <0.6, moderate; 0.6 to <0.8, substantial; and 0.8 to 1.0, almost perfect. All the coefficients above were p < 0.01, unless otherwise stated (^†^0.01 ≤ p ≤ 0.05; ^#^p > 0.05 [*-x, x*]).

**Table 2:** Reliability coefficients (95% confidence intervals: 95% CI) for food items that were measured in both periods (late childhood, 6–12 years and adolescence, 13–18 years).

Food frequency questionnaire		Coefficients (N, methods^‡^), 95% CIs	
		Age 6–12 years	Age 13–18 years
Animal liver	Frequency	**0.69** (138), 0.57–0.78	**0.57** (139), 0.44–0.67
	Portion	**0.20** (99), -0.19, 0.46^#^	**0.46** (102), 0.29–0.60
Fresh milk	Frequency	**0.70** (139), 0.57–0.79	**0.74** (139), 0.65–0.80
	Portion	**0.65** (70), 0.44–0.79	**0.53** (79), 0.35–0.67
Powdered milk	Frequency	**0.51** (138), 0.31–0.65	**0.44** (139), 0.30–0.57
	Portion	**0.40** (30), -0.28, 0.72^#^	**0.35** (30), 0.01–0.63^†^
Soybean milk	Frequency	**0.73** (139), 0.63–0.81	**0.63** (137), 0.51–0.72
	Portion	**0.51** (82), 0.24–0.69	**0.44** (96), 0.27–0.59
Bean curd	Frequency	**0.73** (139), 0.63–0.81	**0.68** (139), 0.58–0.76
	Portion	**0.51** (82), 0.24–0.69	**0.36** (112), 0.19–0.51
Type of salted fish		**0.28** (140, K), 0.18–0.38	**0.31** (139, K), 0.21–0.41
Mouldy salted fish	Frequency	**0.77** (33), 0.53–0.89	**0.85** (26), 0.68–0.93
	Portion	**0.85** (33), 0.70–0.93	**0.69** (26), 0.43–0.85
Firmed salted fish	Frequency	**0.84** (29), 0.65–0.92	**0.64** (36), 0.40–0.80
	Portion	**0.71** (29), 0.37–0.86	**0.56** (34), 0.28–0.76
Other types of salted fish	Frequency	**0.94** (9), 0.75–0.99	**0.94** (13), 0.81–0.98
	Portion	**0.92** (8), 0.64–0.98	**0.38** (13), -0.22, 0.76^#^
Preserved egg	Frequency	**0.62** (139), 0.47–0.73	**0.62** (139), 0.51–0.72
	Portion	**0.64** (111), 0.47–0.75	**0.48** (112), 0.33–0.61
Preserved vegetable	Frequency	**0.67** (138), 0.54–0.77	**0.59** (139), 0.47–0.69
	Portion	**0.54** (98), 0.31–0.69	**0.47** (114), 0.31–0.60
Preserved fruit	Frequency	**0.61** (138), 0.50–0.71	**0.66** (140), 0.55–0.74
	Portion	**0.61** (81), 0.39–0.75	**0.38** (90), 0.19–0.55

Frequency: Never/Less than once a month/Once a month/1–3 per week/4–6 per week/1–2 per day/3+ per day). Portion: Small/Medium/Large. K: Cohen’s kappa. ^‡^Intra-class correlation coefficient was used in most items, unless otherwise stated. Coefficients: 0 to <0.2, poor; 0.2 to <0.4, fair; 0.4 to <0.6, moderate; 0.6 to <0.8, substantial; and 0.8 to 1.0, almost perfect. No differences by different life periods (difference among coefficients >0.30, and tested by a Fisher Z transformation [p < 0.01]) were found. All the coefficients above were p < 0.01, unless otherwise stated (^†^0.01 ≤ p ≤ 0.05; ^#^p > 0.05 [*-x, x*]).

For other items in all subjects, almost perfect (0.8–1.0) reliability coefficients were observed for the number and sex of siblings and offspring, cancer history, family history of cancer and NPC, smoking and quitting status. Although some FFQ items at age 19–30 and 10 years ago showed poor (0–0.2) coefficients, most factors in the questionnaire had fair-to-substantial reliability coefficients (Supplementary Table s2). Subgroup analyses showed similar reliability coefficients in different strata except for a few items (7 by cases/controls, 14 by sex, 11 by interval between 1^st^ and 2^nd^ questionnaire, 2 by education, and 0 by age at the first questionnaire) (Supplementary Tables s2, s3 and s4).

## Discussion

This test-retest study is the first to report the reliability of a computer-assisted, self-administered questionnaire in an NPC case-control study. We found moderate-to-almost perfect (0.4–1.0) reliability of the data on most NPC aetiology factors in early life periods (Tables 1 and 2, and Fig. 1), and most exposure and factors in the questionnaire had fair-to-substantial (0.2–0.8) reliability (Supplementary Table s2). While the reliability coefficients of most questionnaire items were similar across life periods, the reliability coefficients for indicators of sun exposure were higher 10 years ago than in earlier periods (age 19–30, 13–18 and 6–12). Indeed, sun exposure during childhood is remembered less precisely in general in previous studies on skin cancer^20^. Nevertheless, at least fair (0.2–0.4) reliability of sun exposure in earlier periods was found in our analysis, suggesting that the data are still useful but with limitations.

As some of our questionnaire items were adopted from those in previous NPC case-control studies, our results also suggest that previous case-control study results on the same aetiology factors in similar settings would have similar reliability.

Despite decades of research to find the causal factors of NPC, few studies have reported their reliability of the exposure data^21^. Compared with previous studies on other diseases, our results are consistent in the reliability of different exposures, including food/dietary supplements in the JPHC Study cohort II^22^, body figure in colorectal cancer patients^23^ and sun exposure in melanoma patients^24^. Moderate-to-substantial (0.4–0.8) reliability coefficients were found in most FFQ items at age 6–12 and 13–18, and higher reliability coefficients were observed for frequency than portion size, which are consistent with previous test-retest reliability studies on FFQ^15^.

The test-retest reliability of a questionnaire is essential for assessing the validity of epidemiological studies but seldom reported. Such results are particularly important when exposure data are largely based on recall, as in almost all case-control and many cohort studies, especially for rare cancers such as NPC. Evidence of test-retest reliability of NPC questionnaires has been very limited. We took NPC as an example to reaffirm that assessing the recall errors is fundamental while using epidemiological data, and informing the reliability of a questionnaire is important, especially on early life exposure. We also share our questionnaires online for future references (Supplementary questionnaires).

Our present study included 140 subjects within an NPC case-control study in Hong Kong, showing all the coefficients of each questionnaire items for all subjects and for five subgroups, making it the largest and most comprehensive test-retest reliability analysis on NPC case-control studies in the literature. A computer-assisted questionnaire was used to minimise errors from the interviewer, especially subjective bias. We allowed sufficient time (at least 2 weeks after the first questionnaire: average of 30.5 [standard deviation = 18.8] weeks) between interviews to prevent subjects from simply recalling their responses in the first interview. Moreover, differential recall by cases/controls, sex, time between 1^st^ and 2^nd^ questionnaire (2–29/≥30 weeks), education (secondary or less/postsecondary) or age (25–44/45–59/60+ years) at the first questionnaire was unlikely to be large because most of the reliability coefficients were similar.

A limitation of our reliability study was the relatively low response rate (62.8%), which might affect the representativeness of our findings. Such problem is common in test-retest reliability studies^25^. However, we have found that the respondents had similar basic characteristics as all subjects, suggesting that any non-response bias should be small. Secondly, patients were recruited from two of the five hospitals only, but substantial differences in reliability in subjects from different hospitals were unlikely. However, caution is needed for applying the results to other settings. Because only those who agreed to be re-interviewed were included for assessing test-retest reliability, our results might not be applicable to non-respondents. Thirdly, although our present study is the largest test-retest reliability analysis on NPC case-control studies, small numbers in some rare exposure were observed, including vitamin A supplements (no subject reported ever use), and vitamin D supplements (only 1 reported ever use) and alcohol intake (only 1 reported drinking beer using the one-pint glass). Increasing sample size and revising the questions on rare exposure are recommended. Fourthly, because the short-version (any changes) of FFQ was used at age 19–30, and 10 years ago, the present analysis included only the FFQ at age 6–12 and 13–19. This made it difficult to compare results on FFQ among different life periods. We also found poor reliability coefficients for FFQ items at age 19–30, and 10 years ago. Researchers are recommended to include all periods of exposure on FFQ with the standard version^15^ in future NPC studies.

## Conclusions

This study has shown that the questionnaire data of most NPC aetiology factors of an NPC case-control study in Hong Kong have acceptable reliability. Test-retest reliability study for case-control studies of early life exposure in NPC and other rare cancers is warranted, and the results should be taken into account during data analysis. We recommend that reliability test results should be included for publication of main results from case-control studies, particularly for rare cancers, and the questionnaires should be made available for future references.

## Methods

This test-retest reliability study was a supplementary part of a case-control study in Hong Kong, the main study, on the life-course determinants of NPC^16^. Briefly, eligible subjects for the main study were Chinese aged 18+ in 5 major regional hospitals (QMH, QEH, Pamela Youde Nethersole Eastern Hospital, Princess Margaret Hospital and Tuen Mun Hospital, which treat up to 70% of all NPC cases in Hong Kong) during 2014–2017 without the following conditions: chronic kidney failure, liver cirrhosis, serious heart disease, autoimmune diseases, pregnancy, thyroid disorder, previous thyroid/parathyroid removal surgery, dementia, frailty and cognitive impairment. Cases were incident NPC patients diagnosed with histological and/or radiological evidence in the past 2 months in the Department of Clinical Oncology in the hospitals. Controls were new patients or referrals of a new health complaint in the past 12 months in specialist outpatient clinics, or new inpatients admitted in the past 3 months in the same hospitals who were frequency-matched by age (5-year age groups) and sex to the cases. The controls were selected from patients who attended the clinics or admitted to the hospitals with a wide range of medical diseases unrelated to NPC. Those who had been screened positive for NPC related symptoms such as recent facial nerve palsy, tinnitus, unilateral hearing loss, and epistaxis were excluded. With reference to some questionnaires in previous NPC case-control studies and permission from corresponding authors, we designed a computer-assisted, self-administered questionnaire with 285 questions. A life-course milestone approach was used to collect information on socio-demographics, family cancer history, diet^17^, sun exposure, smoking^18^ and drinking history, occupational, household and others factors.

For the test-retest reliability study, eligible subjects were those who verbally agreed to join at least 2 weeks after completing the first questionnaire, and were invited to be interviewed again during January to December 2016 in two hospitals (QMH and QEH) contributing the largest number of subjects. The present analysis included 140 subjects with a response rate of 62.8% (Supplementary Fig. s1). Respondents and all subjects had similar basic characteristics (Supplementary Table s1). Test-retest reliability was assessed by calculating Kappa (κ), weighted Kappa (κ_w_) and intra-class correlation coefficients (ICCs) for categorical, ordinal and continuous variables, respectively. Reliability coefficients were interpreted according to guidelines from Landis and Koch (0 to 0.2, poor; 0.2 to <0.4, fair; 0.4 to <0.6, moderate; 0.6 to <0.8, substantial; and 0.8 to 1.0, almost perfect)^19^. All analyses were conducted using R 3.3.1.

The present analysis focused on dietary patterns in early life periods, including late childhood (age 6–12) and adolescence (13–18), and on body figure and sunlight exposure in two adulthood periods (age 19–30, and 10 years ago). All items in the questionnaire are shown in Supplementary Table s2. Subgroup analyses were conducted stratified by (1) cases/controls, (2) sex, (3) time between 1^st^ and 2^nd^ questionnaire (2–29/≥30 weeks), (4) education (secondary or less/postsecondary) and (5) age (25–44/45–59/60+ years) at the first questionnaire. The differences between/among coefficients were (1) defined by a difference between/among coefficients of >0.30, and 2) tested by Fisher Z transformation (p < 0.01) with Bonferroni correction to adjust for multiple testing.

All participants gave written, informed consent before participation. The Institutional Review Board of the University of Hong Kong/Hospital Authority Hong Kong West Cluster (UW 11–192) and the Research Ethics Committee of the Hospital Authority Kowloon Central/Kowloon East (KC/KE-13-0115/ER-2) approved the study protocol, and all methods were carried out in accordance with relevant guidelines and regulations.

## Electronic supplementary material

Supplementary materials 1 (figures and tables)

Supplementary materials 2 (questionnaires)
