# Supplementary material for: Test-retest reliability of a computer-assisted self-administered questionnaire on early life exposure in a nasopharyngeal carcinoma case-control study
Source: Sci Rep. 2018 May 4;8:7052. doi: 10.1038/s41598-018-25046-y (PMC5935670; doi:10.1038/s41598-018-25046-y)
Supplement: Supplementary file 1 — Supplementary materials 1 (figures and tables) [file 41598_2018_25046_MOESM1_ESM.pdf]

Supplementary materials 1 (figure and tables)

Test-retest reliability of a computer-assisted self-administered questionnaire on early life exposure in a nasopharyngeal carcinoma case-control study

Zhi-Ming MAI[1,2], Jia-Huang LIN[1,2], Shing-Chun CHIANG[1,2], Roger Kai-Cheong NGAN[2,5], Dora Lai-Wan KWONG[2,4], Wai-Tong NG[2,6], Alice Wan-Ying NG[2,7], Kam-Tong YUEN[2,8], Kai-Ming IP[1,2], Yap-Hang CHAN[2,3], Anne Wing-Mui LEE[2,4], Sai-Yin HO[1*], Maria Li LUNG[2,4] and Tai-Hing LAM[1,2]

[1] School of Public Health, The University of Hong Kong, Hong Kong SAR, China

[2] Centre for Nasopharyngeal Carcinoma Research (CNPCR), Research Grants Council Area of Excellence Scheme, The University of Hong Kong, Hong Kong SAR, China

[3] Department of Medicine, Queen Mary Hospital, The University of Hong Kong, Hong Kong SAR, China

[4] Department of Clinical Oncology, Queen Mary Hospital, The University of Hong Kong, Hong Kong SAR, China

[5] Department of Clinical Oncology, Queen Elizabeth Hospital, Hong Kong SAR, China

[6] Department of Clinical Oncology, Pamela Youde Nethersole Eastern Hospital, Hong Kong SAR, China

[7] Department of Clinical Oncology, Tuen Mun Hospital, Hong Kong SAR, China

[8] Department of Clinical Oncology, Princess Margaret Hospital, Hong Kong SAR, China

Content list

Figure s1. Summary of subjects included in the test-retest reliability analysis, p3
Table s1. Basic characteristics of respondents and all subjects in the Hong Kong multi-centre NPC case-control study, p4
Table s2. Questionnaire items and reliability coefficients with their 95% confidence intervals for all subjects, and by disease status and sex, p5
Table s3. Questionnaire items and reliability coefficients with their 95% confidence intervals by time between questionnaires and education, p23
Table s4. Questionnaire items and reliability coefficients with their 95% confidence intervals by age groups at first questionnaire, p42

Supplementary Figure s1. Summary of subjects included in the test-retest reliability analysis

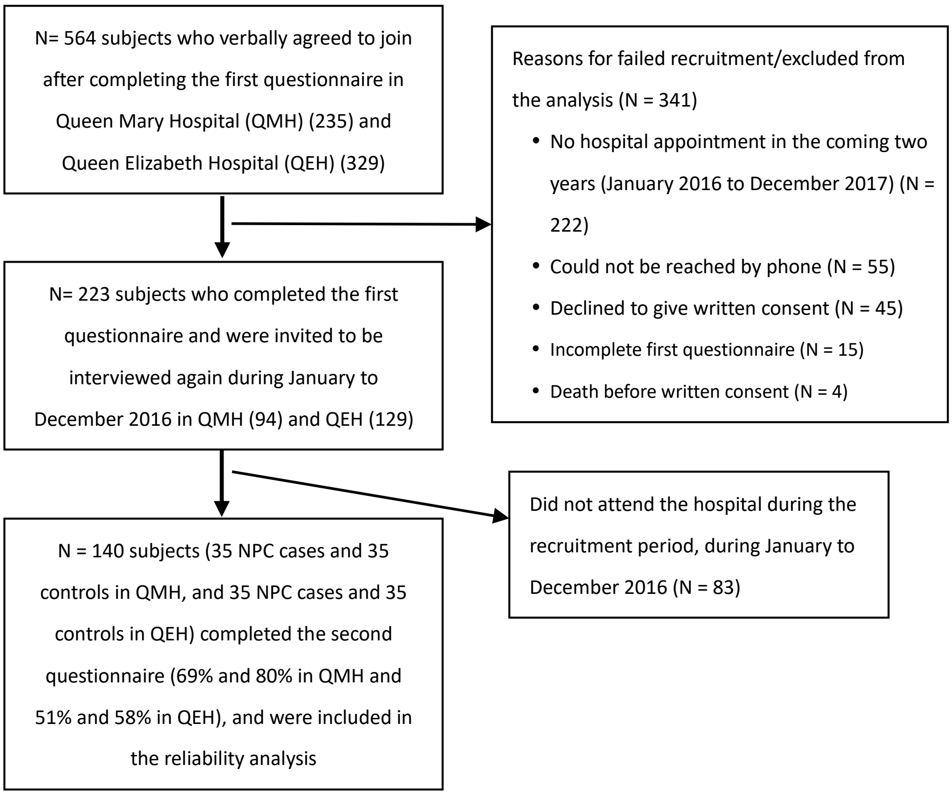

Supplementary Table s1. Basic characteristics of respondents and all subjects in the Hong Kong multi-centre NPC case-control study

	Respondents (n=140) N (%)	All subjects (n=2682) N (%)	P-value†
Mean age (year), SD	51.2, 11.8	50.8, 12.8	0.72
Sex			0.010
Men	110 (78.6)	1830 (68.2)	
Women	30 (21.4)	852 (31.8)	
Education duration, years			0.15
≤6	21 (15.0)	425 (15.8)	
7-12	79 (56.4)	1266 (47.2)	
>12	39 (27.9)	978 (36.5)	
Don't know	1 (0.7)	13 (0.5)	
Household income			0.12
$<15,000	35 (25.0)	644 (24.0)	
$15,000-24,999	47 (33.6)	681 (25.4)	
$25,000-39,999	24 (17.1)	503 (18.8)	
$≥40,000	29 (20.7)	648 (24.2)	
Don't know	5 (3.6)	206 (7.7)	
Smoking status			0.62
Never	91 (65.0)	1637 (61.0)	
Ever	48 (34.3)	1029 (38.4)	
Don't know	1 (0.7)	16 (0.6)	
Drinking status			0.013
Never	73 (52.1)	1370 (51.1)	
Ever	64 (45.7)	1048 (39.1)	
Don't know	3 (2.1)	264 (9.8)	
Salted fish consumption at age 6-12 years			0.44
Never	23 (16.4)	541 (20.2)	
Ever	101 (72.1)	1797 (67.0)	
Don't know	16 (11.4)	344 (12.8)	

† t-test and Chi-square test were used to compare the mean age, and proportions of other factors between respondents and all subjects, respectively.

Supplementary Table s2. Questionnaire items and reliability coefficients with their 95% confidence intervals for all subjects, and by disease status and sex

Questionnaire item	N*	Method	All subjects (N=140)	Disease status				Sex			
					NPC cases (N=70)		Non-NPC controls (N=70)		Men (N=110)		Women (N=30)
				n		n		n		n	
Siblings											
No. of older brother	70	ICC	0.57 (0.39-0.71)	36	0.38 (0.07-0.63)	34	0.90 (0.81-0.95)	54	0.71 (0.55-0.82)	16	0.34 (-0.16, 0.70)
No. of younger brother	68	ICC	0.99 (0.99-1.00)	32	0.99 (0.98-0.99)	36	1.00	52	0.99 (0.99-1.00)	16	0.99 (0.99-1.00)
No. of older sister	70	ICC	0.81 (0.72-0.88)	39	0.75 (0.57-0.86)	31	0.99 (0.97-0.99)	55	0.99 (0.98-0.99)	15	0.64 (0.21-0.86)
No. of younger sister	64	ICC	0.63 (0.46-0.76)	31	0.50 (0.20-0.72)	33	0.97 (0.95-0.99)	51	0.69 (0.51-0.81)	13	0.46 (-0.07, 0.79)
Marital status (single/married/divorced/widowed)	140	K	0.90 (0.83-0.97)	70	0.91 (0.80-1.00)	70	0.89 (0.79-0.99)	110	0.90 (0.82-0.98)	30	0.88 (0.73-1.00)
Income											
Employment status (self-employed/employed/retired/housewives/student/unemployed)	138	K	0.65 (0.54-0.76)	69	0.78 (0.64-0.91)	69	0.53 (0.37-0.69)	109	0.70 (0.59-0.82)	29	0.41 (0.15-0.68)
Personal income (none/less than 15k/15-24.9k/25-39.9k/40k or above)	138	Kw	0.74 (0.61-0.87)	70	0.70 (0.50-0.89)	68	0.77 (0.60-0.94)	108	0.70 (0.55-0.85)	30	0.78 (0.51-1.00)
Household income (none/less than 15k/15-24.9k/25-39.9k/40k or above)	133	Kw	0.61 (0.47-0.76)	67	0.55 (0.32-0.78)	66	0.66 (0.47-0.85)	104	0.62 (0.46-0.78)	29	0.59 (0.27-0.92)
Off-springs											
No. of son	62	ICC	0.99 (0.98-0.99)	28	0.99 (0.97-0.99)	34	1.00	48	0.99 (0.98-0.99)	14	1.00
No. of daughter	59	ICC	1.00	30	1.00	29	1.00	49	1.00	10	1.00
None	19	K	0.95 (0.90-1.00)	21	0.97 (0.92-1.00)	24	0.94 (0.85-1.00)	35	1.00	30	0.93 (0.79-1.00)
Birth place (HK/Macau/Guangxi/Guangdong/Fujian/Hunan/Others/unknown)	140	K	0.94 (0.78-1.00)	70	0.94 (0.85-1.00)	70	0.94 (0.87-1.00)	110	0.92 (0.85-1.00)	30	1.00

Mother's birth place (HK/Macau/Guangxi/Guangdong/Fujian /Hunan/Others/unknown)	140	K	0.76 (0.66-0.85)	70	0.72 (0.58-0.86)	70	0.78 (0.66-0.91)	110	0.78 (0.68-0.88)	30	0.66 (0.45-0.87)
Housing type at 10 years old (Temporary/Public/Home Ownership Scheme/Private (owner)/Private (rent)/Boat/Shanty/Stone/Village/Others)	140	K	0.77 (0.69-0.85)	70	0.79 (0.69-0.90)	70	0.75 (0.63-0.86)	110	0.75 (0.66-0.84)	30	0.83 (0.68-0.98)
Education (None/Old-style school/Primary/Secondary 1-3/Secondary 4-5/Matriculation F.6-7/Technical)											
Subject's	140	K	0.87 (0.81-0.94)	70	0.83 (0.73-0.93)	70	0.91 (0.84-0.99)	110	0.86 (0.79-0.94)	30	0.91 (0.80-1.00)
Subject's father	140	K	0.55 (0.45-0.65)	70	0.58 (0.44-0.72)	70	0.52 (0.38-0.66)	110	0.51 (0.39-0.62)	30	0.73 (0.54-0.92)
Subject's mother	140	K	0.60 (0.58-0.76)	70	0.70 (0.57-0.82)	70	0.65 (0.52-0.78)	110	0.65 (0.54-0.75)	30	0.77 (0.60-0.94)
Cancer history (Yes/No)											
Men	140	K	0.85 (0.75-0.95)	70	0.86 (0.72-0.99)	70	0.85 (0.71-0.99)	110	0.85 (0.75-0.95)	30	-
Women	140	K	0.98 (0.94-1.00)	70	0.95 (0.86-1.00)	70	1.00	110	-	30	0.98 (0.94-1.00)
Mother experienced illness (Yes/No)											
During pregnant	140	K	0.46 (0.32-0.59)	70	0.47 (0.28-0.66)	70	0.43 (0.23-0.62)	110	0.47 (0.31-0.62)	30	0.41 (0.15-0.68)
During delivery	140	K	0.39 (0.23-0.55)	70	0.40 (0.19-0.62)	70	0.37 (0.14-0.61)	110	0.30 (0.12-0.48)	30	0.70 (0.44-0.97)
Birth delivery mode (Natural birth/Caesarean section/Unknown)	140	K	0.41 (0.19-0.62)	70	0.32 (0.02-0.62)$^{+}$	70	0.50 (0.20-0.80)	110	0.41 (0.18-0.65)	30	0.37 (-0.16, 0.89)
Single birth or multiple births	139	K	0.32 (-0.16, 0.81)	70	0.49 (-0.12, 1.00)	69	0.22 (-0.20, 0.62)	109	0.39 (-0.15, 0.93)	30	-
Birth period (Premature/Full-term/Post-term)	140	K	0.66 (0.52-0.80)	70	0.58 (0.38-0.78)	70	0.74 (0.56-0.92)	110	0.58 (0.42-0.75)	30	0.92 (0.77-1.00)
Birth weight (kg)	140	ICC	0.59 (0.47-0.69)	70	0.70 (0.56-0.81)	70	0.42 (0.21-0.60)	110	0.52 (0.37-0.64)	30	0.79 (0.61-0.90)

Breastfed (Yes/No/Unknown)	140	K	0.60 (0.50-0.70)	70	0.62 (0.48-0.76)	70	0.58 (0.43-0.72)	110	0.52 (0.41-0.64)	30	0.89 (0.74-1.00)
Breastfed period (months)	140	ICC	0.39 (0.22-0.56)	70	0.54 (0.28-0.81)	70	0.39 (0.17-0.57)	110	0.38 (0.21-0.53)	30	0.60 (0.32-0.79)
Family history of cancer (Yes/No)	136	K	0.87 (0.78-0.95)	69	0.91 (0.82-1.00)	67	0.82 (0.68-0.96)	106	0.85 (0.75-0.95)	30	0.93 (0.80-1.00)
Family history of nasopharyngeal carcinoma (Yes/No)	75	K	1.00	42	1.00	33	1.00	62	1.00	13	1.00
Oral health											
No. of dental caries	138	Kw	0.57 (0.43-0.71)	69	0.46 (0.26-0.66)	69	0.68 (0.50-0.86)	108	0.61 (0.47-0.76)	30	0.40 (0.04-0.75) †
No. of teeth extracted due to dental caries	92	Kw	0.65 (0.49-0.82)	40	0.81 (0.68-0.93)	52	0.55 (0.29-0.80)	72	0.69 (0.52-0.85)	20	0.48 (-0.01, 0.96)
Periodontal disease (yes/no)	138	K	0.78 (0.67-0.89)	69	0.90 (0.79-1.00)	69	0.67 (0.50-0.85)	108	0.84 (0.73-0.95)	30	0.59 (0.29-0.88)
No. of teeth extracted due to periodontal disease	42	Kw	0.72 (0.51-0.93)	20	0.78 (0.52-1.00)	22	0.65 (0.31-0.98)	34	0.82 (0.69-0.96)	8	0.39 (-0.20, 0.97)
Omega-3											
Ever/never	140	K	0.58 (0.44-0.71)	70	0.71 (0.54-0.87)	70	0.45 (0.24-0.66)	110	0.61 (0.47-0.76)	30	0.43 (0.09-0.76)†
Age started, years	44	ICC	0.60 (0.37-0.76)	22	0.59 (0.23-0.81)	22	0.62 (0.27-0.82)	37	0.52 (0.24-0.72)	7	0.85 (0.36-0.97)
Duration, years	43	ICC	0.59 (0.36-0.76)	22	0.70 (0.40-0.86)	21	0.50 (0.09-0.76) †	36	0.50 (0.21-0.71)	7	0.81 (0.25-0.96)
Frequency (Less than once a month/Once a month/1-3 per week/4-6 per week/1-2 per day/3+ per day)	36	ICC	0.60 (0.34-0.77)	18	0.53 (0.09-0.80) †	18	0.64 (0.26-0.85)	30	0.55 (0.23-0.76)	6	0.83 (0.28-0.97)
Vitamins											
Ever/never	140	K	0.43 (0.27-0.59)	70	0.43 (0.18-0.67)	70	0.39 (0.18-0.60)	110	0.38 (0.20-0.56)	30	0.52 (0.21-0.83)
Multi-vitamins											
Ever/never	140	K	0.55 (0.33-0.78)	70	0.55 (0.11-1.00)	70	0.53 (0.26-0.80)	110	0.53 (0.28-0.79)	30	0.63 (0.16-1.00)
Age started, years	9	ICC	0.59 (-0.08, 0.89)	2	-	7	0.81 (0.30-0.96)	7	0.55 (-0.28, 0.90)	2	0.92 (-0.22, 1.00)
Duration, years	9	ICC	1.00 (0.99-1.00)	2	-	7	1.00	7	0.99 (0.99-1.00)	2	0.99 (0.34-1.00)
Frequency	9	ICC	0.64 (0.01-0.91) †	2	-	7	0.58 (-0.16, 0.91)	7	0.58 (-0.16, 0.91)	2	0.92 (-0.22, 1.00)

(Less than once a month/Once a month/1-3 per week/4-6 per week/1-2 per day/3+ per day)										
Vitamins A										
Ever/never	140	K	-	70	-	70	-	110	-	30	-
Age started, years	0	ICC	-	0	-	0	-	0	-	0	-
Duration, years	0	ICC	-	0	-	0	-	0	-	0	-
Frequency	0	ICC	-	0	-	0	-	0	-	0	-
(Less than once a month/Once a month/1-3 per week/4-6 per week/1-2 per day/3+ per day)										
Vitamins B										
Ever/never	140	K	0.48 (0.22-0.74)	70	0.31 (-0.19, 0.80)	70	0.53 (0.23-0.84)	110	0.20 (-0.16, 0.56)	30	0.63 (0.30-0.96)
Age started, years	6	ICC	0.36 (-0.39, 0.87)	1	-	5	0.40 (-0.63, 0.92)	1	-	5	0.29 (-0.20, 0.86)
Duration, years	6	ICC	0.83 (0.19-0.97)	1	-	5	0.88 (0.36-0.99)	1		5	0.92 (0.48-0.99)
Frequency	5	ICC	0.43 (-0.51, 0.92)	1	-	4	0.39 (-0.66, 0.94)	1		5	0.43 (-0.51, 0.92)
(Less than once a month/Once a month/1-3 per week/4-6 per week/1-2 per day/3+ per day)										
Vitamins C										
Ever/never	140	K	0.34 (0.11-0.56)	70	0.21 (-0.11, 0.54)	70	0.42 (0.13-0.71)	110	0.30 (0.02-0.57) †	30	0.39 (0.02-0.77) †
Age started, years	7	ICC	0.58 (0.08-0.91) †	2	-	5	0.50 (-0.21, 0.92)	4	0.79 (-0.09, 0.99)	3	-0.79 (-1.15, 0.80)
Duration, years	7	ICC	0.23 (0.56-0.81)	2	-	5	0.99 (0.98-1.00)	4	-0.09 (-0.91, 0.86)	3	1.00 (0.39-1.00)
Frequency	7	ICC	0.88 (0.38-0.98)	2	-	5	0.91 (0.43-0.99)	4	0.76 (-0.11, 0.98)	3	1.00
(Less than once a month/Once a month/1-3 per week/4-6 per week/1-2 per day/3+ per day)										
Vitamins D										

Ever/never consumed	140	K	0.73 (0.51-0.93)	70	0.78 (0.52-1.00)	70	0.65 (0.31-0.98)	110	0.82 (0.69-0.96)	30	0.62 (0.40, 0.87)
Age started, years	1	ICC	-	0	-	1	-	0	-	1	-
Duration, years	1	ICC	-	0	-	1	-	0	-	1	-
Frequency (Less than once a month/Once a month/1-3 per week/4-6 per week/1-2 per day/3+ per day)	1	ICC	-	0	-	1	-	0	-	1	-
Vitamins E											
Ever/never	140	K	0.60 (0.31-0.89)	70	0.73 (0.38-1.00)	70	0.47 (0.03-0.91) +	110	0.48 (0.05-0.91) +	30	0.71 (0.35-1.00)
Age started, years	5	ICC	0.50 (0.20-0.92)	3	-	2	-	2	-	3	0.35 (-0.19, 0.97)
Duration, years	5	ICC	0.92 (0.37-0.99)	3	-	2	-	2	-	3	0.94 (0.01-1.00) +
Frequency (Less than once a month/Once a month/1-3 per week/4-6 per week/1-2 per day/3+ per day)	5	ICC	0.86 (0.08-0.99) +	3	-	2	-	2	-	3	1.00
Active smoking											
Ever/never	139	K	0.92 (0.85-0.99)	70	0.97 (0.91-1.00)	69	0.87 (0.76-0.99)	109	0.91 (0.83-0.99)	30	1.00
Time between wake up and first cig (Less than 5/6-30/31-60/60+ minutes)	140	Kw	0.80 (0.68-0.91)	70	0.86 (0.73-0.99)	70	0.75 (0.57-0.93)	110	0.80 (0.68-0.92)	30	0.65 (0.02-1.00) +
Age starting years	46	ICC	0.80 (0.66-0.96)	23	0.88 (0.74-0.95)	23	0.58 (0.23-0.80)	44	0.80 (0.64-0.96)	2	-
Smoking amount											
Before age of 18 years	24	ICC	0.58 (0.24-0.79)	8	0.58 (-0.09, 0.90)	16	0.59 (0.14-0.84)	22	0.71 (0.43-0.87)	2	-2.57 (-14.87, 0.99)
After age of 18 years	46	ICC	0.72 (0.54-0.83)	23	0.87 (0.71-0.94)	23	0.63 (0.30-0.82)	44	0.72 (0.54-0.84)	2	-4.00 (-9.00, 0.97)
Age at quitting	32	ICC	1.00 (0.99-1.00)	18	0.99 (0.99-1.00)	14	0.99 (0.97-1.00)	31	0.99 (0.98-1.00)	1	-
Duration of quitting	24	ICC	0.84 (0.69-0.93)	13	0.52 (0.01-0.82) +	11	0.99 (0.98-0.99)	23	0.84 (0.66-0.93)	1	-

Passive smoking (*household*)											
**childhood**											
No. of co-living smokers	138	ICC	0.77 (0.69-0.83)	70	0.80 (0.70-0.87)	68	0.74 (0.60-0.83)	109	0.71 (0.60-0.80)	29	0.94 (0.87-0.97)
Co-living smoker 1											
Relationship (Father/Mother/Grandfather/Grandmother/Maternal grandfather/Maternal grandmother/Siblings/Others)	82	K	0.60 (0.42-0.79)	42	0.56 (0.28-0.83)	40	0.66 (0.42-0.90)	64	0.63 (0.43-0.84)	18	0.52 (0.15-0.89)
Exposed time/day (Less than 5 minutes/30-60 minutes/1-2 hours/2+ hours)	81	Kw	0.55 (0.36-0.73)	42	0.50 (0.22-0.78)	39	0.57 (0.30-0.83)	63	0.49 (0.26-0.72)	18	0.74 (0.51-0.96)
Years of co-living	77	ICC	0.30 (0.09-0.49) †	40	-0.02 (-0.31, 0.28)	37	0.42 (0.11-0.65)	60	0.32 (0.08-0.52) †	17	0.23 (-0.27,0.63)
Co-living smoker 2											
Relationship (Father/Mother/Grandfather/Grandmother/Maternal grandfather/Maternal grandmother/Siblings/Others)	23	K	0.62 (0.38-0.85)	14	0.63 (0.34-0.93)	9	0.55 (0.18-0.92)	15	0.68 (0.42-0.94)	8	0.53 (0.20-0.85)
Exposed time/day (Less than 5 minutes/30-60 minutes/1-2 hours/2+ hours)	23	Kw	0.44 (0.07-0.82) †	14	0.27 (-0.17, 0.71)	9	0.35 (-0.17, 0.87)	15	0.31 (-0.20, 0.82)	8	0.71 (0.46-0.97)
Years of co-living	23	ICC	0.39 (0.02-0.68) †	14	0.40 (-0.15, 0.76)	9	0.37 (-0.20, 0.80)	15	0.46 (-0.01, 0.77)	8	-0.08 (-0.69, 0.61)
Co-living smoker 3											
Relationship (Father/Mother/Grandfather/Grandmother/Maternal grandfather/Maternal grandmother/Siblings/Others)	8	K	1.00	5	1.00	3	1.00	6	1.00	2	-

Exposed time/day (Less than 5 minutes/30-60 minutes/1-2 hours/2+ hours)	8	Kw	0.36 (-0.29, 1.00)	5	0.75 (0.55-0.94)	3	-0.14 (-0.83, 0.56)	6	0.25 (-0.52, 1.00)	2	0.67 (0.67-0.67)
Years of co-living	8	ICC	0.99 (0.94-1.00)	5	0.95 (0.69-1.00)	3	1.00	6	0.99 (0.92-1.00)	2	-
**adulthood**											
No. of co-living smokers	137	ICC	0.70 (0.60-0.77)	70	0.79 (0.69-0.87)	67	0.57 (0.38-0.71)	108	0.70 (0.59-0.79)	29	0.66 (0.40-0.83)
Co-living smoker 1											
Relationship (Father/Mother/Grandfather/Grandmother/Maternal grandfather/Maternal grandmother/Siblings/Others)	65	K	0.69 (0.52-0.85)	37	0.65 (0.43-0.88)	28	0.73 (0.49-0.98)	46	0.65 (0.44-0.86)	19	0.74 (0.48-1.00)
Exposed time/day (Less than 5 minutes/30-60 minutes/1-2 hours/2+ hours)	63	Kw	0.37 (0.13-0.61)	35	0.29 (-0.06, 0.64)	28	0.41 (0.05-0.76) ┼	44	0.25 (-0.05, 0.56)	19	0.67 (0.39-0.95)
Years of co-living	64	ICC	0.67 (0.51-0.79)	36	0.60 (0.35-0.78)	28	0.75 (0.54-0.88)	45	0.74 (0.58-0.85)	19	0.58 (0.17-0.82)
Co-living smoker 2											
Relationship (Father/Mother/Grandfather/Grandmother/Maternal grandfather/Maternal grandmother/Siblings/Others)	21	K	0.63 (0.39-0.86)	14	0.70 (0.42-0.98)	7	0.32 (-0.12, 0.77)	16	0.67 (0.41-0.93)	5	0.47 (0.09-0.85) ┼
Exposed time/day (Less than 5 minutes/30-60 minutes/1-2 hours/2+ hours)	21	Kw	0.35 (-0.05, 0.74)	14	0.56 (0.23-0.88)	7	0.19 (-0.20, 0.57)	16	0.15 (-0.27, 0.56)	5	0.91 (0.87-0.94)
Years of co-living	21	ICC	0.53 (0.15-0.77)	14	0.75 (0.41-0.91)	7	-0.37 (-0.88, 0.48)	16	0.29 (-0.16, 0.67)	5	0.69 (-0.49, 0.96)
Co-living smoker 3											
Relationship (Father/Mother/Grandfather/Grandmot	4	K	-	3	1.00	1	-	3	-	1	-

her/Maternal grandfather/Maternal grandmother/Siblings/Others)										
Exposed time/day (Less than 5 minutes/30-60 minutes/1-2 hours/2+ hours)	4	Kw	0.31 (-0.41, 1.00)	3	0.89 (0.81-0.97)	1	-	3	-	1	-
Years of co-living	4	ICC	0.87 (-0.25, 0.99)	3	0.88 (-1.23, 0.99)	1	-	3	0.83 (-1.02, 0.99)	1	-
Passive smoking (*workplace*)											
**childhood (before 18 years old)**											
Ever/never	138	K	0.28 (0.16-0.41)	70	0.26 (0.09-0.43) †	68	0.30 (0.12-0.48)	109	0.25 (0.11, 0.39)	29	0.38 (0.12-0.64)
Time of exposed/day (Less than 5 minutes/30-60 minutes/1-2 hours/2+ hours)	23	Kw	0.54 (0.21-0.87)	13	0.46 (-0.01, 0.94)	10	0.62 (0.19-1.00)	17	0.23 (-0.28, 0.74)	6	0.74 (0.54-0.94)
How long did you work that exposed to passive smoking, years	23	ICC	0.53 (0.15-0.77)	13	0.68 (0.25-0.89)	10	0.39 (-0.20, 0.79)	17	0.49 (0.03-0.78) †	6	0.44 (-0.49, 0.90)
**adulthood (after 18 years old)**											
Ever/never	136	K	0.55 (0.41-0.69)	69	0.62 (0.42-0.82)	67	0.48 (0.28-0.69)	107	0.57 (0.40-0.73)	29	0.42 (0.11-0.72)
Time of exposed/day (Less than 5 minutes/30-60 minutes/1-2 hours/2+ hours)	77	Kw	0.45 (0.26-0.65)	43	0.42 (0.12-0.71)	34	0.49 (0.24-0.74)	69	0.44 (0.22-0.65)	8	0.50 (0.09-0.90) †
How long did you work that exposed to passive smoking, years	76	ICC	0.56 (0.39-0.70)	43	0.68 (0.48-0.81)	33	0.35 (0.00-0.61) †	67	0.54 (0.35-0.69)	9	0.59 (-0.10, 0.89)
Occupational hazards											
Exposed/non-exposed	139	K	0.63 (0.51-0.76)	70	0.46 (0.25-0.67)	69	0.81 (0.67-0.94)	109	0.66 (0.52-0.80)	30	0.52 (0.21-0.83)
Dust	139	K	0.66 (0.53-0.78)	70	0.49 (0.28-0.71)	69	0.80 (0.66-0.94)	110	0.63 (0.49-0.77)	30	0.73 (0.45-1.00)
Chemical	139	K	0.32 (0.16-0.49)	70	0.24 (0.03-0.46) †	69	0.42 (0.19-0.66)	110	0.24 (0.05-0.43)	30	0.58 (0.31-0.86)
Fumes	139	K	0.54 (0.38-0.70)	70	0.50 (0.27-0.74)	69	0.58 (0.37-0.78)	110	0.56 (0.39-0.73)	30	0.30 (-0.16, 0.76)
Acid or alkali	139	K	0.54 (0.32-0.77)	70	0.18 (-0.10, 0.45)	69	0.92 (0.75-1.00)	110	0.57 (0.30-0.83)	30	0.46 (0.04-0.87) †

Alcohol drinking												
Ever/never	136	K	0.63 (0.50-0.76)	68	0.50 (0.29-0.70)	68	0.77 (0.61-0.92)	106	0.62 (0.47-0.77)	30	0.57 (0.26-0.88)
Ever drink once per month	48	K	0.63 (0.41-0.84)	22	0.43 (0.07-0.79) ⁺	26	0.75 (0.49-1.00)	44	0.64 (0.41-0.86)	4	0.50 (-0.23, 1.00)
Age at starting	53	ICC	0.59 (0.39-0.74)	25	0.90 (0.78-0.95)	28	0.30 (-0.07, 0.60)	48	0.64 (0.43-0.78)	5	0.02 (-0.54, 0.78)
Age at stopping	19	ICC	0.96 (0.90-0.99)	11	0.98 (0.93-0.99)	8	0.94 (0.71-0.99)	17	0.95 (0.87-0.98)	2	1.00
Duration of stopping drinking	4	ICC	1.00 (0.94-1.00)	1	-	3	0.99 (0.95-1.00)	3	0.99 (0.76-1.00)	1	-
Red wine											
Frequency (Less than once a month/Once a month/1-3 per week/4-6 per week/1-2 per day/3+ per day)	23	ICC	0.66 (0.34-0.84)	12	0.59 (0.07-0.86) ⁺	11	0.70 (0.24-0.91)	20	0.49 (0.06-0.76) ⁺	3	0.86 (-0,11, 1.00)
Portion, glasses	16	ICC	0.41 (-0.11, 0.75)	8	0.56 (-0.16, 0.89)	8	0.37 (-0.51, 0.84)	14	0.32 (-0.25, 0.72)	2	0.92 (-0.22, 1.00)
white wine											
Frequency (Less than once a month/Once a month/1-3 per week/4-6 per week/1-2 per day/3+ per day)	7	ICC	0.73 (0.08-0.95) ⁺	4	0.70 (-0.90, 0.98)	3	0.80 (-0.29, 0.99)	5	0.73 (-0.38, 0.97)	2	0.80 (-0.63, 1.00)
Portion, glasses	7	ICC	0.53 (-0.40, 0.91)	4	0.66 (-0.35, 0.97)	3	0.50 (-0.71, 0.98)	5	0.50 (-0.84, 0.94)	2	1.00
beer											
Frequency (Less than once a month/Once a month/1-3 per week/4-6 per week/1-2 per day/3+ per day)	47	ICC	0.68 (0.49-0.81)	23	0.47 (0.07-0.73) ⁺	24	0.79 (0.58-0.91)	44	0.69 (0.49-0.82)	3	0.50 (-0.71, 0.98)
Portion (unit: pint)	1	ICC	-	0	-	1	-	1	-	1	-
Portion (unit: 375ml/can)	8	ICC	0.43 (-0.40, 0.86)	4	0.19 (-1.08, 0.92)	4	0.92 (0.43-0.99)	5	0.37 (-0.74, 0.91)	3	0.00 (-0.95, 0.95)
Portion (unit: 500ml/can)	5	ICC	0.23 (-1.01, 0.89)	1	-	0	-	5	0.23 (-1.01, 0.89)	0	-
Portion (unit: 330ml/bottle)	3	ICC	0.65 (-0.56, 0.99)	1	-	2	-	3	0.65 (-0.56, 0.99)	0	-
Portion (unit: 600ml/bottle)	6	ICC	0.22 (-0.37, 0.81)	4	0.00 (-1.05, 0.89)	2	-	6	0.22 (-0.37, 0.81)	0	-
spirits											

Frequency (Less than once a month/Once a month/1-3 per week/4-6 per week/1-2 per day/3+ per day)	14	ICC	0.50 (-0.03, 0.81)	7	-0.74 (-1.23, 0.24)	7	0.47 (-0.40, 0.89)	12	0.59 (0.03-0.87) ┼	2	0.00 (-1.00,1.00)
Portion (unit: Chinese-style glass)	2	ICC	-	0	-	2	-	1	-	1	-
Portion (unit: Western-style glass)	4	ICC	0.99 (0.94-1.00)	0	-	4	0.99 (0.94-1.00)	2	1.00	2	0.99 (0.63-1.00)
Sun exposure											
10 years ago											
Outdoor exercise (Less than once a month/Once a month/1-3 per week/4-6 per week/Daily)	140	Kw	0.70 (0.59-0.81)	70	0.72 (0.58-0.86)	70	0.68 (0.50-0.85)	110	0.69 (0.56-0.81)	30	0.74 (0.53-0.95)
Duration of sun exposure (Less than 1/2-4/5-7/8-10 hours)	140	Kw	0.89 (0.84-0.94)	70	0.92 (0.87-0.97)	70	0.85 (0.77-0.94)	110	0.88 (0.82-0.94)	30	0.89 (0.79-0.99)
Avoid sun exposure (Never/Ever)	140	K	0.48 (0.38-0.58)	70	0.39 (0.25-0.53)	70	0.57 (0.43-0.71)	110	0.44 (0.33-0.56)	30	0.61 (0.40-0.81)
Protection from sunshine (Never/Ever)	140	K	0.52 (0.42-0.62)	70	0.49 (0.35-0.64)	70	0.54 (0.40-0.67)	110	0.53 (0.42-0.65)	30	0.44 (0.22-0.65)
19-30 years ago											
Outdoor exercise (Less than once a month/Once a month/1-3 per week/4-6 per week/Daily)	138	Kw	0.46 (0.32-0.61)	70	0.50 (0.31-0.68)	68	0.40 (0.17-0.63)	108	0.48 (0.32-0.65)	30	0.38 (0.06-0.71) ┼
Duration of sun exposure (Less than 1/2-4/5-7/8-10 hours)	138	Kw	0.46 (0.29-0.63)	70	0.59 (0.39-0.79)	68	0.32 (0.06-0.58) ┼	108	0.51 (0.33-0.69)	30	0.19 (-0.12, 0.49)
Avoid sun exposure (Never/Ever)	139	K	0.27 (0.15-0.38)	70	0.19 (0.05-0.34) ┼	69	0.36 (0.19-0.52)	109	0.26 (0.13-0.40)	30	0.26 (0.03-0.48) ┼

Protection from sunshine (Never/Ever)	139	K	0.26 (0.14-0.37)	70	0.24 (0.08-0.41) †	69	0.25 (0.10-0.41)	109	0.22 (0.08-0.35) †	30	0.28 (0.04-0.51) †
13-18 years ago											
Outdoor exercise (Less than once a month/Once a month/1-3 per week/4-6 per week/Daily)	138	Kw	0.52 (0.37-0.67)	70	0.50 (0.29-0.72)	68	0.54 (0.33-0.75)				
Duration of sun exposure (Less than 1/2-4/5-7/8-10 hours)	138	Kw	0.55 (0.38-0.71)	70	0.65 (0.48-0.82)	68	0.43 (0.16-0.71)	108	0.49 (0.30-0.68)	30	0.57 (0.30-0.84)
Avoid sun exposure (Never/Ever)	139	K	0.42 (0.25-0.59)	70	0.21 (0.04-0.39) †	69	0.45 (0.28-0.62)	108	0.58 (0.42-0.73)	30	0.45 (0.01-0.89) †
Protection from sunshine (Never/Ever)	139	K	0.31 (0.14-0.49)	70	0.25 (0.09-0.41) †	69	0.22 (0.04-0.39) †	109	0.32 (0.18-0.46)	30	0.38 (0.14-0.63)
6-12 years ago								109	0.21 (0.07-0.36) †	30	0.22 (0.00-0.45) †
Outdoor exercise (Less than once a month/Once a month/1-3 per week/4-6 per week/Daily)	137	Kw	0.46 (0.30-0.62)	69	0.42 (0.19-0.65)	68	0.52 (0.31-0.72)	108	0.44 (0.25-0.62)	29	0.53 (0.22-0.85)
Duration of sun exposure (Less than 1/2-4/5-7/8-10 hours)	137	Kw	0.32 (0.13-0.51)	70	0.41 (0.16-0.67)	67	0.22 (-0.05, 0.50)	107	0.41 (0.20-0.61)	30	0.09 (-0.24, 0.43)
Avoid sun exposure (Never/Ever)	139	K	0.27 (0.15-0.40)	70	0.29 (0.12-0.46)	69	0.25 (0.07-0.44) †	109	0.24 (0.09-0.38) †	30	0.41 (0.14-0.68)
Protection from sunshine (Never/Ever)	139	K	0.30 (0.15-0.44)	70	0.28 (0.09-0.47) †	69	0.29 (0.08-0.50) †	109	0.27 (0.10-0.43)	30	0.39 (0.10-0.68)
Skin tone											
Current											
Face	138	Kw	0.57 (0.43-0.70)	69	0.51 (0.31-0.70)	69	0.63 (0.47-0.79)	108	0.58 (0.44-0.71)	30	0.50 (0.11-0.88)
Hand	139	Kw	0.47 (0.31-0.64)	70	0.45 (0.23-0.68)	69	0.49 (0.25-0.73)	109	0.45 (0.26-0.64)	30	0.52 (0.13-0.91)
10 years ago: hand	91	Kw	0.53 (0.40-0.67)	44	0.40 (0.16-0.65)	47	0.63 (0.49-0.78)	72	0.52 (0.37-0.66)	19	0.35 (-0.08, 0.78)

Age 19-30 years: hand		138	Kw	0.50 (0.37-0.62)	69	0.50 (0.33-0.68)	69	0.48 (0.29-0.66)	109	0.51 (0.37-0.64)	29	0.29 (-0.02, 0.60)
Age 13-18 years: hand		137	Kw	0.51 (0.39-0.62)	68	0.39 (0.20-0.58)	69	0.59 (0.45-0.72)	108	0.48 (0.35-0.61)	29	0.59 (0.35-0.82)
Age 6-12 years: hand		132	Kw	0.56 (0.44-0.69)	69	0.50 (0.31-0.70)	63	0.61 (0.45-0.78)	105	0.61 (0.48-0.74)	27	0.39 (0.13-0.65)
Body figure, height and weight												
**current**												
Body figure: male		105	Kw	0.68 (0.57-0.78)	53	0.59 (0.39-0.79)	52	0.74 (0.62-0.85)	105	0.68 (0.57-0.78)	-	-
Body figure: female		28	Kw	0.77 (0.62-0.92)	14	0.79 (0.64-0.94)	14	0.74 (0.48-0.99)	-	-	28	0.77 (0.62-0.92)
Height, cm		130	ICC	0.95 (0.93-0.96)	65	0.96 (0.93-0.97)	65	0.94 (0.89-0.96)	104	0.92 (0.89-0.95)	26	0.93 (0.85-0.97)
Weight, kg		137	ICC	0.91 (0.79-0.95)	70	0.86 (0.28-0.95)	67	0.98 (0.97-0.99)	107	0.90 (0.77-0.95)	30	0.91 (0.69-0.97)
**10 years ago**												
Body figure: male		78	Kw	0.63 (0.50-0.76)	40	0.56 (0.40-0.71)	38	0.67 (0.49-0.85)	78	0.63 (0.50-0.76)	-	-
Body figure: female		19	Kw	0.65 (0.46-0.84)	9	0.77 (0.65-0.89)	10	0.60 (0.24-0.96)	-	-	19	0.65 (0.46-0.84)
Weight, kg		96	ICC	0.33 (0.14-0.50)	49	0.18 (-0.10, 0.44)	47	0.42 (0.15-0.63)	75	0.33 (0.12-0.52)	21	0.23 (-0.15, 0.58)
**Age 19-30 years**												
Body figure: male		109	Kw	0.56 (0.41-0.72)	56	0.64 (0.47-0.82)	53	0.50 (0.26-0.73)	109	0.56 (0.41-0.72)	--	--
Body figure: female		28	Kw	0.76 (0.61-0.92)	14	0.77 (0.57-0.97)	14	0.76 (0.51-1.00)	-	-	28	0.76 (0.61-0.92)
**Age 13-18 years**												
Body figure: male		110	Kw	0.68 (0.54-0.82)	56	0.66 (0.45-0.87)	54	0.67 (0.48-0.86)	110	0.68 (0.54-0.82)	-	-
Body figure: female		28	Kw	0.66 (0.45-0.87)	14	0.75 (0.52-0.97)	14	0.60 (0.34-0.86)	-	-	28	0.66 (0.45-0.87)
**Age 6-12 years**												
Body figure: male		108	Kw	0.69 (0.58-0.81)	55	0.66 (0.50-0.82)	53	0.71 (0.55-0.87)	108	0.69 (0.58-0.81)	-	-
Body figure: female		28	Kw	0.86 (0.75-0.96)	14	0.86 (0.71-1.00)	14	0.81 (0.63-0.99)	-	-	28	0.86 (0.75-0.96)
**FFQ at age 6-12 years**												
All meat	freq	137	ICC	0.67 (0.52-0.77)	68	0.66 (0.45-0.79)	69	0.68 (0.43-0.82)	107	0.64 (0.47-0.76)	30	0.73 (0.42-0.87)
	port	131	ICC	0.46 (0.23-0.61)	65	0.70 (0.51-0.82)	66	0.18 (-0.34, 0.49)	105	0.49 (0.25-0.66)	26	0.29 (-0.50, 0.67)
Animal liver	freq	138	ICC	0.69 (0.57-0.78)	69	0.72 (0.55-0.83)	69	0.66 (0.46-0.79)	108	0.74 (0.62-0.82)	30	0.48 (-0.11, 0.76)
	port	99	ICC	0.20 (-0.19, 0.46)	49	0.34 (-0.18, 0.63)	50	0.06 (-0.62, 0.46)	84	0.23 (-0.18, 0.50)	15	-0.24 (-3.23, 0.60)
All fish	freq	138	ICC	0.67 (0.54-0.77)	69	0.72 (0.55-0.83)	69	0.60 (0.36-0.75)	108	0.69 (0.54-0.79)	30	0.59 (0.14-0.81)

	port	136	ICC	0.46 (0.24-0.61)	67	0.43 (0.08-0.65) ┼	69	0.46 (0.12-0.66)	107	0.46 (0.22-0.63)	29	0.46 (-0.14, 0.74)
All fruit	freq	138	ICC	0.75 (0.66-0.82)	69	0.74 (0.58-0.84)	69	0.75 (0.60-0.85)	108	0.79 (0.69-0.85)	30	0.66 (0.30-0.84)
	port	119	ICC	0.56 (0.36-0.69)	56	0.66 (0.42-0.80)	63	0.45 (0.09-0.67) ┼	92	0.61 (0.40-0.74)	27	0.38 (-0.39, 0.72)
All vegetable	freq	139	ICC	0.75 (0.64-0.83)	70	0.75 (0.59-0.84)	69	0.76 (0.60-0.85)	109	0.75 (0.61-0.84)	30	0.73 (0.44-0.87)
	port	136	ICC	0.61 (0.45-0.72)	68	0.61 (0.36-0.76)	68	0.59 (0.34-0.75)	106	0.54 (0.32-0.68)	30	0.76 (0.49-0.88)
Fresh milk	freq	139	ICC	0.70 (0.57-0.79)	70	0.71 (0.52-0.82)	69	0.70 (0.51-0.81)	109	0.69 (0.50-0.81)	30	0.76 (0.49-0.88)
	port	70	ICC	0.65 (0.44-0.79)	34	0.69 (0.37-0.84)	36	0.59 (0.18-0.79)	57	0.70 (0.49-0.82)	13	0.54 (-0.30, 0.85)
Powdered milk	freq	138	ICC	0.51 (0.31-0.65)	69	0.55 (0.27-0.72)	69	0.47 (0.14-0.67)	109	0.58 (0.39-0.72)	29	0.03 (-1.12, 0.55)
	port	30	ICC	0.40 (-0.28, 0.72)	16	0.43 (-0.64, 0.80)	14	0.38 (-0.73, 0.79)	27	0.39 (-0.35, 0.73)	3	0.67 (-4.53, 0.99)
Soybean milk	freq	139	ICC	0.73 (0.63-0.81)	70	0.68 (0.48-0.80)	69	0.78 (0.65-0.87)	109	0.77 (0.66-0.84)	30	0.63 (0.22-0.83)
	port	82	ICC	0.51 (0.24-0.69)	40	0.58 (0.19-0.78)	42	0.37 (-0.19, 0.66)	64	0.46 (0.11-0.67)	18	0.66 (0.11-0.87)
Type of salted fish	freq	140	K	0.28 (0.18-0.38)	70	0.35 (0.21-0.49)	70	0.21 (0.08-0.34) ┼	110	0.27 (0.16-0.38)	30	0.30 (0.10-0.50)
Mouldy salted fish	port	33	ICC	0.77 (0.53-0.89)	20	0.79 (0.47-0.92)	13	0.44 (-1.05, 0.84)	26	0.79 (0.54-0.91)	7	0.71 (-0.36, 0.95)
	freq	33	ICC	0.85 (0.70-0.93)	20	0.89 (0.72-0.96)	13	0.44 (-0.96, 0.83)	26	0.88 (0.73-0.95)	7	-0.00 (-2.23, 0.80)
Firmed salted fish	port	29	ICC	0.84 (0.65-0.92)	17	0.90 (0.56-0.97)	12	0.23 (-2.26, 0.79)	21	0.89 (0.73-0.96)	8	-0.56 (-6.00,0.82)
	freq	29	ICC	0.71 (0.37-0.86)	17	0.60 (-0.16, 0.86)	12	0.90 (0.63-0.97)	21	0.88 (0.70-0.95)	8	-0.62 (-65.8, 0.73)
Other types of salted fish	port	0	ICC	-	0	-	0	-	0	-	0	-
	freq	0	ICC	-	0	-	0	-	0	-	0	-
Any types of salted fish	port	9	ICC	0.94 (0.75-0.99)	3	0.92 (-0.24, 0.99)	6	0.94 (0.13-0.99)	7	0.95 (0.75-0.99)	2	0.89 (-3.32, 1.00)
	freq	8	ICC	0.92 (0.64-0.98)	2	-	6	0.91 (0.44-0.99)	6	0.91 (0.44-0.99)	2	-
Preserved meat	port	137	ICC	0.65 (0.52-0.75)	68	0.66 (0.45-0.79)	69	0.64 (0.42-0.78)	107	0.58 (0.38-0.71)	30	0.77 (0.51-0.89)
	freq	110	ICC	0.67 (0.52-0.78)	54	0.71 (0.50-0.83)	56	0.60 (0.32-0.77)	93	0.66 (0.48-0.77)	17	0.78 (0.40-0.92)
Preserved egg	port	139	ICC	0.62 (0.47-0.73)	69	0.64 (0.42-0.78)	70	0.60 (0.36-0.75)	109	0.66 (0.50-0.77)	30	0.50 (-0.06, 0.76)
	freq	111	ICC	0.64 (0.47-0.75)	50	0.75 (0.57-0.86)	61	0.53 (0.22-0.72)	90	0.58 (0.36-0.72)	21	0.89 (0.72-0.95)
Preserved vegetable	port	138	ICC	0.67 (0.54-0.77)	68	0.61 (0.36-0.76)	70	0.74 (0.58-0.84)	108	0.61 (0.43-0.73)	30	0.80 (0.59-0.91)
	freq	98	ICC	0.54 (0.31-0.69)	46	0.58 (0.24-0.77)	52	0.49 (0.11-0.71)	76	0.40 (0.06-0.62) ┼	22	0.79 (0.49-0.91)
Preserved fruit	port	138	ICC	0.61 (0.50-0.71)	69	0.52 (0.32-0.67)	69	0.72 (0.58-0.81)	108	0.56 (0.41-0.68)	30	0.73 (0.50-0.86)
	freq	81	ICC	0.61 (0.39-0.75)	37	0.54 (0.10-0.76)	44	0.65 (0.35-0.81)	66	0.60 (0.34-0.75)	15	0.58 (-0.14, 0.86)

**FFQ at age 13-18 years**											
Frequency of deep-fried food (Never/Less than once a month/Once a month/1-3 per week/4-6 per week/1-2 per day/3+ times per day)		140	Kw	0.30 (0.08-0.52) ⸸	70	0.23 (-0.13, 0.59)	70	0.38 (0.17-0.58)	110	0.29 (0.02-0.56) ⸸	30	0.27 (-0.10, 0.64)
Frequency of barbeque meat (Never/Less than once a month/Once a month/1-3 per week/4-6 per week/1-2 per day/3+ times per day)		140	Kw	0.52 (0.39-0.65)	70	0.46 (0.28-0.64)	70	0.59 (0.40-0.77)	110	0.47 (0.31-0.63)	30	0.63 (0.43-0.83)
Ever consumed burnt Chicken/duck/goose/pork skin (Yes/No)		140	K	0.22 (0.04-0.40) ⸸	70	0.28 (0.10-0.46)	70	0.35 (0.18-0.52)	110	0.26 (0.12-0.40)	30	0.49 (0.23-0.74)
Red meat	freq	139	ICC	0.48 (0.35-0.60)	70	0.57 (0.39-0.71)	69	0.40 (0.18-0.58)	109	0.52 (0.36-0.64)	30	0.35 (0.00-0.62) ⸸
	port	138	ICC	0.39 (0.23-0.52)	68	0.39 (0.17-0.57)	70	0.36 (0.14-0.55)	108	0.36 (0.18-0.51)	30	0.44 (0.10-0.69)
Poultry	freq	139	ICC	0.55 (0.42-0.66)	70	0.52 (0.32-0.67)	69	0.59 (0.41-0.72)	109	0.52 (0.37-0.65)	30	0.62 (0.34-0.80)
	port	131	ICC	0.44 (0.29-0.57)	65	0.53 (0.32-0.68)	66	0.34 (0.11-0.54)	102	0.40 (0.22-0.55)	29	0.50 (0.16-0.73)
Animal liver	freq	139	ICC	0.57 (0.44-0.67)	70	0.56 (0.37-0.70)	69	0.57 (0.39-0.71)	109	0.57 (0.43-0.69)	30	0.50 (0.19-0.73)
	port	102	ICC	0.46 (0.29-0.60)	51	0.42 (0.16-0.62)	51	0.48 (0.23-0.66)	86	0.46 (0.28-0.61)	16	0.44 (-0.04, 0.76)
Oily fish	freq	139	ICC	0.60 (0.48-0.70)	70	0.67 (0.52-0.78)	69	0.50 (0.30-0.65)	109	0.54 (0.39-0.66)	30	0.76 (0.55-0.88)
	port	79	ICC	0.53 (0.36-0.67)	33	0.68 (0.44-0.83)	46	0.42 (0.16-0.63)	65	0.52 (0.32-0.68)	14	0.60 (0.09-0.85) ⸸
Non-oily fish	freq	139	ICC	0.56 (0.43-0.66)	70	0.58 (0.39-0.72)	69	0.54 (0.35-0.69)	109	0.58 (0.45-0.70)	30	0.48 (0.16-0.71)
	port	115	ICC	0.26 (0.08-0.42) ⸸	53	0.31 (0.05-0.53) ⸸	62	0.23 (-0.02, 0.45)	90	0.33 (0.13-0.50)	25	-0.02 (-0.39, 0.36)
Shellfish	freq	139	ICC	0.61 (0.49-0.70)	70	0.45 (0.24-0.62)	69	0.71 (0.58-0.81)	109	0.54 (0.39-0.66)	30	0.75 (0.55-0.87)
	port	114	ICC	0.40 (0.23-0.54)	56	0.46 (0.22-0.64)	58	0.36 (0.11-0.56)	93	0.39 (0.20-0.55)	21	0.49 (0.08-0.76) ⸸
Leafy green vegetable	freq	138	ICC	0.44 (0.29-0.56)	69	0.44 (0.23-0.61)	69	0.44 (0.22-0.61)	108	0.43 (0.27-0.58)	30	0.33 (-0.02, 0.61)
	port	137	ICC	0.44 (0.29-0.57)	68	0.46 (0.25-0.63)	69	0.42 (0.21-0.60)	107	0.37 (0.20-0.52)	30	0.60 (0.30-0.79)
Other vegetable	freq	140	ICC	0.38 (0.22-0.51)	70	0.51 (0.31-0.66)	70	0.22 (-0.02, 0.43)	110	0.39 (0.22-0.54)	30	0.30 (-0.3, 0.59)
	port	129	ICC	0.60 (0.47-0.70)	63	0.52 (0.32-0.68)	66	0.68 (0.53-0.79)	100	0.60 (0.45-0.71)	29	0.61 (0.32-0.80)

Carrot	freq	140	ICC	0.64 (0.53-0.72)	70	0.58 (0.40-0.72)	70	0.69 (0.55-0.80)	110	0.63 (0.50-0.73)	30	0.66 (0.39-0.82)
	port	108	ICC	0.41 (0.24-0.55)	51	0.46 (0.21-0.65)	57	0.35 (0.10-0.56)	87	0.40 (0.21-0.56)	21	0.44 (0.04-0.73) ┼
Tomato	freq	139	ICC	0.53 (0.40-0.64)	69	0.59 (0.41-0.72)	70	0.50 (0.30-0.66)	109	0.61 (0.47-0.71)	30	0.38 (0.04-0.65) ┼
	port	124	ICC	0.42 (0.26-0.55)	62	0.58 (0.39-0.73)	62	0.19 (-0.06, 0.42)	99	0.46 (0.29-0.60)	25	0.22 (-0.16, 0.55)
Citrus fruit	freq	139	ICC	0.59 (0.47-0.69)	70	0.58 (0.40-0.72)	69	0.60 (0.42-0.73)	109	0.66 (0.54-0.75)	30	0.39 (0.04-0.65) ┼
	port	127	ICC	0.48 (0.33-0.60)	65	0.43 (0.22-0.61)	62	0.53 (0.33-0.69)	99	0.44 (0.26-0.58)	28	0.61 (0.32-0.80)
Other fruits	freq	140	ICC	0.55 (0.42-0.65)	70	0.59 (0.41-0.72)	70	0.50 (0.31-0.66)	110	0.48 (0.32-0.61)	30	0.68 (0.44-0.84)
	port	130	ICC	0.38 (0.22-0.52)	64	0.47 (0.26-0.64)	66	0.27 (0.04-0.48)┼	101	0.39 (0.21-0.55)	29	0.34 (-0.01, 0.62)
Fresh milk	freq	139	ICC	0.74 (0.65-0.80)	70	0.61 (0.44-0.74)	69	0.81 (0.71-0.88)	109	0.72 (0.62-0.80)	30	0.76 (0.55-0.88)
	port	79	ICC	0.53 (0.35-0.67)	38	0.46 (0.16-0.68)	41	0.62 (0.40-0.78)	65	0.58 (0.39-0.72)	14	0.33 (-0.21, 0.72)
Powdered milk	freq	139	ICC	0.44 (0.30-0.57)	70	0.61 (0.44-0.74)	69	0.31 (0.08-0.51)┼	109	0.50 (0.35-0.63)	30	0.14 (-0.24, 0.47)
	port	30	ICC	0.35 (0.01-0.63)┼	17	0.18 (-0.35, 0.60)	13	0.57 (0.09-0.84)┼	25	0.40 (0.05-0.68)┼	5	0.33 (-0.35, 0.89)
Dairy products	freq	140	ICC	0.64 (0.53-0.73)	70	0.61 (0.44-0.74)	70	0.67 (0.52-0.78)	110	0.66 (0.54-0.75)	30	0.57 (0.27-0.77)
	port	106	ICC	0.41 (0.24-0.55)	51	0.43 (0.18-0.63)	55	0.39 (0.14-0.59)	84	0.48 (0.30-0.63)	22	0.15 (-0.17, 0.48)
Eggs	freq	140	ICC	0.57 (0.45-0.67)	70	0.57 (0.39-0.71)	70	0.58 (0.40-0.72)	110	0.53 (0.36-0.65)	30	0.66 (0.40-0.82)
	port	132	ICC	0.29 (0.12-0.44)	66	0.19 (-0.06, 0.41)	66	0.34 (0.11-0.54)	106	0.31 (0.13-0.47)	26	0.18 (-0.22, 0.52)
Tofu	freq	140	ICC	0.59 (0.47-0.69)	70	0.74 (0.61-0.83)	70	0.48 (0.28-0.64)	110	0.55 (0.40-0.67)	30	0.73 (0.51-0.86)
	port	126	ICC	0.45 (0.30-0.58)	64	0.41 (0.18-0.59)	62	0.50 (0.30-0.67)	99	0.44 (0.26-0.58)	27	0.52 (0.18-0.75)
Soybean milk	freq	137	ICC	0.63 (0.51-0.72)	68	0.57 (0.39-0.71)	69	0.68 (0.53-0.79)	108	0.65 (0.52-0.74)	29	0.58 (0.27-0.78)
	port	96	ICC	0.44 (0.27-0.59)	49	0.71 (0.54-0.82)	47	0.19 (-0.08, 0.44)	77	0.51 (0.33-0.66)	19	0.21 (-0.21, 0.58)
Bean curd	freq	139	ICC	0.68 (0.58-0.76)	70	0.61 (0.43-0.74)	69	0.75 (0.63-0.84)	109	0.66 (0.55-0.76)	30	0.72 (0.49-0.86)
	port	112	ICC	0.36 (0.19-0.51)	54	0.35 (0.09-0.56)┼	58	0.39 (0.15-0.59)	89	0.41 (0.23-0.57)	23	0.22 (-0.19, 0.57)
Types of salted fish		139	K	0.31 (0.21-0.41)	69	0.26 (0.12-0.40)	70	0.35 (0.21-0.49)	110	0.30 (0.19-0.41)	29	0.35 (0.13-0.57)
Mouldy salted fish	freq	26	ICC	0.85 (0.68-0.93)	17	0.82 (0.58-0.93)	9	0.82 (0.42-0.96)	21	0.89 (0.74-0.95)	5	0.46 (-0.19, 0.91)
	port	26	ICC	0.69 (0.43-0.85)	17	0.67 (0.31-0.87)	9	0.64 (0.04-0.90)┼	21	0.77 (0.52-0.90)	5	0.00 (-0.51, 0.76)
Firmed salted fish	freq	36	ICC	0.64 (0.40-0.80)	21	0.62 (0.27-0.83)	15	0.64 (0.22-0.86)	26	0.70 (0.45-0.86)	10	0.20 (-0.55, 0.73)
	port	34	ICC	0.56 (0.28-0.76)	20	0.52 (0.11-0.78)	14	0.65 (0.23-0.87)	25	0.52 (0.16-0.76)	9	0.68 (0.07-0.92)┼
Other types of salted fish	freq	0	ICC	-	0	-	0	-	0	-	0	-

	port	0	ICC	-	0	-	0	-	0	-	0	-
Any types of salted fish	freq	13	ICC	0.94 (0.81-0.98)	5	0.73 (-0.01, 0.97)	8	0.97 (0.89-0.99)	11	0.96 (0.85-0.99)	2	0.00 (-1.00, 1.00)
	port	13	ICC	0.38 (-0.22, 0.76)	5	0.60 (-0.17, 0.95)	8	0.36 (-0.28, 0.82)	11	0.77 (0.33-0.93)	2	-4.00 (-9.00, 0.97)
Preserved seafood	freq	139	ICC	0.57 (0.45-0.67)	70	0.48 (0.27-0.64)	69	0.67 (0.52-0.78)	109	0.56 (0.42-0.68)	30	0.58 (0.29-0.78)
	port	112	ICC	0.45 (0.29-0.59)	55	0.35 (0.10-0.57)	57	0.54 (0.33-0.70)	90	0.46 (0.28-0.61)	22	0.46 (0.06-0.73)†
Preserved vegetable	freq	139	ICC	0.59 (0.47-0.69)	70	0.47 (0.27-0.63)	69	0.74 (0.61-0.83)	109	0.49 (0.34-0.62)	30	0.79 (0.60-0.89)
	port	114	ICC	0.47 (0.31-0.60)	55	0.44 (0.20-0.63)	59	0.50 (0.28-0.67)	88	0.42 (0.24-0.58)	26	0.57 (0.26-0.78)
Preserved fruit	freq	140	ICC	0.66 (0.55-0.74)	70	0.58 (0.40-0.72)	70	0.73 (0.60-0.83)	110	0.66 (0.54-0.75)	30	0.64 (0.37-0.81)
	port	90	ICC	0.38 (0.19-0.55)	45	0.23 (-0.07, 0.49)	45	0.52 (0.27-0.70)	73	0.31 (0.09-0.50)†	17	0.58 (0.14-0.83)
Preserved egg	freq	139	ICC	0.62 (0.51-0.72)	70	0.64 (0.48-0.76)	69	0.61 (0.44-0.74)	109	0.60 (0.47-0.71)	30	0.68 (0.43-0.83)
	port	112	ICC	0.48 (0.33-0.61)	53	0.47 (0.23-0.66)	59	0.50 (0.27-0.67)	91	0.46 (0.28-0.60)	21	0.63 (0.28-0.83)
Preserved meat	freq	139	ICC	0.50 (0.37-0.62)	69	0.49 (0.29-0.65)	70	0.52 (0.32-0.67)	109	0.35 (0.17-0.50)	30	0.77 (0.57-0.88)
	port	113	ICC	0.49 (0.34-0.62)	53	0.60 (0.40-0.75)	60	0.36 (0.12-0.56)	94	0.49 (0.32-0.63)	19	0.51 (0.11-0.77)
Processed meat	freq	140	ICC	0.65 (0.55-0.74)	70	0.69 (0.54-0.80)	70	0.61 (0.44-0.74)	110	0.69 (0.58-0.78)	30	0.44 (0.11-0.69)
	port	100	ICC	0.42 (0.24-0.57)	48	0.45 (0.19-0.65)	52	0.39 (0.13-0.60)	84	0.44 (0.25-0.60)	16	0.35 (-0.19-0.72)
Condiments	freq	139	ICC	0.40 (0.25-0.53)	70	0.40 (0.18-0.57)	69	0.41 (0.19-0.59)	109	0.43 (0.26-0.57)	30	0.32 (-0.05, 0.61)
	port	125	ICC	0.28 (0.11-0.43)	63	0.25 (0.01-0.47)†	62	0.31 (0.06-0.52)†	99	0.34 (0.16-0.50)	26	0.22 (-0.10, 0.53)
Green/white tea	freq	140	ICC	0.49 (0.36-0.61)	70	0.38 (0.16-0.57)	70	0.62 (0.45-0.74)	110	0.39 (0.22-0.54)	30	0.70 (0.45-0.84)
	port	77	ICC	0.34 (0.13-0.52)	36	0.47 (0.18-0.69)	41	0.22 (-0.09, 0.50)	63	0.25 (0.01-0.47)†	14	0.59 (0.11-0.85)
Oolong tea	freq	140	ICC	0.64 (0.53-0.73)	70	0.69 (0.55-0.80)	70	0.57 (0.38-0.71)	110	0.51 (0.36-0.64)	30	0.87 (0.75-0.94)
	port	70	ICC	0.08 (-0.15, 0.30)	35	0.29 (-0.03, 0.56)	35	-0.10 (-0.41, 0.24)	57	0.08 (-0.17, 0.32)	13	-0.03 (-0.62, 0.53)
Red/black tea	freq	140	ICC	0.63 (0.52-0.72)	70	0.62 (0.46-0.75)	70	0.64 (0.47-0.76)	110	0.71 (0.60-0.79)	30	0.35 (0.00-0.63)†
	port	88	ICC	0.28 (0.08-0.47)†	45	0.26 (-0.04, 0.51)	43	0.30 (0.01-0.55)†	76	0.27 (0.05-0.47)†	12	0.35 (-0.26, 0.76)
Cantonese-style milk tea	freq	140	ICC	0.70 (0.60-0.77)	70	0.67 (0.51-0.78)	70	0.73 (0.60-0.83)	110	0.66 (0.54-0.75)	30	0.79 (0.60-0.89)
	port	70	ICC	0.23 (-0.01, 0.44)	33	0.25 (-0.10, 0.55)	37	0.22 (-0.12, 0.51)	62	0.29 (0.05-0.50)†	8	0.00 (-0.52, 0.63)
Coffee	freq	139	ICC	0.67 (0.57-0.75)	70	0.62 (0.46-0.75)	69	0.71 (0.57-0.81)	109	0.67 (0.55-0.76)	30	0.67 (0.42-0.83)
	port	44	ICC	0.39 (0.11-0.62)	19	0.42 (-0.05, 0.73)	25	0.35 (-0.06, 0.65)	38	0.44 (0.14-0.67)	6	0.00 (-1.01, 0.79)
Chinese herbal tea	freq	139	ICC	0.63 (0.52-0.72)	70	0.64 (0.47-0.76)	69	0.63 (0.46-0.75)	109	0.64 (0.52-0.74)	30	0.58 (0.28-0.78)

port	109	ICC	0.37 (0.20-0.52)	56	0.27 (0.01-0.50)+	53	0.49 (0.26-0.67)	86	0.41 (0.22-0.57)	23	0.06 (-0.31, 0.43)
**FFQ at age 19-30 years**										
Any changes to your diet during 19-30 years old, compared with 13-18's (Yes/No)	135	K	0.40 (0.24-0.56)	67	0.49 (0.27-0.71)	68	0.30 (0.05-0.54)+	105	0.44 (0.25-0.62)	30	0.30 (-0.04, 0.63)
If yes, which food items										
All meat (yes/no)	77	K	0.22 (0.15-0.28)	39	0.23 (0.14-0.31)	38	0.19 (0.09-0.30)+	61	0.24 (0.17-0.32)	16	0.12 (-0.03, 0.27)
Animal liver (yes/no)	44	K	0.15 (0.07-0.23) +	20	0.04 (-0.04, 0.12)	24	0.19 (0.08-0.30)+	33	0.17 (0.08-0.26)+	11	0.11 (-0.02, 0.24)
Seafood (yes/no)	69	K	0.19 (0.12-0.26)	31	0.18 (0.08-0.27)+	38	0.17 (0.07-0.28)+	55	0.22 (0.14-0.30)	14	0.07 (-0.08, 0.22)
Vegetable (yes/no)	63	K	0.16 (0.09-0.23) +	28	0.17 (0.07-0.26)+	35	0.13 (0.02-0.24)+	48	0.18 (0.10-0.26)	15	0.10 (-0.05, 0.25)
Fruit (yes/no)	72	K	0.18 (0.11-0.25)	30	0.19 (0.10-0.29)	42	0.14 (0.03-0.24)+	53	0.20 (0.12-0.27)	19	0.14 (0.01-0.27)+
Dairy products (yes/no)	52	K	0.14 (0.06-0.21) +	25	0.14 (0.04-0.25)+	27	0.11 (0.01-0.22)+	37	0.15 (0.06-0.23)+	15	0.11 (-0.03, 0.25)
Egg (yes/no)	49	K	0.18 (0.10-0.25)	24	0.16 (0.05-0.26)+	25	0.18 (0.07-0.28)+	35	0.19 (0.10-0.28)	14	0.13 (-0.02, 0.28)
Soybean products (yes/no)	55	K	0.16 (0.08-0.23) +	29	0.15 (0.05-0.25)+	26	0.15 (0.04-0.27)+	38	0.18 (0.09-0.26)+	17	0.10 (-0.05, 0.24)
Salted fish (yes/no)	49	K	0.18 (0.11-0.26)	25	0.19 (0.08-0.29)+	24	0.17 (0.08-0.27)+	37	0.20 (0.11-0.29)	12	0.14 (0.01-0.28)+
Preserved food (yes/no)	50	K	0.17 (0.09-0.24) +	27	0.18 (0.08-0.28)+	23	0.15 (0.04-0.26)+	35	0.19 (0.10-0.28)	15	0.11 (-0.03, 0.25)
Beverage	69	K	0.18 (0.11-0.25)	32	0.19 (0.10-0.28)	37	0.15 (0.04-0.26)+	54	0.22 (0.14-0.29)	15	0.07 (-0.06, 0.19)
**FFQ 10 years ago**										
Any changes to your diet before 10 years ago, compared with 19-30's	140	K	0.59 (0.47-0.70)	70	0.57 (0.41-0.73)	70	0.60 (0.44-0.76)	110	0.56 (0.43-0.69)	30	0.69 (0.46-0.91)
If yes, which food items										
All meat (yes/no)	97	K	0.27 (0.13-0.42)	49	0.24 (0.03-0.44)+	48	0.28 (0.08-0.48)+	77	0.24 (0.07-0.40)+	20	0.40 (0.08-0.72)+
Animal liver (yes/no)	98	K	0.26 (0.11-0.41)	49	0.28 (0.08-0.49)+	49	0.21 (0.01-0.42)+	77	0.26 (0.09-0.43)+	21	0.27 (-0.05, 0.59)
Seafood (yes/no)	95	K	0.21 (0.06-0.36) +	49	0.15 (-0.04, 0.35)	46	0.25 (0.04-0.45)+	75	0.17 (0.05-0.34)+	20	0.32 (-0.00, 0.65)
Vegetable (yes/no)	97	K	0.23 (0.08-0.37) +	49	0.15 (-0.04, 0.35)	48	0.26 (0.05-0.48)+	77	0.19 (0.02-0.35)+	20	0.38 (0.06-0.70)+
Fruit (yes/no)	98	K	0.22 (0.08-0.37) +	49	0.24 (0.04-0.44)+	49	0.18 (-0.02, 0.38)	77	0.19 (0.03-0.36)+	21	0.32 (0.00-0.63)+
Dairy products (yes/no)	96	K	0.29 (0.13-0.44)	49	0.23 (0.02-0.45)+	47	0.31 (0.11-0.52)	75	0.20 (0.03-0.38)+	21	0.56 (0.27-0.85)

Egg (yes/no)	96	K	0.15 (0.00-0.30) +	49	0.21 (0.00-0.42)+	47	0.06 (-0.16, 0.27)	77	0.08 (-0.08, 0.25)	19	0.43 (0.10-0.77)
Soybean products (yes/no)	97	K	0.23 (0.08-0.39) +	49	0.21 (0.00-0.43)+	48	0.22 (0.01-0.44)+	77	0.17 (-0.01, 0.34)	20	0.48 (0.18-0.77)
Salted fish (yes/no)	94	K	0.23 (0.08-0.39) +	48	0.26 (0.04-0.47)+	46	0.19 (-0.02, 0.40)	73	0.25 (0.07-0.42)+	21	0.20 (-0.12, 0.53)
Preserved food (yes/no)	97	K	0.22 (0.07-0.37) +	49	0.22 (0.01-0.43)+	48	0.18 (-0.03, 0.39)	76	0.24 (0.07-0.41)+	21	0.14 (-0.18, 0.46)
Beverage	97	K	0.21 (0.06-0.36) +	49	0.27 (0.06-0.47)+	48	0.13 (-0.08, 0.33)	77	0.16 (-0.01, 0.33)	20	0.40 (0.07-0.72) +

* only subjects who filled out the question in both questionnaires were included in the analysis.

ICC: intra-class correlation coefficient. K: Cohen's kappa. Kw: Weighted kappa. Weighted kappa. Freq: frequency (Never/Less than once a month/Once a month/1-3 per week/4-6 per week/1-2 per day/3+ per day). Differences (difference between coefficients >0.30, and were tested by a Fisher Z transformation) were shade. (Coefficients: 0-0.20 [poor]; 0.20-0.40 [fair]; 0.40-0.60 [moderate]; 0.60-0.80 [substantial]; 0.80-1.00 [almost perfect]). The format of 95% cell: (x1-x2) indicating p<0.01, unless otherwise stated (+: indicating p between 0.05 and0.01); the other format: (-x1, x2) indicating p-value>0.05.

Supplementary Table s3. Questionnaire items and reliability coefficients with their 95% confidence intervals by time between questionnaires and education

Questionnaire item	N*	Method	Time between questionnaires				Education			
			2-29 weeks, (N=70)		≥30 weeks, (N=70)		Secondary or less (N=92)		Postsecondary (N=47)	
			n		n		n		n	
Siblings										
No. of older brother	70	ICC	34	0.39 (0.06-0.64)	36	0.77 (0.60-0.88)	52	0.50 (0.27-0.68)	18	1.00
No. of younger brother	68	ICC	39	0.99 (0.98-0.99)	29	1.00	49	0.99 (0.99-1.00)	19	1.00
No. of older sister	70	ICC	31	1.00	39	0.74 (0.55-0.85)	49	0.76 (0.62-0.86)	21	1.00
No. of younger sister	64	ICC	34	0.99 (0.97-0.99)	30	0.21 (-0.14, 0.52)	47	0.76 (0.61-0.86)	17	0.31 (-0.17, 0.67)
Marital status (single/married/divorced/widowed)	140	K	70	0.84 (0.71-0.97)	70	0.95 (0.88-1.00)	92	0.89 (0.79-0.98)	47	0.92 (0.81-1.00)
Income										
Employment status (self-employed/employed/retired/housewives/student/unemployed)	138	K	69	0.68 (0.54-0.82)	69	0.62 (0.45-0.78)	90	0.64 (0.51-0.77)	47	0.70 (0.47-0.92)
Personal income (none/less than 15k/15-24.9k/25-39.9k/40k or above)	138	Kw	70	0.68 (0.45-0.90)	68	0.80 (0.68-0.92)	90	0.53 (0.32-0.73)	47	0.82 (0.62-1.00)
Household income (none/less than 15k/15-24.9k/25-39.9k/40k or above)	133	Kw	67	0.47 (0.22-0.73)	66	0.75 (0.62-0.87)	86	0.46 (0.24-0.68)	47	0.69 (0.47-0.92)
Off-springs										
No. of son	62	ICC	29	0.99 (0.97-0.99)	33	1.00	44	0.99 (0.98-0.99)	17	1.00
No. of daughter	59	ICC	29	1.00	30	1.00	42	1.00	16	1.00
None	45	K	24	0.94 (0.85-1.00)	21	0.97 (0.90-1.00)	6	0.95 (0.87-1.00)	14	0.96 (0.87-1.00)

Birth place (HK/Macau/Guangxi/Guangdong /Fujian/Hunan/Others/unknown)	140	K	70	0.94 (0.85-1.00)	70	0.94 (0.86-1.00)	92	0.91 (0.83-1.00)	47	1.00
Mother's birth place (HK/Macau/Guangxi/Guangdong /Fujian/Hunan/Others/unknown)	140	K	70	0.74 (0.61-0.88)	70	0.76 (0.64-0.89)	92	0.77 (0.66-0.88)	47	0.71 (0.53-0.89)
Housing type at 10 years old (Temporary/Public/Home Ownership Scheme/Private (owner)/Private (rent)/Boat/Shanty/Stone/Village /Others)	140	K	70	0.85 (0.75-0.94)	70	0.70 (0.57-0.82)	92	0.71 (0.61-0.82)	47	0.89 (0.78-0.99)
Education (None/Old-style school/Primary/Secondary 1-3/Secondary 4-5/Matriculation F.6-7/Technical)										
Subject's	140	K	70	0.89 (0.81-0.97)	70	0.85 (0.75-0.95)	92	0.85 (0.76-0.94)	47	0.84 (0.70-0.98)
Subject's father	140	K	70	0.60 (0.47-0.73)	70	0.50 (0.36-0.64)	92	0.54 (0.42-0.66)	47	0.56 (0.39-0.72)
Subject's mother	140	K	70	0.74 (0.62-0.85)	70	0.61 (0.48-0.75)	92	0.61 (0.49-0.73)	47	0.74 (0.60-0.88)
Cancer history (Yes/No)										
Men	140	K	70	0.85 (0.71-0.99)	70	0.85 (0.72-0.99)	92	0.87 (0.76-0.98)	47	0.79 (0.56-1.00)
Women	140	K	70	1.00	70	0.96 (0.88-1.00)	92	0.97 (0.92-1.00)	47	1.00
Mother experienced illness (Yes/No)										
During pregnant	140	K	70	0.55 (0.36-0.74)	70	0.38 (0.20-0.55)	92	0.45 (0.29-0.62)	47	0.46 (0.22-0.69)
During delivery	140	K	70	0.40 (0.16-0.64)	70	0.37 (0.16-0.59)	92	0.43 (0.22-0.63)	47	0.30 (0.03-0.56) ┼

Birth delivery mode (Natural birth/Caesarean section/Unknown)	140	K	70	0.48 (0.20-0.75)	70	0.31 (-0.02, 0.64)	92	0.31 (0.02-0.59) †	47	0.48 (0.16-0.80)
Single birth or multiple births	139	K	70	0.49 (-0.11, 1.00)	69	-0.01 (-0.02, 0.01)	91	0.32 (-0.17, 0.81)	47	-
Birth period (Premature/Full-term/Post-term)	140	K	70	0.58 (0.39-0.77)	70	0.41 (0.21-0.60)	92	0.65 (0.49-0.82)	47	0.72 (0.49-0.94)
Birth weight (kg)	140	ICC	70	0.69 (0.55-0.80)	70	0.49 (0.29-0.65)	92	0.55 (0.39-0.68)	47	0.66 (0.47-0.80)
Breastfed (Yes/No/Unknown)	140	K	70	0.41 (0.19-0.62)	70	0.37 (0.11-0.64)	92	0.57 (0.44-0.71)	47	0.61 (0.45-0.77)
Breastfed period (months)	140	ICC	70	0.53 (0.34-0.68)	70	0.39 (0.17-0.57)	92	0.42 (0.23-0.57)	47	0.53 (0.29-0.70)
Family history of cancer (Yes/No)	136	K	68	0.88 (0.76-0.99)	68	0.85 (0.73-0.98)	90	0.89 (0.79-0.98)	46	0.83 (0.66-0.99)
Family history of nasopharyngeal carcinoma (Yes/No)	75	K	31	1.00	44	1.00	51	1.00	24	1.00
Omega-3										
Ever/never	140	K	70	0.57 (0.37-0.76)	70	0.60 (0.42-0.78)	92	0.52 (0.34-0.70)	47	0.66 (0.44-0.87)
Age started, years	44	ICC	23	0.58 (0.25-0.80)	21	0.63 (0.30-0.83)	24	0.40 (-0.01, 0.69)	20	0.91 (0.79-0.96)
Duration, years	43	ICC	23	0.50 (0.14-0.75)	20	0.68 (0.35-0.86)	24	0.42 (0.04-0.70)†	19	0.88 (0.71-0.95)
Frequency (Less than once a month/Once a month/1-3 per week/4-6 per week/1-2 per day/3+ per day)	36	ICC	20	0.81 (0.58-0.92)	16	0.32 (-0.18, 0.69)	22	0.61 (0.27-0.82)	14	0.59 (0.14-0.84)
Vitamins										
Ever/never	140	K	70	0.49 (0.27-0.70)	70	0.36 (0.14-0.58)	92	0.33 (0.12-0.53)	47	0.53 (0.30-0.76)
Multi-vitamins										
Ever/never	140	K	70	0.57 (0.29-0.86)	70	0.51 (0.14-0.88)	92	0.37 (-0.03, 0.76)	47	0.62 (0.34-0.90)
Age started, years	9	ICC	6	0.61 (-0.11, 0.93)	3	0.11 (-0.58, 0.95)	2	-	7	0.66 (0.02-0.93) †
Duration, years	9	ICC	6	0.99 (0.99-1.00)	3	0.99 (0.35-1.00)	2	0.99 (0.94-1.00)	7	0.99 (0.98-1.00)

Frequency (Less than once a month/Once a month/1-3 per week/4-6 per week/1-2 per day/3+ per day)	9	ICC	6	0.52 (-0.32, 0.91)	3	0.92 (0.22-1.00)	2	-	7	0.53 (-0.32, 0.90)
Vitamins A										
Ever/never	140	K	70	-0.02 (-0.05, 0.01)	70	-	92	-	47	-
Age started, years	0	ICC	0	-	0	-	0	-	0	-
Duration, years	0	ICC	0	-	0	-	0	-	0	-
Frequency (Less than once a month/Once a month/1-3 per week/4-6 per week/1-2 per day/3+ per day)	0	ICC	0	-	0	-	0	-	0	-
Vitamins B										
Ever/never	140	K	70	0.52 (0.16-0.87)	70	0.45 (0.09-0.82) +	92	0.47 (0.11-0.82)	47	0.49 (0.10-0.87)
Age started, years	6	ICC	3	0.35 (-1.10, 0.98)	3	0.63 (-0.36, 0.99)	3	0.04 (-0.22, 0.90)	3	0.53 (-2.35, 0.99)
Duration, years	6	ICC	3	0.74 (-1.62, 0.99)	3	0.99 (0.41-1.00)	3	0.29 (-1.00, 0.97)	3	0.73 (-0.33, 0.99)
Frequency (Less than once a month/Once a month/1-3 per week/4-6 per week/1-2 per day/3+ per day)	5	ICC	2	-	3	0.27 (-0.85, 0.97)	3	-0.01 (-0.95, 0.95)	2	1.00
Vitamins C										
Ever/never	140	K	70	0.28 (-0.08, 0.63)	70	0.36 (0.07-0.64) +	92	0.15 (-0.12, 0.41)	47	0.55 (0.23-0.87)
Age started, years	7	ICC	2	0.90 (-0.02, 1.00)	5	0.48 (-0.32, 0.93)	2	0.59 (-0.39, 0.99)	5	0.83 (0.19-0.98)
Duration, years	7	ICC	2	-	5	0.99 (0.98-1.00)	2	-0.17 (-1.08, 0.99)	5	0.99 (0.98-1.00)
Frequency (Less than once a month/Once a month/1-3 per week/4-6 per week/1-2 per day/3+ per day)	7	ICC	2	0.50 (0.01-0.99) +	5	0.94 (0.64-0.99)	2	0.00 (-0.99, 0.99)	5	0.91 (0.43-0.99)

Vitamins D											
Ever/never consumed	140	K		70	0.10 (0.16-0.36)	70	0.26 (-0.19, 0.70)	92	-0.04 (-0.09, 0.01)	47	0.22 (-0.19, 0.62)
Age started, years	1	ICC	-	1	-	0	-	1	-	0	-
Duration, years	1	ICC	-	1	-	0	-	1	-	0	-
Frequency (Less than once a month/Once a month/1-3 per week/4-6 per week/1-2 per day/3+ per day)	1	ICC	-	1	-	0	-	1	-	0	-
Vitamins E											
Ever/never	140	K		70	0.79 (0.50-1.00)	70	0.30 (-0.20, 0.80)	92	0.75 (0.48-1.00)	47	-0.03 (-0.07, 0.01)
Age started, years	5	ICC		4	0.35 (-0.33, 0.93)	1	-	5	0.50 (-0.20, 0.92)	0	-
Duration, years	5	ICC		4	0.96 (0.59-1.00)	1	-	5	0.92 (0.37-0.99)	0	-
Frequency (Less than once a month/Once a month/1-3 per week/4-6 per week/1-2 per day/3+ per day)	5	ICC		4	0.83 (-0.43, 0.99)	1	-	5	0.86 (0.08-0.99) †	0	-
Oral health											
No. of dental caries	138	Kw		70	0.59 (0.39-0.80)	68	0.55 (0.36-0.73)	91	0.52 (0.35-0.70)	46	0.67 (0.47-0.88)
No. of teeth extracted due to dental caries	92	Kw		48	0.80 (0.67-0.92)	44	0.50 (0.22-0.79)	60	0.52 (0.28-0.77)	31	0.82 (0.66-0.98)
Periodontal disease (yes/no)	138	K		69	0.84 (0.71-0.98)	69	0.72 (0.55-0.89)	90	0.74 (0.60-0.89)	47	0.85 (0.69-1.00)
No. of teeth extracted due to periodontal disease	42	Kw		21	0.77 (0.46-1.00)	21	0.68 (0.39-0.97)	29	0.64 (0.35-0.92)	13	0.92 (0.82-1.00)
Active smoking											
Ever/never	139	K		70	0.97 (0.91-1.00)	69	0.86 (0.73-0.99)	91	0.91 (0.81-1.00)	47	0.95 (0.86-1.00)

Time between wake up and first cig (Less than 5/6-30/31-60/60+ minutes)	140	Kw	70	0.80 (0.64-0.95)	70	0.80 (0.63-0.97)	92	0.77 (0.62-0.92)	47	0.86 (0.73-0.99)
Age starting years	46	ICC	27	0.78 (0.57-0.89)	19	0.85 (0.66-0.94)	31	0.77 (0.59-0.88)	15	0.92 (0.78-0.97)
Smoking amount										
Before age of 18 years	24	ICC	14	0.41 (-0.16, 0.76)	10	0.90 (0.65-0.97)	17	0.49 (0.04-0.78) †	7	0.83 (0.27-0.97)
After age of 18 years	46	ICC	27	0.63 (0.33-0.81)	19	0.90 (0.76-0.96)	31	0.59 (0.30-0.78)	15	0.88 (0.68-0.96)
Age at quitting	32	ICC	19	0.99 (0.99-1.00)	13	0.99 (0.97-1.00)	23	0.99 (0.98-1.00)	9	0.98 (0.93-1.00)
Duration of quitting	24	ICC	15	0.78 (0.48-0.92)	9	0.99 (0.98-1.00)	18	0.84 (0.64-0.94)	6	0.83 (0.18-0.98)
Passive smoking (*household*)										
**childhood**										
No. of co-living smokers	138	ICC	70	0.85 (0.77-0.91)	68	0.67 (0.50-0.79)	90	0.74 (0.63-0.82)	47	0.83 (0.71-0.90)
Co-living smoker 1										
Relationship (Father/Mother/Grandfather/Grandmother/Maternal grandfather/Maternal grandmother/Siblings/Others)	82	K	40	0.52 (0.25-0.80)	42	0.68 (0.43-0.93)	52	0.47 (0.22-0.73)	29	0.79 (0.54-1.00)
Exposed time/day (Less than 5 minutes/30-60 minutes/1-2 hours/2+ hours)	81	Kw	40	0.56 (0.29-0.82)	41	0.54 (0.27-0.80)	51	0.55 (0.31-0.78)	29	0.54 (0.28-0.80)
Years of co-living	77	ICC	38	0.15 (-0.18, 0.45)	39	0.47 (0.19-0.68)	47	0.20 (-0.08, 0.46)	29	0.67 (0.41-0.83)
Co-living smoker 2										
Relationship (Father/Mother/Grandfather/Grandmother/Maternal	23	K	10	0.63 (0.29-0.96)	13	0.58 (0.26-0.90)	17	0.63 (0.35-0.91)	5	0.33 (-0.19, 0.86)

grandfather/Maternal grandmother/Siblings/Others)									
Exposed time/day (Less than 5 minutes/30-60 minutes/1-2 hours/2+ hours)	23	Kw	10	0.21 (-0.47, 0.88)	13	0.67 (0.48-0.86)	17	0.48 (0.05-0.91) ┼	5	0.21 (-0.50, 0.92)
Years of co-living	23	ICC	10	0.88 (0.62-0.97)	13	0.03 (-0.30, 0.47)	17	0.52 (0.08-0.79) ┼	5	0.06 (-0.48, 0.79)
Co-living smoker 3									
Relationship (Father/Mother/Grandfather/Grandmother/Maternal grandfather/Maternal grandmother/Siblings/Others)	8	K	6	1.00	2	-	6	1.00	1	-
Exposed time/day (Less than 5 minutes/30-60 minutes/1-2 hours/2+ hours)	8	Kw	6	-0.13 (-0.68, 0.43)	2	-	6	0.09 (-0.76, 0.93)	1	-
Years of co-living	8	ICC	6	0.99 (0.92-1.00)	2	-	6	1.00	1	-
**adulthood**									
No. of co-living smokers	137	ICC	70	0.70 (0.55-0.80)	67	0.69 (0.55-0.80)	89	0.69 (0.56-0.78)	47	0.66 (0.46-0.79)
Co-living smoker 1									
Relationship (Father/Mother/Grandfather/Grandmother/Maternal grandfather/Maternal grandmother/Siblings/Others)	65	K	31	0.63 (0.35-0.92)	34	0.71 (0.51-0.92)	45	0.71 (0.50-0.91)	19	0.61 (0.29-0.93)
Exposed time/day (Less than 5 minutes/30-60 minutes/1-2 hours/2+ hours)	63	Kw	30	0.47 (0.15-0.79)	33	0.26 (0.04-0.48) ┼	44	0.36 (0.07-0.65) ┼	18	0.37 (-0.06, 0.80)
Years of co-living	64	ICC	30	0.81 (0.64-0.91)	34	0.54 (0.25-0.74)	44	0.64 (0.43-0.79)	19	0.79 (0.54-0.91)

Co-living smoker 2										
Relationship (Father/Mother/Grandfather/Grandmother/Maternal grandfather/Maternal grandmother/Siblings/Others)	21	K	9	0.48 (0.17-0.79)	12	0.65 (0.33-0.98)	17	0.62 (0.35-0.89)	3	0.40 (-0.37, 1.00)
Exposed time/day (Less than 5 minutes/30-60 minutes/1-2 hours/2+ hours)	21	Kw	9	-0.22 (-0.43, -0.01)	12	0.58 (0.24-0.92)	17	0.38 (-0.05, 0.80)	3	-
Years of co-living	21	ICC	9	0.83 (0.41-0.96)	12	0.03 (-0.46, 0.56)	17	0.57 (0.15-0.82)		-0.30 (-0.91, 0.90)
Co-living smoker 3										
Relationship (Father/Mother/Grandfather/Grandmother/Maternal grandfather/Maternal grandmother/Siblings/Others)	4	K	3	-	1	-	2	1.00	1	-
Exposed time/day (Less than 5 minutes/30-60 minutes/1-2 hours/2+ hours)	4	Kw	3	-	1	-	2	-0.43 (-1.00, 0.25)	1	-
Years of co-living	4	ICC	3	-	1	-	2	0.80 (-0.63, 1.00)	1	-
Passive smoking (*workplace*)										
**childhood (before 18 years old)**										
Ever/never	138	K	69	0.32 (0.14-0.50)	69	0.24 (0.06-0.41)	90	0.29 (0.14-0.45)	47	0.22 (0.01-0.43) †
Time of exposed/day (Less than 5 minutes/30-60 minutes/1-2 hours/2+ hours)	23	Kw	14	0.57 (0.12-1.00)	9	0.50 (0.01-0.99)	19	0.49 (0.09-0.88) †	3	0.77 (0.58-0.96)

How long did you work that exposed to passive smoking, years	23	ICC	14	0.48 (-0.07, 0.80)	9	0.60 (-0.06, 0.89)	19	0.43 (-0.03, 0.74)	3	0.94 (0.32-1.00)
**adulthood (after 18 years old)**										
Ever/never	136	K	68	0.49 (0.27-0.70)	68	0.62 (0.43-0.80)	89	0.41 (0.21-0.62)	46	0.70 (0.50-0.90)
Time of exposed/day (Less than 5 minutes/30-60 minutes/1-2 hours/2+ hours)	77	Kw	38	0.55 (0.29-0.81)	39	0.37 (0.09-0.64) †	55	0.31 (0.05-0.58) †	21	0.54 (0.24-0.83)
How long did you work that exposed to passive smoking, years	76	ICC	37	0.62 (0.38-0.79)	39	0.49 (0.21-0.69)	54	0.60 (0.40-0.75)	21	0.33 (-0.13, 0.66)
Occupational hazards										
Exposed/non-exposed	139	K	70	0.73 (0.57-0.88)	69	0.54 (0.34-0.73)	91	0.58 (0.41-0.75)	47	0.70 (0.50-0.90)
Dust	139	K	70	0.77 (0.62-0.91)	69	0.54 (0.34-0.74)	92	0.58 (0.41-0.74)	47	0.80 (0.62-0.98)
Chemical	139	K	70	0.48 (0.23-0.74)	69	0.20 (-0.01, 0.40)	92	0.30 (0.11-0.50)	47	0.35 (0.05-0.65) †
Fumes	139	K	70	0.61 (0.41-0.80)	69	0.45 (0.19-0.70)	92	0.51 (0.32-0.69)	47	0.56 (0.23-0.90)
Acid or alkali	139	K	70	0.61 (0.35-0.87)	69	0.42 (0.01-0.83) †	92	0.54 (0.28-0.80)	47	0.65 (0.20-1.00)
Alcohol drinking										
Ever/never	136	K	69	0.68 (0.51-0.85)	67	0.57 (0.37-0.77)	89	0.69 (0.53-0.84)	46	0.52 (0.27-0.76)
Ever drink once per month	48	K	26	0.69 (0.42-0.97)	22	0.55 (0.20-0.89)	34	0.65 (0.39-0.90)	14	0.55 (0.11-0.99)
Age at starting	53	ICC	31	0.58 (0.29-0.77)	22	0.66 (0.33-0.84)	37	0.54 (0.27-0.73)	16	0.80 (0.52-0.92)
Age at stopping	19	ICC	13	0.99 (0.98-1.00)	6	0.88 (0.22-0.98)	12	0.95 (0.85-0.99)	7	0.97 (0.80-0.99)
Duration of stopping drinking	4	ICC	4	0.99 (0.94-1.00)	0	-	2	0.99 (0.89-1.00)	2	0.99 (0.90-1.00)
Red wine										

Frequency (Less than once a month/Once a month/1-3 per week/4-6 per week/1-2 per day/3+ per day)	23	ICC	12	0.73 (0.30-0.91)	11	0.27 (-0.42, 0.74)	14	0.74 (0.36-0.91)	9	-0.11 (-0.82, 0.59)
Portion, glasses	16	ICC	9	0.25 (-0.56, 0.77)	7	0.64 (-0.11, 0.93)	9	0.38 (-0.33, 0.82)	7	0.64 (-0.07, 0.93)
white wine										
Frequency (Less than once a month/Once a month/1-3 per week/4-6 per week/1-2 per day/3+ per day)	7	ICC	4	0.71 (-0.69, 0.98)	3	0.00 (-0.95, 0.95)	4	0.71 (-0.69, 0.98)	3	-
Portion, glasses	7	ICC	4	0.62 (-0.41, 0.97)	3	0.60 (-0.62, 0.99)	4	0.62 (-0.41, 0.97)	3	0.60 (-0.62, 0.99)
beer										
Frequency (Less than once a month/Once a month/1-3 per week/4-6 per week/1-2 per day/3+ per day)	47	ICC	27	0.79 (0.59-0.90)	20	0.53 (0.14-0.78)	33	0.68 (0.44-0.83)	14	0.75 (0.39-0.91)
Portion (unit: pint)	2	ICC	1	-	1	-	2	-	0	-
Portion (unit: 375ml/can)	8	ICC	5	0.83 (0.18-0.98)	3	0.39 (-0.79, 0.98)	6	0.35 (-0.71, 0.88)	2	-
Portion (unit: 500ml/can)	2	ICC	2	-	0	-	2	-	3	-
Portion (unit: 330ml/bottle)	3	ICC	1	-	2	0.72 (-0.74, 1.00)	2	0.72 (-0.74, 1.00)	1	-
Portion (unit: 600ml/bottle)	6	ICC	3	-0.35 (-0.83, 0.87)	3	0.51 (-0.70, 0.98)	6	0.22 (-0.37, 0.81)	0	-
spirits										
Frequency (Less than once a month/Once a month/1-3 per week/4-6 per week/1-2 per day/3+ per day)	14	ICC	9	0.59 (-0.09, 0.89)	5	0.33 (-0.97, 0.91)	9	0.48 (-0.26, 0.86)	5	0.58 (-0.61, 0.95)

Portion (unit: Chinese-style glass)	2	ICC	1	-	1	-	2	-	0	-
Portion (unit: Western-style glass)	4	ICC	4	0.99 (0.94-1.00)	0	-	3	0.99 (0.83-1.00)	1	-
Sun exposure										
10 years ago										
Outdoor exercise (Less than once a month/Once a month/1-3 per week/4-6 per week/Daily)	140	Kw	70	0.82 (0.73-0.92)	70	0.58 (0.39-0.76)	92	0.63 (0.47-0.79)	47	0.54 (0.74-0.94)
Duration of sun exposure (Less than 1/2-4/5-7/8-10 hours)	140	Kw	70	0.88 (0.81-0.95)	70	0.89 (0.82-0.96)	92	0.85 (0.77-0.93)	47	0.94 (0.90-0.98)
Avoid sun exposure (Never/Ever)	140	K	70	0.56 (0.42-0.70)	70	0.40 (0.26-0.54)	92	0.37 (0.24-0.50)	47	0.67 (0.52-0.83)
Protection from sunshine (Never/Ever)	140	K	70	0.57 (0.44-0.71)	70	0.46 (0.32-0.61)	92	0.47 (0.65-0.88)	47	0.58 (0.42-0.74)
19-30 years ago										
Outdoor exercise (Less than once a month/Once a month/1-3 per week/4-6 per week/Daily)	138	Kw	70	0.44 (0.24-0.63)	68	0.48 (0.27-0.69)	90	0.44 (0.25-0.62)	47	0.45 (0.23-0.68)
Duration of sun exposure (Less than 1/2-4/5-7/8-10 hours)	138	Kw	70	0.43 (0.20-0.67)	68	0.49 (0.26-0.72)	90	0.49 (0.28-0.71)	47	0.31 (0.07-0.54) ‡
Avoid sun exposure (Never/Ever)	139	K	70	0.35 (0.18-0.51)	69	0.19 (0.04-0.35) ‡	91	0.28 (0.14-0.42)	47	0.19 (-0.03, 0.41)

Protection from sunshine (Never/Ever)	139	K	70	0.34 (0.18-0.51)	69	0.17 (0.01-0.33) ┼	91	0.27 (0.13-0.41)	47	0.19 (-0.02, 0.40)
13-18 years ago										
Outdoor exercise (Less than once a month/Once a month/1-3 per week/4-6 per week/Daily)	138	Kw	70	0.50 (0.28-0.71)	68	0.54 (0.33-0.76)	90	0.49 (0.31-0.67)	47	0.57 (0.30-0.84)
Duration of sun exposure (Less than 1/2-4/5-7/8-10 hours)	138	Kw	70	0.52 (0.25-0.78)	68	0.58 (0.39-0.77)	90	0.56 (0.35-0.77)	47	0.47 (0.25-0.69)
Avoid sun exposure (Never/Ever)	139	K	70	0.36 (0.21-0.52)	69	0.30 (0.11-0.49)	91	0.40 (0.25-0.56)	47	0.17 (-0.03, 0.37)
Protection from sunshine (Never/Ever)	139	K	70	0.24 (0.09-0.40) ┼	69	0.24 (0.06-0.41) ┼	91	0.29 (0.14-0.44)	47	0.09 (-0.12, 0.30)
6-12 years ago										
Outdoor exercise (Less than once a month/Once a month/1-3 per week/4-6 per week/Daily)	137	Kw	69	0.59 (0.39-0.79)	68	0.32 (0.08-0.55) ┼	89	0.41 (0.21-0.61)	47	0.56 (0.32-0.79)
Duration of sun exposure (Less than 1/2-4/5-7/8-10 hours)	137	Kw	69	0.33 (0.02-0.64) ┼	68	0.32 (0.10-0.55)	90	0.30 (0.05-0.55)	46	0.33 (0.07-0.60) ┼
Avoid sun exposure (Never/Ever)	139	K	70	0.37 (0.18-0.55)	69	0.18 (0.01-0.34) ┼	91	0.28 (0.13-0.43)	47	0.23 (-0.00, 0.45)
Protection from sunshine (Never/Ever)	139	K	70	0.29 (0.10-0.48)	69	0.30 (0.09-0.52)	91	0.29 (0.11-0.48)	47	0.28 (0.05-0.50) ┼
Skin tone										

Current											
Face		138	Kw	70	0.54 (0.33-0.76)	68	0.59 (0.43-0.75)	90	0.61 (0.47-0.75)	47	0.27 (-0.04, 0.57)
Hand		139	Kw	70	0.36 (0.10-0.63)	69	0.57 (0.36-0.77)	91	0.46 (0.25-0.66)	47	0.40 (0.17-0.62)
10 years ago: hand		91	Kw	46	0.47 (0.26-0.68)	45	0.59 (0.41-0.76)	66	0.53 (0.38-0.69)	24	0.50 (0.21-0.79)
Age 19-30 years: hand		138	Kw	70	0.54 (0.37-0.70)	68	0.46 (0.27-0.65)	90	0.51 (0.35-0.67)	47	0.43 (0.24-0.62)
Age 13-18 years: hand		137	Kw	68	0.52 (0.36-0.68)	69	0.50 (0.34-0.66)	90	0.49 (0.34-0.64)	46	0.56 (0.40-0.73)
Age 6-12 years: hand		132	Kw	66	0.54 (0.35-0.73)	66	0.59 (0.42-0.75)	85	0.54 (0.37-0.70)	46	0.63 (0.45-0.81)
Body figure, height and weight											
**current**											
Body figure: male		105	Kw	55	0.72 (0.59-0.85)	50	0.62 (0.44-0.80)	67	0.63 (0.50-0.77)	37	0.77 (0.61-0.93)
Body figure: female		28	Kw	14	0.70 (0.39-1.00)	14	0.79 (0.65-0.94)	22	0.75 (0.56-0.93)	6	0.88 (0.80-0.97)
Height, cm		130	ICC	66	0.93 (0.89-0.96)	64	0.96 (0.93-0.97)	84	0.95 (0.93-0.97)	46	0.92 (0.86-0.95)
Weight, kg		137	ICC	69	0.94 (0.89-0.97)	68	0.86 (0.60-0.94)	89	0.83 (0.58-0.91)	47	0.96 (0.92-0.98)
**10 years ago**											
Body figure: male		78	Kw	40	0.68 (0.55-0.81)	38	0.58 (0.36-0.80)	54	0.60 (0.44-0.76)	23	0.73 (0.01-0.91) †
Body figure: female		19	Kw	10	0.57 (0.19-0.95)	9	0.79 (0.66-0.92)	15	0.62 (0.38-0.86)	4	0.70 (0.45-0.95)
Weight, kg		96	ICC	49	0.31 (0.03-0.54) †	47	0.36 (0.08-0.58) †	68	0.25 (0.02-0.46) †	27	0.47 (0.12-0.72)
**Age 19-30 years**											
Body figure: male		109	Kw	56	0.61 (0.39-0.83)	53	0.51 (0.30-0.72)	68	0.43 (0.22-0.65)	40	0.73 (0.54-0.92)
Body figure: female		28	Kw	14	0.77 (0.55-0.99)	17	0.73 (0.50-0.95)	22	0.74 (0.56-0.92)	6	0.93 (0.87-0.99)
**Age 13-18 years**											
Body figure: male		110	Kw	56	0.71 (0.52-0.90)	54	0.63 (0.44-0.82)	68	0.51 (0.28-0.73)	41	0.81 (0.70-0.92)
Body figure: female		28	Kw	14	0.66 (0.35-0.97)	14	0.50 (0.10-0.90)	22	0.67 (0.46-0.89)	6	0.53 (0.18-0.87)
**Age 6-12 years**											
Body figure: male		108	Kw	54	0.71 (0.55-0.87)	54	0.68 (0.51-0.84)	67	0.65 (0.48-0.82)	40	0.74 (0.61-0.86)
Body figure: female		28	Kw	14	0.84 (0.70-0.98)	14	0.86 (0.68-1.00)	22	0.89 (0.82-0.97)	6	0.40 (-0.39, 1.00)
**FFQ at age 6-12 years**											
All meat	freq	137	ICC	70	0.60 (0.34-0.75)	67	0.75 (0.59-0.84)	90	0.69 (0.52-0.80)	46	0.45 (0.02-0.69) †

	port	131	ICC	66	0.43 (0.08-0.65) +	65	0.48 (0.15-0.68)	86	0.33 (-0.03, 0.57)	45	0.60 (0.28-0.78)
Animal liver	freq	138	ICC	70	0.59 (0.34-0.74)	68	0.78 (0.65-0.87)	91	0.61 (0.41-0.74)	46	0.85 (0.73-0.92)
	port	99	ICC	49	0.39 (-0.06, 0.65)	50	-0.09 (-0.95, 0.39)	60	0.18 (-0.37, 0.51)	39	0.24 (-0.46, 0.60)
All fish	freq	138	ICC	69	0.63 (0.41-0.77)	69	0.72 (0.55-0.83)	91	0.60 (0.40-0.74)	46	0.83 (0.69-0.91)
	port	136	ICC	69	0.45 (0.12-0.66)	67	0.46 (0.13-0.67)	90	0.42 (0.13-0.62)	46	0.57 (0.23-0.76)
All fruit	freq	138	ICC	69	0.69 (0.51-0.81)	69	0.81 (0.70-0.88)	90	0.73 (0.59-0.82)	47	0.75 (0.55-0.86)
	port	119	ICC	62	0.59 (0.32-0.75)	57	0.52 (0.19-0.72)	75	0.63 (0.42-0.77)	44	0.27 (-0.35, 0.61)
All vegetable	freq	139	ICC	70	0.76 (0.60-0.86)	69	0.74 (0.58-0.84)	91	0.77 (0.65-0.85)	47	0.71 (0.47-0.84)
	port	136	ICC	69	0.71 (0.54-0.82)	67	0.43 (0.07-0.65) +	89	0.61 (0.41-0.75)	47	0.59 (0.27-0.77)
Fresh milk	freq	139	ICC	70	0.63 (0.41-0.77)	69	0.76 (0.60-0.86)	91	0.64 (0.45-0.76)	47	0.70 (0.15-0.87)
	port	70	ICC	35	0.76 (0.52-0.88)	35	0.55 (0.10-0.77)	33	0.78 (0.55-0.89)	37	0.43 (-0.13, 0.71)
Powdered milk	freq	138	ICC	70	0.39 (0.02-0.62) +	68	0.63 (0.39-0.77)	90	0.47 (0.20-0.65)	47	0.55 (0.19-0.75)
	port	30	ICC	17	0.40 (-0.67, 0.78)	13	0.40 (-1.14, 0.82)	16	0.28 (-0.98, 0.74)	14	0.58 (-0.27, 0.86)
Soybean milk	freq	139	ICC	70	0.64 (0.41-0.77)	69	0.83 (0.73-0.90)	91	0.69 (0.52-0.79)	47	0.82 (0.67-0.90)
	port	82	ICC	39	0.60 (0.24-0.79)	43	0.43 (-0.04, 0.69)	44	0.39 (-0.13, 0.67)	38	0.68 (0.37-0.83)
Type of salted fish		140	K	70	0.34 (0.21-0.48)	70	0.21 (0.08-0.35) +	92	0.22 (0.10-0.33)	47	0.39 (0.21-0.56)
Mouldy salted fish	freq	33	ICC	18	0.77 (0.37-0.91)	15	0.73 (0.15-0.91)	19	0.77 (0.39-0.91)	13	0.54 (-0.62, 0.86)
	port	33	ICC	18	0.84 (0.58-0.94)	15	0.86 (0.58-0.95)	20	0.80 (0.48-0.92)	13	0.95 (0.83-0.98)
Firmed salted fish	freq	29	ICC	16	0.84 (0.54-0.94)	13	0.84 (0.48-0.95)	17	0.81 (0.49-0.93)	12	0.71 (0.05-0.91) +
	port	29	ICC	16	0.91 (0.73-0.97)	13	0.51 (-0.70, 0.85)	17	0.76 (0.31-0.91)	12	0.47 (-1.03, 0.85)
Other types of salted fish	freq	0	ICC	0	-	0	-	0	-	0	-
	port	0	ICC	0	-	0	-	0	-	0	-
Any types of salted fish	freq	9	ICC	4	0.90 (-0.02, 0.99)	5	0.96 (0.66-1.00)	6	0.96 (0.60-0.99)	3	-
	port	8	ICC	4	-	4	0.89 (-0.11, 0.99)	6	1.00	2	0.89 (-0.32, 1.00)
Preserved meat	freq	137	ICC	69	0.69 (0.50-0.81)	68	0.61 (0.37-0.76)	90	0.64 (0.46-0.77)	46	0.71 (0.48-0.84)
	port	110	ICC	54	0.59 (0.29-0.76)	56	0.71 (0.50-0.83)	67	0.74 (0.58-0.84)	43	0.54 (0.15-0.75)
Preserved egg	freq	139	ICC	69	0.73 (0.56-0.83)	70	0.71 (0.50-0.83)	91	0.52 (0.27-0.68)	47	0.84 (0.72-0.91)
	port	111	ICC	52	0.61 (0.32-0.78)	59	0.66 (0.43-0.80)	70	0.66 (0.45-0.79)	41	0.60 (0.24-0.79)

Preserved vegetable	freq	138	ICC	69	0.62 (0.39-0.76)	69	0.71 (0.53-0.82)	90	0.61 (0.41-0.74)	47	0.78 (0.60-0.88)
	port	98	ICC	46	0.49 (0.07-0.72) ǂ	52	0.58 (0.26-0.76)	64	0.59 (0.33-0.75)	34	0.42 (-0.19, 0.71)
Preserved fruit	freq	138	ICC	69	0.62 (0.45-0.75)	69	0.61 (0.44-0.74)	90	0.64 (0.50-0.75)	47	0.57 (0.34-0.74)
	port	81	ICC	44	0.70 (0.44-0.83)	37	0.45 (-0.08, 0.72)	51	0.73 (0.53-0.85)	30	0.33 (-0.39, 0.68)
**FFQ at age 13-18 years**											
Frequency of deep-fried food (Never/Less than once a month/Once a month/1-3 per week/4-6 per week/1-2 per day/3+ times per day)		140	Kw	70	0.25 (-0.10, 0.60)	70	0.36 (0.15-0.57)	92	0.25 (-0.01, 0.50)	47	0.50 (0.31-0.69)
Frequency of barbeque meat (Never/Less than once a month/Once a month/1-3 per week/4-6 per week/1-2 per day/3+ times per day)		140	Kw	70	0.46 (0.26-0.65)	70	0.60 (0.43-0.77)	92	0.46 (0.30-0.61)	47	0.68 (0.47-0.89)
Ever consumed burnt Chicken/duck/goose/pork skin (Yes/No)		140	K	70	0.32 (0.15-0.49)	70	0.30 (0.12-0.48)	92	0.30 (0.15-0.45)	47	0.30 (0.08-0.52) ǂ
Red meat	freq	139	ICC	70	0.45 (0.25-0.62)	69	0.54 (0.34-0.68)	91	0.46 (0.28-0.60)	47	0.48 (0.22-0.67)
	port	138	ICC	69	0.46 (0.25-0.63)	69	0.30 (0.08-0.50) ǂ	91	0.37 (0.18-0.53)	47	0.42 (0.16-0.63)
Poultry	freq	139	ICC	70	0.47 (0.27-0.63)	69	0.67 (0.52-0.78)	91	0.49 (0.32-0.63)	47	0.61 (0.40-0.76)
	port	131	ICC	64	0.51 (0.31-0.67)	67	0.36 (0.13-0.55)	85	0.48 (0.30-0.63)	46	0.29 (0.02-0.53) ǂ
Animal liver	freq	139	ICC	70	0.55 (0.36-0.69)	69	0.59 (0.42-0.75)	91	0.58 (0.43-0.70)	47	0.54 (0.31-0.72)
	port	102	ICC	50	0.28 (-0.01, 0.52)	52	0.57 (0.36-0.73)	63	0.39 (0.16-0.58)	39	0.58 (0.33-0.75)
Oily fish	freq	139	ICC	70	0.60 (0.42-0.73)	69	0.59 (0.42-0.73)	91	0.60 (0.45-0.72)	47	0.58 (0.35-0.74)
	port	79	ICC	37	0.65 (0.42-0.80)	42	0.38 (0.09-0.61) ǂ	47	0.47 (0.21-0.66)	32	0.64 (0.37-0.81)
Non-oily fish	freq	139	ICC	70	0.60 (0.43-0.73)	69	0.51 (0.31-0.66)	91	0.59 (0.44-0.71)	47	0.49 (0.24-0.68)

	port	115	ICC	57	0.31 (0.06-0.53) †	58	0.22 (-0.04, 0.45)	72	0.32 (0.10-0.51)	43	0.18 (-0.11, 0.45)
Shellfish	freq	139	ICC	70	0.69 (0.55-0.80)	69	0.51 (0.31-0.66)	91	0.59 (0.44-0.71)	47	0.61 (0.39-0.76)
	port	114	ICC	55	0.49 (0.26-0.67)	59	0.30 (0.05-0.52) †	70	0.24 (0.01-0.44) †	44	0.58 (0.34-0.75)
Leafy green vegetable	freq	138	ICC	69	0.57 (0.39-0.71)	69	0.25 (0.02-0.46) †	90	0.44 (0.25-0.59)	47	0.42 (0.15-0.63)
	port	137	ICC	68	0.58 (0.40-0.72)	69	0.29 (0.06-0.49) †	90	0.45 (0.27-0.60)	47	0.42 (0.16-0.63)
Other vegetable	freq	140	ICC	70	0.28 (0.06-0.48) †	70	0.48 (0.27-0.64)	92	0.29 (0.09-0.46) †	47	0.62 (0.40-0.77)
	port	129	ICC	64	0.60 (0.41-0.73)	65	0.60 (0.42-0.74)	83	0.60 (0.44-0.72)	46	0.60 (0.37-0.75)
Carrot	freq	140	ICC	70	0.64 (0.48-0.76)	70	0.64 (0.47-0.76)	92	0.60 (0.45-0.72)	47	0.72 (0.55-0.83)
	port	108	ICC	53	0.38 (0.13-0.59)	55	0.43 (0.19-0.63)	63	0.32 (0.08-0.52) †	45	0.56 (0.32-0.73)
Tomato	freq	139	ICC	70	0.54 (0.35-0.69)	69	0.52 (0.33-0.68)	92	0.48 (0.30-0.62)	46	0.71 (0.53-0.83)
	port	124	ICC	60	0.40 (0.16-0.59)	64	0.41 (0.19-0.60)	79	0.47 (0.28-0.63)	45	0.33 (0.04-0.57) †
Citrus fruit	freq	139	ICC	70	0.65 (0.49-0.77)	69	0.48 (0.28-0.65)	91	0.56 (0.40-0.69)	47	0.70 (0.53-0.82)
	port	127	ICC	60	0.45 (0.22-0.63)	67	0.49 (0.29-0.65)	80	0.50 (0.32-0.65)	47	0.44 (0.17-0.64)
Other fruits	freq	140	ICC	70	0.62 (0.46-0.75)	70	0.38 (0.16-0.56)	92	0.54 (0.38-0.67)	47	0.57 (0.34-0.74)
	port	130	ICC	61	0.40 (0.16-0.59)	69	0.69 (0.13-0.55)	83	0.42 (0.23-0.58)	47	0.25 (-0.04, 0.50)
Fresh milk	freq	139	ICC	70	0.67 (0.52-0.78)	69	0.78 (0.66-0.86)	91	0.64 (0.50-0.75)	47	0.80 (0.66-0.88)
	port	79	ICC	38	0.54 (0.27-0.74)	41	0.49 (0.23-0.69)	43	0.31 (0.03-0.56) †	36	0.79 (0.62-0.89)
Powdered milk	freq	139	ICC	70	0.34 (0.12-0.53)	69	0.57 (0.39-0.71)	91	0.49 (0.32-0.63)	47	0.34 (0.06-0.57) †
	port	30	ICC	19	0.32 (-0.09, 0.66)	11	0.48 (-0.15, 0.83)	17	0.21 (-0.20, 0.59)	13	0.51 (-0.04, 0.82)
Dairy products	freq	140	ICC	70	0.62 (0.46-0.75)	70	0.67 (0.51-0.78)	92	0.59 (0.44-0.71)	47	0.81 (0.68-0.89)
	port	106	ICC	52	0.47 (0.24-0.66)	54	0.35 (0.10-0.56)	62	0.41 (0.19-0.60)	44	0.17 (-0.13, 0.44)
Eggs	freq	140	ICC	70	0.63 (0.46-0.75)	70	0.49 (0.29-0.65)	92	0.55 (0.38-0.67)	47	0.64 (0.40-0.79)
	port	132	ICC	64	0.12 (-0.13, 0.36)	68	0.39 (0.17-0.57)	85	0.18 (-0.03, 0.38)	47	0.51 (0.26-0.69)
Tofu	freq	140	ICC	70	0.59 (0.42-0.73)	70	0.58 (0.40-0.72)	92	0.55 (0.39-0.68)	47	0.71 (0.53-0.83)
	port	126	ICC	61	0.54 (0.33-0.70)	65	0.38 (0.15-0.57)	80	0.49 (0.31-0.64)	46	0.40 (0.13-0.62)
Soybean milk	freq	137	ICC	69	0.59 (0.38-0.70)	68	0.69 (0.54-0.80)	89	0.59 (0.44-0.71)	47	0.74 (0.58-0.85)
	port	96	ICC	48	0.39 (0.13-0.60)	48	0.48 (0.24-0.67)	55	0.42 (0.19-0.62)	41	0.47 (0.20-0.67)
Bean curd	freq	139	ICC	70	0.65 (0.49-0.77)	69	0.70 (0.56-0.80)	91	0.66 (0.53-0.76)	47	0.73 (0.57-0.84)

	port	112	ICC	54	0.43 (0.19-0.63)	58	0.27 (0.01-0.49) ǂ	67	0.43 (0.21-0.61)	45	0.23 (-0.06, 0.49)
Types of salted fish		139	K	70	0.42 (0.28-0.55)	69	0.21 (0.07-0.34) ǂ	91	0.29 (0.17-0.42)	47	0.35 (0.18-0.52)
Mouldy salted fish	freq	26	ICC	15	0.82 (0.53-0.93)	11	0.91 (0.73-0.98)	18	0.84 (0.58-0.94)	8	0.68 (-0.03, 0.93)
	port	26	ICC	15	0.64 (0.23-0.86)	11	0.83 (0.51-0.95)	18	0.58 (0.15-0.82)	8	0.89 (0.58-0.98)
Firmed salted fish	freq	36	ICC	21	0.70 (0.39-0.86)	15	0.42 (-0.11, 0.76)	24	0.61 (0.28-0.81)	12	0.49 (-0.04, 0.81)
	port	34	ICC	20	0.51 (0.10-0.78)	14	0.36 (-0.22, 0.74)	22	0.56 (0.18-0.79)	12	0.42 (-0.19, 0.79)
Other types of salted fish	freq	0	ICC	0	-	0	-	0	-	0	-
	port	0	ICC	0	-	0	-	0	-	0	-
Any types of salted fish	freq	13	ICC	5	0.92 (0.53-0.99)	8	0.95 (0.77-0.99)	7	0.95 (0.72-0.99)	6	0.88 (0.44-0.98)
	port	13	ICC	5	0.20 (-1.15, 0.89)	8	0.26 (-0.60, 0.80)	7	-0.75 (-1.22, 0.22)	6	1.00
Preserved seafood	freq	139	ICC	70	0.65 (0.48-0.76)	69	0.46 (0.26-0.63)	91	0.57 (0.41-0.69)	47	0.54 (0.30-0.71)
	port	112	ICC	55	0.48 (0.25-0.66)	57	0.43 (0.19-0.62)	69	0.38 (0.16-0.57)	43	0.57 (0.33-0.74)
Preserved vegetable	freq	139	ICC	70	0.57 (0.39-0.71)	69	0.62 (0.45-0.75)	91	0.55 (0.39-0.68)	47	0.72 (0.55-0.83)
	port	114	ICC	54	0.57 (0.36-0.73)	60	0.37 (0.13-0.57)	73	0.43 (0.22-0.60)	41	0.54 (0.28-0.73)
Preserved fruit	freq	140	ICC	70	0.70 (0.55-0.80)	70	0.61 (0.43-0.74)	92	0.64 (0.50-0.75)	47	0.70 (0.51-0.82)
	port	90	ICC	45	0.51 (0.26-0.70)	45	0.25 (-0.05, 0.51)	54	0.49 (0.26-0.67)	36	0.19 (-0.13, 0.48)
Preserved egg	freq	139	ICC	70	0.66 (0.48-0.78)	69	0.60 (0.42-0.73)	91	0.64 (0.50-0.75)	47	0.57 (0.34-0.73)
	port	112	ICC	56	0.61 (0.42-0.75)	56	0.36 (0.11-0.57)	70	0.51 (0.31-0.66)	42	0.44 (0.16-0.66)
Preserved meat	freq	139	ICC	70	0.49 (0.29-0.65)	69	0.52 (0.33-0.68)	91	0.48 (0.30-0.62)	47	0.55 (0.31-0.72)
	port	113	ICC	55	0.54 (0.33-0.71)	58	0.43 (0.20-0.62)	68	0.48 (0.27-0.65)	45	0.50 (0.25-0.69)
Processed meat	freq	140	ICC	70	0.72 (0.59-0.82)	70	0.57 (0.39-0.71)	92	0.65 (0.52-0.75)	47	0.60 (0.37-0.75)
	port	100	ICC	49	0.35 (0.07-0.57) ǂ	51	0.48 (0.24-0.67)	59	0.45 (0.21-0.63)	41	0.38 (0.08-0.61) ǂ
Condiments	freq	139	ICC	70	0.40 (0.18-0.58)	69	0.40 (0.19-0.58)	91	0.41 (0.22-0.56)	47	0.40 (0.14-0.61)
	port	125	ICC	60	0.25 (-0.01, 0.47)	65	0.27 (0.03-0.49) ǂ	78	0.26 (0.04-0.46)	47	0.31 (0.03-0.55) ǂ
Green/white tea	freq	140	ICC	70	0.41 (0.19-0.59)	70	0.56 (0.37-0.70)	92	0.50 (0.33-0.64)	47	0.43 (0.17-0.64)
	port	77	ICC	38	0.26 (-0.06, 0.53)	39	0.40 (0.10-0.64)	42	0.46 (0.20-0.67)	35	0.11 (-0.24, 0.43)
Oolong tea	freq	140	ICC	70	0.68 (0.53-0.79)	70	0.60 (0.43-0.73)	92	0.69 (0.56-0.78)	47	0.50 (0.25-0.69)
	port	70	ICC	36	0.13 (-0.17, 0.43)	34	0.04 (-0.31, 0.37)	39	0.34 (0.05-0.58) ǂ	31	-0.42 (-0.69, -0.06)

Red/black tea	freq	140	ICC	70	0.57 (0.38-0.71)	70	0.70 (0.55-0.80)	92	0.58 (0.43-0.70)	47	0.74 (0.57-0.84)
	port	88	ICC	40	0.37 (0.07-0.61) +	48	0.19 (-0.10, 0.45)	50	0.41 (0.14-0.61)	38	0.10 (-0.22, 0.40)
Cantonese-style milk tea	freq	140	ICC	70	0.80 (0.70-0.87)	70	0.58 (0.41-0.72)	92	0.67 (0.53-0.77)	47	0.80 (0.67-0.88)
	port	70	ICC	39	0.17 (-0.16, 0.46)	31	0.36 (0.01-0.63) +	35	0.30 (-0.04, 0.57)	34	-0.04 (-0.38, 0.30)
Coffee	freq	139	ICC	70	0.73 (0.59-0.82)	69	0.61 (0.43-0.74)	91	0.58 (0.43-0.71)	47	0.79 (0.65-0.88)
	port	44	ICC	26	0.42 (0.05-0.69) +	18	0.35 (-0.12, 0.69)	20	0.48 (0.04-0.76) +	24	0.25 (-0.18, 0.59)
Chinese herbal tea	freq	139	ICC	70	0.63 (0.47-0.76)	69	0.63 (0.46-0.75)	91	0.64 (0.50-0.75)	47	0.61 (0.40-0.76)
	port	109	ICC	57	0.61 (0.42-0.75)	52	0.13 (-0.16, 0.39)	69	0.26 (0.03-0.47) +	40	0.47 (0.18-0.68)
**FFQ at age 19-30 years**											
Any changes to your diet during 19-30 years old, compared with 13-18's (Yes/No)		135	K	69	0.31 (0.07-0.54) +	66	0.50 (0.28-0.72)	88	0.36 (0.15-0.57)	46	0.44 (0.17-0.71)
If yes, which food items											
All meat (yes/no)		77	K	40	0.20 (0.10-0.30)	37	0.23 (0.14-0.32)	51	0.22 (0.14-0.31)	25	0.19 (0.08-0.30) +
Animal liver (yes/no)		44	K	20	0.17 (0.06-0.28) +	24	0.14 (0.03-0.24) +	28	0.14 (0.04-0.24) +	15	0.16 (0.02-0.29) +
Seafood (yes/no)		69	K	32	0.15 (0.04-0.25) +	37	0.23 (0.14-0.32)	45	0.16 (0.07-0.26) +	23	0.23 (0.12-0.34)
Vegetable (yes/no)		63	K	26	0.10 (-0.01, 0.21)	37	0.21 (0.11-0.30)	43	0.17 (0.08-0.26) +	19	0.13 (0.01-0.24) +
Fruit (yes/no)		72	K	34	0.14 (0.03-0.24) +	38	0.22 (0.13-0.31)	48	0.18 (0.09-0.27) +	23	0.16 (0.05-0.28) +
Dairy products (yes/no)		52	K	22	0.10 (-0.01, 0.20)	30	0.17 (0.07-0.27) +	31	0.15 (0.06-0.25) +	20	0.11 (-0.01, 0.22)
Egg (yes/no)		49	K	22	0.16 (0.05-0.28) +	27	0.19 (0.09-0.29) +	30	0.20 (0.11-0.29)	18	0.14 (0.02-0.26) +
Soybean products (yes/no)		55	K	25	0.12 (0.01-0.22) +	30	0.19 (0.10-0.29)	36	0.16 (0.06-0.25) +	18	0.14 (0.02-0.26) +
Salted fish (yes/no)		49	K	22	0.20 (0.10-0.31)	27	0.17 (0.07-0.27) +	30	0.17 (0.08-0.27) +	18	0.20 (0.08-0.33) +
Preserved food (yes/no)		50	K	20	0.17 (0.06-0.28) +	30	0.17 (0.07-0.27) +	31	0.16 (0.07-0.25) +	18	0.19 (0.06-0.31) +
Beverage		69	K	39	0.15 (0.05-0.26) +	30	0.20 (0.10-0.29)	47	0.16 (0.07-0.25) +	21	0.20 (0.09-0.32) +
**FFQ 10 years ago**											

Any changes to your diet before 10 years ago, compared with 19-30's	140	K	70	0.55 (0.38-0.71)	70	0.63 (0.48-0.79)	92	0.48 (0.32-0.63)	47	0.82 (0.67-0.96)
If yes, which food items										
All meat (yes/no)	97	K	48	0.18 (-0.03, 0.39)	49	0.36 (0.15-0.56)	69	0.24 (0.06-0.42) †	27	0.35 (0.07-0.63) †
Animal liver (yes/no)	98	K	49	0.20 (-0.01, 0.40)	49	0.33 (0.12-0.54)	70	0.19 (0.02-0.37) †	27	0.44 (0.16-0.73)
Seafood (yes/no)	95	K	46	0.19 (-0.01, 0.39)	49	0.24 (0.03-0.44) †	67	0.13 (-0.05, 0.30)	27	0.41 (0.12-0.69)
Vegetable (yes/no)	97	K	49	0.15 (-0.06, 0.35)	48	0.32 (0.11-0.53)	70	0.16 (-0.02, 0.33)	26	0.42 (0.14-0.70)
Fruit (yes/no)	98	K	49	0.24 (0.03-0.44) †	49	0.19 (-0.03, 0.40)	70	0.21 (0.04-0.39) †	27	0.23 (-0.07, 0.53)
Dairy products (yes/no)	96	K	47	0.17 (-0.06, 0.40)	49	0.40 (0.19-0.60)	70	0.25 (0.07-0.43) †	25	0.37 (0.04-0.70) †
Egg (yes/no)	96	K	48	0.03 (-0.17-0.22)	48	0.31 (0.10-0.51)	69	0.14 (-0.04, 0.32)	26	0.19 (-0.12, 0.50)
Soybean products (yes/no)	97	K	49	0.17 (-0.05, 0.38)	48	0.31 (0.09-0.52) †	70	0.20 (0.02-0.38) †	26	0.33 (0.01-0.66) †
Salted fish (yes/no)	94	K	47	0.17 (-0.05, 0.38)	47	0.31 (0.10-0.52)	68	0.17 (-0.01, 0.35)	25	0.42 (0.13-0.71)
Preserved food (yes/no)	97	K	48	0.19 (-0.02, 0.40)	49	0.26 (0.05-0.47) †	69	0.19 (0.01-0.36) †	27	0.29 (-0.01, 0.59)
Beverage	97	K	48	0.09 (-0.12, 0.29)	49	0.32 (0.12-0.52)	69	0.22 (0.04-0.40) †	27	0.18 (-0.13, 0.48)

* only subjects who filled out the question in both questionnaires were included in the analysis.

ICC: intra-class correlation coefficient. K: Cohen's kappa. Kw: Weighted kappa. Weighted kappa. Freq: frequency (Never/Less than once a month/Once a month/1-3 per week/4-6 per week/1-2 per day/3+ per day). Differences by time between questionnaire or by education (difference between coefficients >0.30, and were tested by a Fisher Z transformation) were shade. (Coefficients: 0-0.20 [poor]; 0.20-0.40 [fair]; 0.40-0.60 [moderate]; 0.60-0.80 [substantial]; 0.80-1.00 [almost perfect]). The format of 95% cell: (x1-x2) indicating p<0.01, unless otherwise stated (†: indicating p between 0.05 and0.01); the other format: (-x1, x2) indicating p-value>0.05.

Supplementary Table s4. Questionnaire items and reliability coefficients with their 95% confidence intervals by age groups at first questionnaire

Questionnaire item	N*	Method	Age groups at the first questionnaire					
				Age at 25-44 years (N=41)		Age at 45-59 years (N=63)		Age at 60+ years (N=36)
			n		n		n	
Siblings								
No. of older brother	70	ICC	13	1.00	31	0.40 (0.07-0.66) ǂ	26	0.61 (0.30-0.81)
No. of younger brother	68	ICC	17	1.00 (1.00-1.00)	32	0.99 (0.97-0.99)	19	1.00
No. of older sister	70	ICC	17	1.00	35	0.99 (0.98-1.00)	18	0.58 (0.17-0.82)
No. of younger sister	64	ICC	12	0.90 (0.70-0.97)	32	0.38 (0.05-0.64) ǂ	20	0.75 (0.48-0.89)
Marital status (single/married/divorced/widowed)	140	K	41	1.00 (1.00-1.00)	63	0.78 (0.61-0.95)	36	0.87 (0.68-1.00)
Income								
Employment status (self-employed/employed/retired/housewives/student/unemployed)	138	K	41	0.31 (0.05-0.57) ǂ	61	0.80 (0.66-0.93)	36	0.58 (0.36-0.79)
Personal income (none/less than 15k/15-24.9k/25-39.9k/40k or above)	138	Kw	40	0.53 (0.20-0.86)	62	0.77 (0.58-0.95)	36	0.74 (0.54-0.94)
Household income (none/less than 15k/15-24.9k/25-39.9k/40k or above)	133	Kw	40	0.44 (0.13-0.76)	62	0.64 (0.41-0.86)	31	0.67 (0.43-0.91)
Off-springs								
No. of son	62	ICC	9	1.00	25	1.00	28	0.99 (0.97-0.99)
No. of daughter	59	ICC	9	1.00	31	1.00	19	1.00 (1.00-1.00)
None	45	K	41	1.00 (1.00-1.00)	63	0.92 (0.81-1.00)	36	0.84 (0.54-1.00)
Birth place (HK/Macau/Guangxi/Guangdong/Fujian/Hunan/Others/unknown)	140	K	41	1.00 (1.00-1.00)	63	0.95 (0.86-1.00)	36	0.86 (0.72-1.00)
Mother's birth place (HK/Macau/Guangxi/Guangdong/Fujian/Hunan/Others/unknown)	140	K	41	0.72 (0.56-0.89)	63	0.76 (0.61-0.91)	36	0.75 (0.56-0.95)
Housing type at 10 years old (Temporary/Public/Home Ownership Scheme/Private (owner)/Private (rent)/Boat/Shanty/Stone/Village/Others)	140	K	41	0.74 (0.58-0.89)	63	0.80 (0.69-0.91)	36	0.72 (0.55-0.88)
Education (None/Old-style school/Primary/Secondary 1-3/Secondary 4-5/Matriculation F.6-7/Technical)								
Subject's	140	K	41	0.93 (0.84-1.00)	63	0.83 (0.72-0.94)	36	0.86 (0.72-0.99)

Subject's father	140	K	41	0.58 (0.40-0.76)	63	0.49 (0.34-0.65)	36	0.53 (0.34-0.71)
Subject's mother	140	K	41	0.68 (0.52-0.84)	63	0.69 (0.55-0.82)	36	0.58 (0.38-0.78)
Cancer history (Yes/No)								
Men	140	K	41	0.75 (0.52-0.98)	63	0.91 (0.78-1.00)	36	0.88 (0.72-1.00)
Women	140	K	41	0.93 (0.78-1.00)	63	1.00 (1.00-1.00)	36	1.00 (1.00-1.00)
Mother experienced illness (Yes/No)								
During pregnant	140	K	41	0.37 (0.13-0.61)	63	0.43 (0.23-0.63)	36	0.61 (0.36-0.87)
During delivery	140	K	41	0.39 (0.13-0.65)	63	0.30 (0.07-0.54) ┼	36	0.56 (0.25-0.87)
Birth delivery mode (Natural birth/Caesarean section/Unknown)	140	K	41	0.54 (0.25-0.83)	63	-0.06 (-0.10, -0.01)	36	0.40 (0.00-0.79) ┼
Single birth or multiple births	139	K	41	-	63	-	35	-
Birth period (Premature/Full-term/Post-term)	140	K	41	0.65 (0.42-0.87)	63	0.68 (0.44-0.91)	36	0.62 (0.35-0.90)
Birth weight (kg)	140	ICC	41	0.46 (0.18-0.67)	63	0.68 (0.52-0.79)	36	0.57 (0.30-0.75)
Breastfed (Yes/No/Unknown)	140	K	41	0.59 (0.40-0.77)	63	0.60 (0.46-0.74)	36	0.53 (0.29-0.78)
Breastfed period (months)	140	ICC	41	0.36 (0.08-0.60) ┼	63	0.41 (0.18-0.60)	36	0.58 (0.31-0.76)
Family history of cancer (Yes/No)	136	K	40	0.77 (0.56-0.98)	62	0.86 (0.72-0.99)	34	0.94 (0.83-1.00)
Family history of nasopharyngeal carcinoma (Yes/No)	75	K	27	1.00 (1.00-1.00)	26	1.00 (1.00-1.00)	22	1.00 (1.00-1.00)
Omega-3								
Ever/never	140	K	41	0.70 (0.48-0.92)	63	0.55 (0.35-0.76)	36	0.47 (0.18-0.76)
Age started, years	44	ICC	13	0.63 (0.13-0.87)	22	0.72 (0.44-0.87)	9	0.33 (-0.44, 0.80)
Duration, years	43	ICC	13	0.48 (-0.08, 0.81)	21	0.68 (0.37-0.86)	9	0.42 (-0.34, 0.84)
Frequency	36	ICC	10	0.54 (-0.14, 0.87)	18	0.53 (0.10-0.80)	8	0.76 (0.19-0.95)
(Less than once a month/Once a month/1-3 per week/4-6 per week/1-2 per day/3+ per day)								
Vitamins								
Ever/never	140	K	41	0.47 (0.22-0.72)	63	0.52 (0.28-0.75)	36	0.16 (-0.16, 0.48)
Multi-vitamins								

Ever/never	140	K	41	0.69 (0.35-1.00)	63	0.45 (0.08-0.82) †	36	0.53 (0.06-0.99) †
Age started, years	9	ICC	4	-0.23 (-0.29, 0.33)	3	0.77 (-0.16, 0.99)	2	-0.57 (-1.00, 0.96)
Duration, years	9	ICC	4	1.00 (0.98-1.00)	3	0.99 (0.72-1.00)	2	0.83 (-0.08, 1.00)
Frequency	9	ICC	4	0.57 (-0.96, 0.97)	3	0.58 (-1.72, 0.99)	2	-
(Less than once a month/Once a month/1-3 per week/4-6 per week/1-2 per day/3+ per day)								
Vitamins A								
Ever/never	140	K	41	-0.02 (-0.06, 0.01)	63	-	36	-
Age started, years	0	ICC	0	-	0	-	0	-
Duration, years	0	ICC	0	-	0	-	0	-
Frequency	0	ICC	0	-	0	-	0	-
(Less than once a month/Once a month/1-3 per week/4-6 per week/1-2 per day/3+ per day)								
Vitamins B								
Ever/never	140	K	41	0.23 (-0.21, 0.68)	63	1.00 (1.00-1.00)	36	-0.08 (-0.16, -0.00)
Age started, years	6	ICC	1	-	5	0.28 (-0.48, 0.88)	0	-
Duration, years	6	ICC	1	-	5	0.76 (-0.22, 0.97)	0	-
Frequency	5	ICC	1	-	4	0.39 (-0.66, 0.94)	0	-
(Less than once a month/Once a month/1-3 per week/4-6 per week/1-2 per day/3+ per day)								
Vitamins C								
Ever/never	140	K	41	0.27 (-0.09, 0.63)	63	0.53 (0.21-0.85)	36	-0.07 (-0.14, -0.00)
Age started, years	7	ICC	3	-0.70 (-1.61, 0.88)	4	0.52 (-0.23, 0.96)	0	-
Duration, years	7	ICC	3	1.00 (0.85-1.00)	4	-0.14 (-0.95, 0.85)	0	-
Frequency	7	ICC	3	0.96 (0.46-1.00)	4	0.75 (-0.09, 0.98)	0	-
(Less than once a month/Once a month/1-3 per week/4-6 per week/1-2 per day/3+ per day)								
Vitamins D								

Ever/never consumed	140	K		41	0.66 (0.03-1.00) †	63	-0.04 (-0.09, -0.00)	36	-0.05 (-0.12, 0.03)
Age started, years	1	ICC	-	1	-	0	-	0	-
Duration, years	1	ICC	-	1	-	0	-	0	-
Frequency	1	ICC	-	1	-	0	-	0	-
(Less than once a month/Once a month/1-3 per week/4-6 per week/1-2 per day/3+ per day)									
Vitamins E									
Ever/never	140	K		41	0.47 (-0.15, 1.00)	63	1.00 (1.00-1.00)	36	-0.06 (-0.12, -0.00)
Age started, years	5	ICC		1	-	4	0.52 (-0.22, 0.95)	0	-
Duration, years	5	ICC		1	-	4	0.74 (-0.77, 0.98)	0	-
Frequency	5	ICC		1	-	4	0.83 (-0.43, 0.99)	0	-
(Less than once a month/Once a month/1-3 per week/4-6 per week/1-2 per day/3+ per day)									
Oral health									
No. of dental caries	138	Kw		40	0.47 (0.16-0.77)	63	0.66 (0.49-0.82)	35	0.46 (0.16-0.76)
No. of teeth extracted due to dental caries	92	Kw		26	0.60 (0.26-0.94)	41	0.77 (0.65-0.88)	25	0.44 (0.04-0.84) †
Periodontal disease (yes/no)	138	K		40	0.94 (0.81-1.00)	62	0.79 (0.64-0.95)	36	0.61 (0.36-0.87)
No. of teeth extracted due to periodontal disease	42	Kw		10	0.83 (0.69-0.97)	19	0.81 (0.62-1.00)	13	0.52 (0.12-0.92)
Active smoking									
Ever/never	139	K		40	0.95 (0.85-1.00)	63	0.89 (0.76-1.00)	36	0.94 (0.83-1.00)
Time between wake up and first cig (Less than 5/6-30/31-60/60+ minutes)	140	Kw		41	0.66 (0.39-0.92)	63	0.82 (0.65-0.99)	36	0.91 (0.80-1.00)
Age starting years	46	ICC		16	0.94 (0.83-0.98)	17	0.96 (0.89-0.99)	13	0.56 (0.07-0.84) †
Smoking amount									
Before age of 18 years	24	ICC		11	0.48 (-0.17, 0.83)	7	0.76 (0.10-0.96)	6	0.56 (-0.19, 0.92)
After age of 18 years	46	ICC		16	0.59 (0.16-0.83)	17	0.83 (0.59-0.93)	13	0.80 (0.48-0.94)
Age at quitting	32	ICC		9	0.99 (0.97-1.00)	12	0.99 (0.96-1.00)	11	1.00 (0.98-1.00)

Duration of quitting	24	ICC	8	0.30 (-0.50, 0.81)	10	0.72 (0.22-0.92)	6	0.99 (0.96-1.00)
Passive smoking (*household*)								
**childhood**								
No. of co-living smokers	138	ICC	40	0.64 (0.38-0.79)	62	0.86 (0.78-0.91)	36	0.77 (0.59-0.87)
Co-living smoker 1								
Relationship (Father/Mother/Grandfather/Grandmother/Maternal grandfather/Maternal grandmother/Siblings/Others)	82	K	24	0.43 (0.17-0.69)	37	0.76 (0.50-1.00)	21	0.53 (0.14-0.93)
Exposed time/day (Less than 5 minutes/30-60 minutes/1-2 hours/2+ hours)	81	Kw	24	0.63 (0.39-0.87)	36	0.71 (0.52-0.90)	21	0.13 (-0.15, 0.40)
Years of co-living	77	ICC	22	0.50 (0.11-0.76)	34	0.09 (-0.27, 0.41)	21	0.29 (-0.09, 0.62)
Co-living smoker 2								
Relationship (Father/Mother/Grandfather/Grandmother/Maternal grandfather/Maternal grandmother/Siblings/Others)	23	K	5	0.47 (0.09-0.85) †	12	0.68 (0.38-0.99)	6	0.46 (-0.11, 1.00)
Exposed time/day (Less than 5 minutes/30-60 minutes/1-2 hours/2+ hours)	23	Kw	5	0.62 (0.30-0.93)	12	0.55 (0.18-0.92)	6	0.19 (-0.24, 0.62)
Years of co-living	23	ICC	5	-0.15 (-0.82, 0.74)	12	-0.05 (-0.55, 0.50)	6	0.99 (0.92-1.00)
Co-living smoker 3								
Relationship (Father/Mother/Grandfather/Grandmother/Maternal grandfather/Maternal grandmother/Siblings/Others)	8	K	-	-	5	1.00 (1.00-1.00)	3	1.00 (1.00-1.00)
Exposed time/day (Less than 5 minutes/30-60 minutes/1-2 hours/2+ hours)	8	Kw	-	-	5	0.66 (0.43-0.88)	3	0.00 (-0.46, 0.46)
Years of co-living	8	ICC	-	-	5	-	3	0.98 (0.72-1.00)
**adulthood**								
No. of co-living smokers	137	ICC	40	0.51 (0.25-0.71)	61	0.74 (0.60-0.84)	36	0.74 (0.55-0.86)
Co-living smoker 1								
Relationship (Father/Mother/Grandfather/Grandmother/Maternal grandfather/Maternal grandmother/Siblings/Others)	65	K	17	0.60 (0.28-0.92)	30	0.70 (0.42-0.97)	18	0.72 (0.43-1.00)
Exposed time/day (Less than 5 minutes/30-60 minutes/1-2 hours/2+ hours)	63	Kw	16	0.33 (-0.04, 0.70)	29	0.51 (0.18-0.84)	18	0.13 (-0.24, 0.51)
Years of co-living	64	ICC	16	0.50 (0.03-0.79) †	30	0.70 (0.45-0.84)	18	0.65 (0.27-0.85)
Co-living smoker 2								

Relationship (Father/Mother/Grandfather/Grandmother/Maternal grandfather/Maternal grandmother/Siblings/Others)	21	K	2	0.33 (0.03-0.64) †	9	0.54 (0.14-0.94)	10	0.69 (0.34-1.00)
Exposed time/day (Less than 5 minutes/30-60 minutes/1-2 hours/2+ hours)	21	Kw	2	-	9	0.13 (-0.30, 0.55)	10	0.51 (-0.08, 1.00)
Years of co-living	21	ICC	2	0.00 (-0.07, 0.98)	9	-0.06 (-0.62, 0.58)	10	0.80 (0.37-0.95)
Co-living smoker 3								
Relationship (Father/Mother/Grandfather/Grandmother/Maternal grandfather/Maternal grandmother/Siblings/Others)	4	K	-	-	1	-	3	1.00 (1.00-1.00)
Exposed time/day (Less than 5 minutes/30-60 minutes/1-2 hours/2+ hours)	4	Kw	-	-	1	-	3	0.00 (-0.61, 0.94)
Years of co-living	4	ICC	-	-	1	-	3	0.90 (-0.57, 1.00)
Passive smoking (*workplace*)								
**childhood (before 18 years old)**								
Ever/never	138	K	41	0.08 (-0.13, 0.29)	61	0.31 (0.13-0.49)	36	0.46 (0.22-0.69)
Time of exposed/day (Less than 5 minutes/30-60 minutes/1-2 hours/2+ hours)	23	Kw	5	0.82 (0.65-0.99)	9	0.26 (-0.31, 0.83)	9	0.46 (-0.14, 1.00)
How long did you work that exposed to passive smoking, years	23	ICC	5	0.21 (-0.74, 0.34)	9	0.38 (-0.41, 0.82)	9	0.88 (0.56-0.97)
**adulthood (after 18 years old)**								
Ever/never	136	K	40	0.61 (0.38-0.84)	61	0.64 (0.43-0.85)	35	0.32 (0.02-0.63) †
Time of exposed/day (Less than 5 minutes/30-60 minutes/1-2 hours/2+ hours)	77	Kw	21	0.66 (0.41-0.91)	39	0.38 (0.10-0.66)	17	0.11 (-0.37, 0.58)
How long did you work that exposed to passive smoking, years	76	ICC	21	0.45 (0.02-0.73) †	38	0.51 (0.23-0.71)	17	0.54 (0.12-0.80)
Occupational hazards								
Exposed/non-exposed	139	K	41	0.80 (0.62-0.99)	62	0.60 (0.41-0.79)	36	0.48 (0.20-0.76)
Dust	139	K	41	0.63 (0.39-0.87)	63	0.73 (0.55-0.90)	36	0.56 (0.31-0.81)
Chemical	139	K	41	0.56 (0.19-0.93)	63	0.28 (0.04-0.53) †	36	0.22 (-0.03, 0.47)
Fumes	139	K	41	0.78 (0.55-1.00)	63	0.44 (0.17-0.71)	36	0.46 (0.22-0.69)
Acid or alkali	139	K	41	0.58 (0.17-0.99)	63	0.64 (0.32-0.96)	36	0.39 (-0.03, 0.80)
Alcohol drinking								
Ever/never	136	K	40	0.70 (0.48-0.92)	60	0.50 (0.29-0.72)	36	0.77 (0.55-0.98)

Ever drink once per month	48	K	16	0.38 (-0.08, 0.83)	21	0.71 (0.42-1.00)	11	0.79 (0.41-1.00)
Age at starting	53	ICC	17	0.71 (0.38-0.88)	24	0.60 (0.26-0.81)	12	0.53 (0.00-0.84) ǂ
Age at stopping	19	ICC	8	0.91 (0.60-0.98)	7	0.98 (0.88-1.00)	4	0.91 (0.36-0.99)
Duration of stopping drinking	4	ICC	2	0.99 (0.71-1.00)	1	-	1	-
Red wine								
Frequency (Less than once a month/Once a month/1-3 per week/4-6 per week/1-2 per day/3+ per day)	23	ICC	8	0.70 (0.13-0.93)	12	0.77 (0.39-0.93)	3	0.00 (-1.85, 0.96)
Portion, glasses	16	ICC	4	0.73 (-0.82, 0.98)	9	0.00 (-0.75, 0.66)	3	1.00
white wine								
Frequency (Less than once a month/Once a month/1-3 per week/4-6 per week/1-2 per day/3+ per day)	7	ICC	2	0.71 (-0.09, 1.00)	4	1.00	1	-
Portion, glasses	7	ICC	2	1.00	4	0.20 (-1.55, 0.93)	1	-
beer								
Frequency (Less than once a month/Once a month/1-3 per week/4-6 per week/1-2 per day/3+ per day)	47	ICC	13	0.94 (0.81-0.98)	24	0.50 (0.12-0.75)	10	0.70 (0.20-0.91)
Portion (unit: pint)	1	ICC	0	-	2	0.00 (-1.00, 1.00)	0	-
Portion (unit: 375ml/can)	8	ICC	2	0.92 (-0.22, 1.00)	4	0.33 (-0.70, 0.94)	2	0.00 (-1.00, 1.00)
Portion (unit: 500ml/can)	1	ICC	2	1.00	3	0.00 (-2.15, 0.96)	0	-
Portion (unit: 330ml/bottle)	3	ICC	2	0.39 (-0.93, 1.00)	1	-	0	-
Portion (unit: 600ml/bottle)	6	ICC	1	-	3	0.47 (-1.05, 0.98)	2	-
spirits								
Frequency (Less than once a month/Once a month/1-3 per week/4-6 per week/1-2 per day/3+ per day)	14	ICC	6	0.25 (-0.71, 0.85)	6	0.72 (-0.18, 0.96)	2	1.00
Portion (unit: Chinese-style glass)	2	ICC	1	-	0	-	1	-
Portion (unit: Western-style glass)	4	ICC	3	0.99 (0.83-1.00)	1	-	0	-
Sun exposure								
10 years ago								

Outdoor exercise (Less than once a month/Once a month/1-3 per week/4-6 per week/Daily)	140	Kw	41	-	63	0.46 (0.23-0.69)	36	0.35 (0.02-0.68) ǂ
Duration of sun exposure (Less than 1/2-4/5-7/8-10 hours)	140	Kw	41	-	63	0.37 (0.17-0.57)	36	0.59 (0.38-0.80)
Avoid sun exposure (Never/Ever)	140	K	41	-	63	0.15 (-0.02, 0.31)	36	0.28 (0.08-0.49) ǂ
Protection from sunshine (Never/Ever)	140	K	41	-	63	0.23 (0.06-0.40) ǂ	36	0.26 (0.06-0.47) ǂ
19-30 years ago								
Outdoor exercise (Less than once a month/Once a month/1-3 per week/4-6 per week/Daily)	138	Kw	40	0.33 (0.07-0.60) ǂ	62	0.52 (0.31-0.73)	36	0.44 (0.14-0.74)
Duration of sun exposure (Less than 1/2-4/5-7/8-10 hours)	138	Kw	40	0.16 (-0.14, 0.46)	62	0.51 (0.34-0.69)	36	0.58 (0.26-0.90)
Avoid sun exposure (Never/Ever)	139	K	41	0.26 (0.05-0.47) ǂ	62	0.22 (0.04-0.39) ǂ	36	0.24 (0.05-0.43) ǂ
Protection from sunshine (Never/Ever)	139	K	41	0.21 (0.00-0.42) ǂ	62	0.22 (0.05-0.39) ǂ	36	0.30 (0.03-0.57) ǂ
13-18 years ago								
Outdoor exercise (Less than once a month/Once a month/1-3 per week/4-6 per week/Daily)	138	Kw	40	0.45 (0.18-0.71)	62	0.37 (0.13-0.60)	36	0.79 (0.61-0.97)
Duration of sun exposure (Less than 1/2-4/5-7/8-10 hours)	138	Kw	40	0.60 (0.38-0.82)	62	0.34 (0.07-0.61) ǂ	36	0.73 (0.54-0.92)
Avoid sun exposure (Never/Ever)	139	K	41	0.44 (0.22-0.67)	62	0.25 (0.08-0.42) ǂ	36	0.14 (-0.16, 0.45)
Protection from sunshine (Never/Ever)	139	K	41	0.16 (-0.07, 0.38)	62	0.25 (0.08-0.43) ǂ	36	0.21 (-0.07, 0.48)
6-12 years ago								
Outdoor exercise (Less than once a month/Once a month/1-3 per week/4-6 per week/Daily)	137	Kw	40	0.41 (0.10-0.72)	61	0.32 (0.10-0.54)	36	0.69 (0.46-0.93)
Duration of sun exposure (Less than 1/2-4/5-7/8-10 hours)	137	Kw	40	0.66 (0.47-0.84)	61	-0.04 (-0.27, 0.19)	36	0.46 (0.13-0.78)
Avoid sun exposure (Never/Ever)	139	K	41	0.26 (0.02-0.49) ǂ	62	0.25 (0.08-0.41) ǂ	36	0.30 (-0.02, 0.63)
Protection from sunshine (Never/Ever)	139	K	41	0.27 (0.02-0.52) ǂ	62	0.33 (0.14-0.52)	36	-0.06 (-0.11, -0.01)
Skin tone								
Current								
Face	138	Kw	41	0.64 (0.44-0.83)	61	0.48 (0.29-0.68)	36	0.61 (0.36-0.86)
Hand	139	Kw	41	0.32 (0.01-0.63) ǂ	62	0.42 (0.15-0.69)	36	0.60 (0.38-0.83)

10 years ago: hand		91	Kw	0	-	55	0.48 (0.28-0.67)	36	0.59 (0.41-0.78)
Age 19-30 years: hand		138	Kw	40	0.16 (-0.11, 0.43)	62	0.62 (0.47-0.77)	36	0.52 (0.28-0.75)
Age 13-18 years: hand		137	Kw	41	0.41 (0.17-0.65)	60	0.43 (0.27-0.58)	36	0.63 (0.47-0.80)
Age 6-12 years: hand		132	Kw	38	0.67 (0.44-0.90)	58	0.46 (0.27-0.65)	36	0.53 (0.32-0.74)
Body figure, height and weight									
**current**									
Body figure: male		105	Kw	30	0.75 (0.55-0.95)	48	0.59 (0.40-0.77)	27	0.69 (0.53-0.85)
Body figure: female		28	Kw	9	0.79 (0.61-0.96)	11	0.54 (0.06-1.00) †	8	0.74 (0.40-1.00)
Height, cm		130	ICC	40	0.99 (0.97-0.99)	60	0.95 (0.92-0.97)	30	0.87 (0.73-0.94)
Weight, kg		137	ICC	40	0.93 (0.76-0.97)	63	0.87 (0.66-0.94)	34	0.92 (0.83-0.96)
**10 years ago**									
Body figure: male		78	Kw	-	-	51	0.57 (0.39-0.75)	27	0.71 (0.54-0.88)
Body figure: female		19	Kw	-	-	11	0.45 (0.06-0.84) †	8	0.60 (0.32-0.87)
Weight, kg		96	ICC	-	-	62	0.23 (-0.02, 0.46)	34	0.65 (0.40-0.81)
**Age 19-30 years**									
Body figure: male		109	Kw	32	0.71 (0.53-0.88)	51	0.45 (0.21-0.69)	26	0.24 (-0.16, 0.63)
Body figure: female		28	Kw	9	0.73 (0.53-0.94)	11	0.77 (0.49-1.00)	8	0.78 (0.57-0.98)
**Age 13-18 years**									
Body figure: male		110	Kw	32	0.80 (0.68-0.92)	51	0.42 (0.17-0.67)	27	0.51 (0.16-0.87)
Body figure: female		28	Kw	9	0.54 (0.20-0.89)	11	0.66 (0.36-0.96)	8	0.75 (0.44-1.00)
**Age 6-12 years**									
Body figure: male		108	Kw	31	0.75 (0.55-0.94)	51	0.62 (0.43-0.81)	26	0.65 (0.42-0.89)
Body figure: female		28	Kw	9	0.90 (0.79-1.00)	11	0.49 (0.03-0.94) †	8	0.94 (0.88-1.00)
**FFQ at age 6-12 years**									
All meat	freq	137	ICC	40	0.49 (0.05-0.73) †	61	0.56 (0.25-0.74)	36	0.75 (0.50-0.87)
	port	131	ICC	37	0.59 (0.20-0.79)	63	0.42 (0.05-0.65) †	31	0.08 (-0.93, 0.56)
Animal liver	freq	138	ICC	40	0.77 (0.56-0.88)	63	0.60 (0.34-0.76)	35	0.72 (0.44-0.86)
	port	99	ICC	27	0.08 (-1.06, 0.59)	48	0.35 (-0.16, 0.63)	24	0.00 (-1.38, 0.57)

All fish	freq	138	ICC	40	0.61 (0.26-0.79)	62	0.67 (0.45-0.80)	36	0.73 (0.46-0.86)
	port	136	ICC	38	0.60 (0.23-0.79)	63	0.43 (0.07-0.65) †	35	0.28 (-0.43, 0.64)
All fruit	freq	138	ICC	40	0.70 (0.43-0.84)	62	0.72 (0.53-0.84)	36	0.75 (0.50-0.87)
	port	119	ICC	36	0.56 (0.15-0.77)	56	0.62 (0.35-0.78)	27	0.33 (-0.38, 0.68)
All vegetable	freq	139	ICC	40	0.70 (0.44-0.84)	63	0.68 (0.47-0.81)	36	0.86 (0.68-0.93)
	port	136	ICC	40	0.51 (0.06-0.74) †	61	0.64 (0.40-0.78)	35	0.66 (0.34-0.83)
Fresh milk	freq	139	ICC	40	0.65 (-0.00, 0.85)	63	0.58 (0.31-0.74)	36	0.52 (0.07-0.76) †
	port	70	ICC	30	0.70 (0.36-0.85)	38	0.54 (0.10-0.76)	2	1.00
Powdered milk	freq	138	ICC	40	0.32 (-0.30, 0.64)	63	0.51 (0.19-0.70)	35	0.67 (0.35-0.83)
	port	30	ICC	8	-0.62 (-46.7, 0.72)	19	0.66 (0.09-0.87) †	3	4.00 (4.00-4.00)
Soybean milk	freq	139	ICC	40	0.58 (0.20-0.78)	63	0.71 (0.52-0.83)	36	0.83 (0.67-0.91)
	port	82	ICC	30	0.32 (-0.45, 0.68)	39	0.55 (0.14-0.76)	13	0.60 (-0.42, 0.88)
Type of salted fish		140	K	41	0.42 (0.23-0.61)	63	0.17 (0.03-0.30) †	36	0.25 (0.07-0.44) †
Mouldy salted fish	freq	33	ICC	9	0.66 (-0.27, 0.92)	16	0.74 (0.23-0.91)	8	0.74 (-0.28, 0.95)
	port	33	ICC	9	0.80 (0.10-0.96)	17	0.91 (0.74-0.97)	7	0.83 (-0.01, 0.97)
Firmed salted fish	freq	29	ICC	5	0.63 (-0.55, 0.96)	16	0.59 (-0.17, 0.86)	8	0.97 (0.87-1.00)
	port	29	ICC	5	0.67 (-5.28, 0.97)	16	0.81 (0.44-0.93)	8	0.53 (-1.87, 0.91)
Other types of salted fish	freq	0	ICC	0	-	0	-	0	-
	port	0	ICC	0	-	0	-	0	-
Any types of salted fish	freq	9	ICC	3	-	3	0.92 (-0.24, 1.00)	3	0.98 (0.63-1.00)
	port	8	ICC	2	-	3	-	3	1.00
Preserved meat	freq	137	ICC	40	0.78 (0.59-0.89)	61	0.56 (0.27-0.74)	36	0.63 (0.27-0.81)
	port	110	ICC	33	0.57 (0.14-0.79)	53	0.59 (0.28-0.76)	24	0.95 (0.90-0.98)
Preserved egg	freq	139	ICC	41	0.40 (-0.13, 0.68)	62	0.77 (0.62-0.86)	36	0.63 (0.26-0.81)
	port	111	ICC	31	0.51 (-0.03, 0.77)	57	0.72 (0.52-0.83)	23	0.57 (-0.05, 0.82)
Preserved vegetable	freq	138	ICC	41	0.41 (-0.11, 0.69)	61	0.67 (0.45-0.80)	36	0.75 (0.51-0.87)
	port	98	ICC	27	0.30 (-0.59, 0.68)	47	0.60 (0.28-0.78)	24	0.63 (0.13-0.84)
Preserved fruit	freq	138	ICC	40	0.46 (0.17-0.67)	62	0.66 (0.50-0.78)	36	0.58 (0.31-0.76)

	port	81	ICC	26	0.21 (-0.77, 0.65)	45	0.74 (0.52-0.86)	10	0.36 (-0.63, 0.81)
**FFQ at age 13-18 years**									
Frequency of deep-fried food (Never/Less than once a month/Once a month/1-3 per week/4-6 per week/1-2 per day/3+ times per day)		140	Kw	41	0.49 (0.28-0.70)	63	0.26 (0.02-0.49) ⁺	36	0.21 (-0.21, 0.63)
Frequency of barbeque meat (Never/Less than once a month/Once a month/1-3 per week/4-6 per week/1-2 per day/3+ times per day)		140	Kw	41	0.60 (0.43-0.78)	63	0.38 (0.16-0.60)	36	0.45 (0.18-0.72)
Ever consumed burnt Chicken/duck/goose/pork skin (Yes/No)		140	K	41	0.24 (0.02-0.45) ⁺	63	0.29 (0.10-0.49)	36	0.33 (0.08-0.57) ⁺
Red meat	freq	139	ICC	40	0.38 (0.08-0.62) ⁺	63	0.52 (0.32-0.68)	36	0.43 (0.13-0.66)
	port	138	ICC	41	0.37 (0.07-0.61) ⁺	62	0.36 (0.12-0.56)	35	0.24 (-0.10, 0.53)
Poultry	freq	139	ICC	40	0.37 (0.07-0.61) ⁺	63	0.41 (0.18-0.59)	36	0.56 (0.29-0.75)
	port	131	ICC	41	0.45 (0.17-0.67)	60	0.44 (0.21-0.62)	30	0.24 (-0.13, 0.55)
Animal liver	freq	139	ICC	40	0.75 (0.57-0.86)	63	0.44 (0.22-0.62)	36	0.52 (0.24-0.72)
	port	102	ICC	29	0.65 (0.37-0.82)	50	0.24 (-0.04, 0.49)	23	0.63 (0.29-0.82)
Oily fish	freq	139	ICC	40	0.55 (0.29-0.73)	63	0.59 (0.40-0.73)	36	0.52 (0.23-0.72)
	port	79	ICC	32	0.68 (0.45-0.83)	37	0.35 (0.04-0.60) ⁺	10	0.43 (-0.27, 0.82)
Non-oily fish	freq	139	ICC	40	0.57 (0.31-0.75)	63	0.53 (0.31-0.69)	36	0.59 (0.32-0.77)
	port	115	ICC	33	0.30 (-0.05, 0.58)	55	0.14 (-0.13, 0.39)	27	0.36 (-0.02, 0.65)
Shellfish	freq	139	ICC	40	0.71 (0.51-0.83)	63	0.47 (0.25-0.64)	36	0.66 (0.43-0.81)
	port	114	ICC	34	0.58 (0.30-0.77)	54	0.30 (0.04-0.53) ⁺	26	0.21 (-0.17, 0.54)
Leafy green vegetable	freq	138	ICC	40	0.41 (0.11-0.64)	63	0.41 (0.19-0.59)	35	0.50 (0.21-0.71)
	port	137	ICC	41	0.35 (0.05-0.60) ⁺	62	0.55 (0.35-0.70)	34	0.31 (-0.03, 0.59)
Other vegetable	freq	140	ICC	41	0.70 (0.50-0.83)	63	0.34 (0.11-0.54)	36	0.15 (-0.19, 0.45)
	port	129	ICC	39	0.62 (0.39-0.78)	58	0.55 (0.34-0.71)	32	0.64 (0.39-0.81)
Carrot	freq	140	ICC	41	0.51 (0.23-0.70)	63	0.65 (0.47-0.77)	36	0.62 (0.38-0.79)
	port	108	ICC	38	0.52 (0.24-0.71)	52	0.44 (0.19-0.63)	18	0.18 (-0.31, 0.59)
Tomato	freq	139	ICC	41	0.49 (0.22-0.69)	63	0.50 (0.29-0.66)	35	0.59 (0.32-0.77)
	port	124	ICC	37	0.57 (0.31-0.76)	61	0.37 (0.13-0.57)	26	0.26 (-0.14, 0.59)

Citrus fruit	freq	139	ICC	40	0.55 (0.29-0.73)	63	0.45 (0.23-0.63)	36	0.63 (0.38-0.79)
	port	127	ICC	39	0.41 (0.12-0.64)	61	0.46 (0.24-0.64)	27	0.53 (0.19-0.75)
Other fruits	freq	140	ICC	41	0.47 (0.20-0.67)	63	0.38 (0.16-0.57)	36	0.68 (0.46-0.82)
	port	130	ICC	40	0.40 (0.11-0.63)	60	0.46 (0.23-0.64)	30	0.13 (-0.21, 0.46)
Fresh milk	freq	139	ICC	40	0.77 (0.61-0.87)	63	0.72 (0.57-0.82)	36	0.57 (0.31-0.76)
	port	79	ICC	33	0.63 (0.37-0.80)	40	0.44 (0.16-0.65)	6	0.62 (-0.18, 0.93)
Powdered milk	freq	139	ICC	40	0.55 (0.30-0.73)	63	0.36 (0.12-0.56)	36	0.44 (0.13-0.67)
	port	30	ICC	10	0.22 (-0.43, 0.72)	18	0.50 (0.07-0.78) ǂ	2	0.00 (-1.00, 1.00)
Dairy products	freq	140	ICC	41	0.53 (0.27-0.72)	63	0.71 (0.56-0.81)	36	0.45 (0.15-0.68)
	port	106	ICC	40	0.25 (-0.06, 0.51)	53	0.54 (0.32-0.70)	13	0.35 (-0.18, 0.74)
Eggs	freq	140	ICC	41	0.51 (0.25-0.70)	63	0.43 (0.21-0.61)	36	0.78 (0.61-0.88)
	port	132	ICC	39	0.42 (0.13-0.64)	62	0.11 (-0.13, 0.34)	31	0.42 (0.08-0.68) ǂ
Tofu	freq	140	ICC	41	0.17 (-0.15, 0.45)	63	0.69 (0.53-0.80)	36	0.75 (0.56-0.86)
	port	126	ICC	40	0.39 (0.09-0.63) ǂ	56	0.29 (0.03-0.51) ǂ	30	0.71 (0.48-0.85)
Soybean milk	freq	137	ICC	39	0.43 (0.14-0.66)	62	0.61 (0.43-0.74)	36	0.70 (0.49-0.84)
	port	96	ICC	34	0.41 (0.09-0.65) ǂ	44	0.50 (0.24-0.69)	18	0.41 (-0.01, 0.72)
Bean curd	freq	139	ICC	40	0.49 (0.21-0.69)	63	0.56 (0.36-0.71)	36	0.87 (0.75-0.93)
	port	112	ICC	37	0.43 (0.12-0.66)	54	0.22 (-0.04, 0.46)	21	0.62 (0.27-0.82)
Types of salted fish		139	K	40	0.44 (0.25-0.62)	63	0.15 (0.01-0.28) ǂ	36	0.40 (0.20-0.61)
Mouldy salted fish	freq	26	ICC	8	0.57 (-0.18, 0.90)	12	0.85 (0.56-0.95)	6	0.90 (0.49-0.99)
	port	26	ICC	8	0.63 (-0.08, 0.91)	12	0.66 (0.20-0.89)	6	0.80 (0.20-0.97)
Firmed salted fish	freq	36	ICC	5	0.60 (-0.31, 0.95)	20	0.68 (0.34-0.86)	11	0.60 (0.07-0.87) ǂ
	port	34	ICC	5	0.33 (-0.86, 0.91)	19	0.67 (0.33-0.86)	10	0.50 (-0.14, 0.85)
Other types of salted fish	freq	0	ICC	0	-	0	-	0	-
	port	0	ICC	0	-	0	-	0	-
Any types of salted fish	freq	13	ICC	6	0.93 (0.65-0.99)	4	0.84 (0.08-0.99) ǂ	3	0.96 (0.46-1.00)
	port	13	ICC	6	1.00 (1.00-1.00)	4	-0.50 (-1.23, 0.75)	3	-0.00 (-0.95, 0.95)
Preserved seafood	freq	139	ICC	41	0.50 (0.24-0.70)	63	0.59 (0.40-0.73)	35	0.60 (0.34-0.78)

	port	112	ICC	35	0.53 (0.25-0.73)	54	0.29 (0.03-0.51) †	23	0.61 (0.27-0.81)
Preserved vegetable	freq	139	ICC	41	0.67 (0.46-0.81)	63	0.54 (0.33-0.69)	35	0.56 (0.28-0.75)
	port	114	ICC	33	0.48 (0.18-0.70)	57	0.56 (0.35-0.71)	24	0.26 (-0.15, 0.60)
Preserved fruit	freq	140	ICC	41	0.52 (0.26-0.71)	63	0.60 (0.41-0.74)	36	0.71 (0.50-0.84)
	port	90	ICC	29	0.43 (0.10-0.68)	49	0.40 (0.14-0.61)	12	0.18 (-0.35, 0.65)
Preserved egg	freq	139	ICC	41	0.59 (0.35-0.76)	62	0.47 (0.25-0.64)	36	0.78 (0.61-0.88)
	port	112	ICC	30	0.36 (0.02-0.62) †	57	0.52 (0.30-0.69)	25	0.57 (0.22-0.78)
Preserved meat	freq	139	ICC	40	0.48 (0.20-0.69)	63	0.38 (0.12-0.57)	36	0.62 (0.37-0.79)
	port	113	ICC	36	0.39 (0.08-0.64) †	52	0.58 (0.37-0.73)	25	0.35 (-0.05, 0.65)
Processed meat	freq	140	ICC	41	0.69 (0.48-0.82)	63	0.64 (0.46-0.76)	36	0.33 (0.01-0.59) †
	port	100	ICC	35	0.26 (-0.09, 0.54)	52	0.37 (0.11-0.59)	13	0.84 (0.57-0.95)
Condiments	freq	139	ICC	41	0.38 (0.08-0.61) †	63	0.34 (0.10-0.54)	35	0.40 (0.08-0.65) †
	port	125	ICC	39	0.33 (0.01-0.58) †	62	0.27 (0.02-0.49) †	24	0.19 (-0.24, 0.55)
Green/white tea	freq	140	ICC	41	0.49 (0.22-0.69)	63	0.40 (0.17-0.59)	36	0.57 (0.31-0.75)
	port	77	ICC	28	0.27 (-0.12, 0.58)	41	0.32 (0.03-0.57) †	8	0.52 (-0.32, 0.88)
Oolong tea	freq	140	ICC	41	0.63 (0.40-0.78)	63	0.43 (0.21-0.61)	36	0.88 (0.77-0.94)
	port	70	ICC	30	-0.07 (-0.43, 0.30)	31	0.07 (-0.26, 0.39)	9	0.24 (-0.23, 0.72)
Red/black tea	freq	140	ICC	41	0.47 (0.20-0.68)	63	0.73 (0.59-0.83)	36	0.53 (0.25-0.73)
	port	88	ICC	28	0.18 (-0.21, 0.52)	47	0.21 (-0.08, 0.47)	13	0.57 (0.04- 0.85) †
Cantonese-style milk tea	freq	140	ICC	41	0.64 (0.41-0.79)	63	0.71 (0.56-0.81)	36	0.61 (0.36-0.78)
	port	70	ICC	24	-0.13 (-0.51, 0.29)	37	0.32 (0.00-0.58) †	9	1.00 (1.00-1.00)
Coffee	freq	139	ICC	40	0.80 (0.65-0.89)	63	0.62 (0.44-0.75)	36	0.18 (-0.16, 0.47)
	port	44	ICC	20	0.00 (-0.38, 0.41)	20	0.59 (0.22-0.82)	4	1.00
Chinese herbal tea	freq	139	ICC	40	0.60 (0.36-0.77)	63	0.52 (0.32-0.68)	36	0.74 (0.56-0.86)
	port	109	ICC	34	-0.02 (-0.36, 0.32)	55	0.53 (0.31-0.70)	20	0.46 (0.02-0.75) †
**FFQ at age 19-30 years**									
Any changes to your diet during 19-30 years old, compared with 13-18's (Yes/No)		135	K	38	0.56 (0.30-0.83)	62	0.33 (0.09-0.57) †	35	0.31 (-0.4, 0.66)
If yes, which food items									

All meat (yes/no)	77	K	26	0.24 (0.14-0.33)	30	0.20 (0.10-0.31)	21	0.18 (0.02-0.35) †
Animal liver (yes/no)	44	K	15	0.10 (-0.02, 0.22)	23	0.18 (0.07-0.29) †	6	0.12 (-0.07, 0.31)
Seafood (yes/no)	69	K	22	0.19 (0.08-0.29) †	28	0.15 (0.05-0.26) †	19	0.21 (0.05-0.37) †
Vegetable (yes/no)	63	K	18	0.15 (0.03-0.26) †	28	0.13 (0.02-0.23) †	17	0.19 (0.02-0.36) †
Fruit (yes/no)	72	K	21	0.17 (0.05-0.28) †	32	0.14 (0.04-0.25) †	19	0.22 (0.09-0.36) †
Dairy products (yes/no)	52	K	17	0.12 (0.01-0.24) †	25	0.14 (0.03-0.25) †	10	0.13 (-0.06, 0.31)
Egg (yes/no)	49	K	17	0.17 (0.04-0.29) †	19	0.14 (0.02-0.26) †	13	0.20 (0.02-0.37) †
Soybean products (yes/no)	55	K	19	0.17 (0.05-0.29) †	23	0.11 (-0.00, 0.22)	13	0.20 (0.02-0.37) †
Salted fish (yes/no)	49	K	17	0.13 (0.01-0.25) †	23	0.21 (0.10-0.32)	9	0.18 (0.02-0.34) †
Preserved food (yes/no)	50	K	16	0.14 (0.01-0.26) †	25	0.18 (0.07-0.28) †	9	0.17 (-0.02, 0.36)
Beverage	69	K	20	0.15 (0.03-0.26) †	28	0.18 (0.08-0.29) †	21	0.16 (0.01-0.31) †
**FFQ 10 years ago**								
Any changes to your diet before 10 years ago, compared with 19-30's	140	K	41		63	0.35 (0.10-0.60)	36	-0.08 (-0.40, 0.24)
If yes, which food items								
All meat (yes/no)	97	K	-	-	61	0.33 (0.14-0.52)	36	0.16 (-0.08, 0.41)
Animal liver (yes/no)	98	K	-	-	62	0.35 (0.17-0.54)	36	0.08 (-0.19, 0.35)
Seafood (yes/no)	95	K	-	-	61	0.33 (0.15-0.52)	34	-0.03 (-0.25, 0.20)
Vegetable (yes/no)	97	K	-	-	62	0.29 (0.10-0.47)	35	0.12 (-0.14, 0.37)
Fruit (yes/no)	98	K	-	-	62	0.29 (0.10-0.47)	36	0.12 (-0.12, 0.35)
Dairy products (yes/no)	96	K	-	-	61	0.41 (0.21-0.60)	35	0.07 (-0.17, 0.31)
Egg (yes/no)	96	K	-	-	61	0.24 (0.05-0.43) †	35	-0.00 (-0.25, 0.24)
Soybean products (yes/no)	97	K	-	-	62	0.29 (0.10-0.49)	35	0.12 (-0.14, 0.38)
Salted fish (yes/no)	94	K	-	-	59	0.32 (0.13-0.52)	35	0.06 (-0.20, 0.32)
Preserved food (yes/no)	97	K	-	-	62	0.29 (0.10-0.48)	35	0.03 (-0.24, 0.31)
Beverage	97	K	-	-	62	0.29 (0.11-0.48)	35	0.04 (-0.21, 0.30)

* only subjects who filled out the question in both questionnaires were included in the analysis.

ICC: intra-class correlation coefficient. K: Cohen's kappa. Kw: Weighted kappa. Freq: frequency (Never/Less than once a month/Once a month/1-3 per week/4-6 per week/1-2 per day/3+ per day). Port: portion (Small/Medium/Large). No differences by age groups at the first questionnaire (difference among coefficients >0.30, and tested by a Fisher Z transformation [p<0.01] with a Bonferonni correction) were found. (de Waal et al 2014). (Coefficients: 0-0.20 [poor]; 0.20-0.40 [fair]; 0.40-0.60 [moderate]; 0.60-0.80 [substantial]; 0.80-1.00 [almost perfect]). All the coefficients above were p<0.01, unless otherwise stated (†: 0.01≤p≤0.05); except the format of cell *(-x, x)* indicating p>0.05.
