# Supplementary material for: Test-retest reliability of a computer-assisted self-administered questionnaire on early life exposure in a nasopharyngeal carcinoma case-control study
Source: Sci Rep. 2018 May 4;8:7052. doi: 10.1038/s41598-018-25046-y (PMC5935670; doi:10.1038/s41598-018-25046-y)
Supplement: Supplementary file 2 — Supplementary materials 2 (questionnaires) [file 41598_2018_25046_MOESM2_ESM.pdf]

Supplementary materials 2 (questionnaires)

Test-retest reliability of a computer-assisted self-administered questionnaire on early life exposure in a nasopharyngeal carcinoma case-control study

Zhi-Ming MAI[1,2], Jia-Huang LIN[1,2], Shing-Chun CHIANG[1,2], Roger Kai-Cheong NGAN[2,5], Dora Lai-Wan KWONG[2,4], Wai-Tong NG[2,6], Alice Wan-Ying NG[2,7], Kam-Tong YUEN[2,8], Kai-Ming IP[1,2], Yap-Hang CHAN[2,3], Anne Wing-Mui LEE[2,4], Sai-Yin HO[1*], Maria Li LUNG[2,4] and Tai-Hing LAM[1,2]

[1] School of Public Health, The University of Hong Kong, Hong Kong SAR, China

[2] Centre for Nasopharyngeal Carcinoma Research (CNPCR), Research Grants Council Area of Excellence Scheme, The University of Hong Kong, Hong Kong SAR, China

[3] Department of Medicine, Queen Mary Hospital, The University of Hong Kong, Hong Kong SAR, China

[4] Department of Clinical Oncology, Queen Mary Hospital, The University of Hong Kong, Hong Kong SAR, China

[5] Department of Clinical Oncology, Queen Elizabeth Hospital, Hong Kong SAR, China

[6] Department of Clinical Oncology, Pamela Youde Nethersole Eastern Hospital, Hong Kong SAR, China

[7] Department of Clinical Oncology, Tuen Mun Hospital, Hong Kong SAR, China

[8] Department of Clinical Oncology, Princess Margaret Hospital, Hong Kong SAR, China

Content list

Chinese Questionnaire, p3
English Questionnaire, p38

鼻咽癌病例對照研究問卷
Version 20 (07 Aug 2017)

資料處理及保密

在任何情況下您的個人資料會嚴格保密。
只有研究人員才可查閱您的資料。
研究人員將採取所有合理可行措施確保收集的個人資料受到保護及合理使用。
此項研究的結果可能會在本地及國際醫學雜誌發表，數據只會以匿名方式發佈於學術界。
在任何情況下您的個人身份都不會被透露。

回答問卷時

請仔細閱讀及回答，所有問題都沒有所謂對或錯的答案，請盡量回憶並回答。
準確的答案對鼻咽癌研究非常重要，為鼻咽癌患者和預防鼻咽癌帶來幫助。
如果有任何疑問或不肯定，請詢問研究員。

目錄

第一部份：個人資料..6

第二部份：健康記錄..8

第三部分：生活習慣..16

第四部份：職業接觸..33

第五部分：問卷調查方式偏好研究..36

第六部分：調查員問卷質量評估..37

****此部分由調查員填寫****

z. 調查對象編號

z1. 訪問日期及時間:______日_____月________年________時________分

z2. 調查對象姓名: ______________________(中文)

______________________(英文)

z3. 參與者組別: □病例(請跳過z11 至z15) □對照(請跳過z9, z10, z16 至z19) z4.

年齡: ________

z5. 性別: □男 □女

z6. AoE/Epid 編號: __________________

z7. 聯絡電話: _______________________

z8. 現時居住於哪區:

香港島: □中西區 □東區 □南區 □灣仔區

九龍: □九龍城區 □觀塘區 □深水埗區 □黃大仙區 □油尖旺區

新界: □離島區 □葵青區 □北區 □西貢區 □沙田區 □大埔區
□荃灣區 □屯門區 □元朗區

其他: □澳門 □內地

z9. 鼻咽癌確診日期: 日 月____________年

z10. 調查對象是否第一次被診斷為鼻咽癌 (ICD-10,C11): □否 □是

z11. 門診/住院病人: □門診病人 □住院病人

z12. 專科門診部門: □內科 □骨科 □眼科 □外科 z13. 住院部

門: □內科 □骨科 □眼科 □外科 z14. 門

診轉介日期: 日 月__________________年

z15. 入院日期: 日 月____________年

z16. AoE consent status:

z17. 同意提供鼻腔組織: □否 □是 z18.

同意提供血液樣本: □否 □是 z19. 同意

提供唾液樣本: □否 □是 z20. Epi study

consent status:

z21. 同意問卷調查: □否 □是 □不合資格(Ineligible)

z22. 同意提供血液樣本: □否 □是

z23. 同意提供唾液樣本: □否 □是 □不適用 □AoE z24. (只限

QEH) 同意提供口腔抹片: □否 □是 □不適用 z25. 同意家長問

卷/聯繫: □否 □是

z26. 調查對象拒絕問卷調查的原因(可多選):

□在簽知情同意書或面談前已經離開醫院	□認為問卷調查會很花時間	□病情嚴重
□對本研究態度消極	□對醫院印象負面	□洩露個人隱私
□語言/ 聽力障礙	□視力障礙	□精神障礙
□沒有給出原因	□其他	

z27. 其他: ____

z28. 調查對象拒絕血液樣本的原因(可多選):

□在簽知情同意書或面談前已經離開醫院	□認為問卷調查會很花時間	□病情嚴重
□對本研究態度消極	□對醫院印象負面	□洩露個人隱私
□語言/ 聽力障礙	□視力障礙	□精神障礙
□沒有給出原因	□其他	

z29. 其他: ____

z30. 調查對象拒絕唾液樣本的原因(可多選):

□在簽知情同意書或面談前已經離開醫院	□認為問卷調查會很花時間	□病情嚴重
□對本研究態度消極	□對醫院印象負面	□洩露個人隱私
□語言/ 聽力障礙	□視力障礙	□精神障礙
□沒有給出原因	□其他	

z31. 其他: ____

z32. (只限 QEH)調查對象拒絕口腔抹片的原因(可多選):

□在簽知情同意書或面談前已經離開醫院 □認為問卷調查會很花時間 □病情嚴重
□對本研究態度消極 □對醫院印象負面 □洩露個人隱私
□語言/ 聽力障礙 □視力障礙 □精神障礙
□沒有給出原因 □其他

z33. 其他: ____

z34. 調查對象拒絕家長問卷/聯繫的原因(可多選):
□記憶問題 □已離世 □病情嚴重
□對本研究態度消極 □對醫院印象負面 □洩露個人隱私
□語言/ 聽力障礙 □視力障礙 □精神障礙
□沒有給出原因 □其他

z35. 其他: ____

z36. 血液樣本收集日期:______日_____月________年

z37. EDTA: □ 1 □ 2

z38. Clotted blood: □ 1 □ 2

z39. 唾液樣本樣本收集日期:______日_____月________年

z40. 唾液: □1 □2

z41. (只限QEH) 口腔抹片收集日期:_______日_____月________年

z42. Buccal Swab: □ 1 □2 □3

z43. 不合資格的調查對象

z44. 不合資格的原因:
□認知障礙症(痴呆症) □身體不適 □聽力問題
□不能填寫同意書 □不是一年內新症 □曾患有鼻咽癌
□懷孕 □其他

z45. 其他: ____

****此部分由調查對象填寫****

第一部份：個人資料

首先是關於您與家族的一些基本資料，請選擇符合您情況的答案。

b1a. 請問您有多少個親生兄弟姐妹(包括在世與已過世的，不包括您自己)?
(請注意並不包括收養的和同父異母或同母異父的兄弟姐妹)
b1b.______哥哥 b1c.______姐姐 b1d.______弟弟 b1e.______妹妹 b1f. 不適用

b2a. 您的婚姻狀況是?
□單身(指從未結婚) □已婚 □離婚/分居 □配偶已去世

b3a. 您有多少個親生子女? (包括在世與已過世的)
b3b.______兒子 b3c.______女兒 b3d. 不適用

b4a. 您的家鄉方言/母語是? (可多選)
□蜑（蛋）家話 □潮州話 □客家話 □閩南話 □粵語
□廣州/廣府話 □台 ft 話 □中 ft 話 □東莞話 □梧州話
□其他粵語方言 □湖南話 □普通話 □廣西方言 □其他

b4b. 您親生母親的家鄉方言/母語是? (可多選)
□蜑（蛋）家話 □潮州話 □客家話 □閩南話 □粵語
□廣州/廣府話 □台 ft 話 □中 ft 話 □東莞話 □梧州話
□其他粵語方言 □湖南話 □普通話 □廣西方言 □其他

b5a. 您的出生地點是?
□香港(跳至b5b) □澳門(跳至b5b) □廣西(跳至b5b)
□廣東(跳至b6a) □福建(跳至b7a) □湖南(跳至b5b)
□中國其他省份(跳至b5b) □其他國家(跳至b8a) □不知道(跳至b5b)

b5b.您親生母親的出生地點是?
□香港(跳至b12a) □澳門(跳至b12a) □廣西(跳至b12a)
□廣東(跳至b9a) □福建(跳至b10a) □湖南(跳至b12a)
□中國其他省份(跳至b12a) □其他國家(跳至b11a) □不知道(跳至b12a)

b6a. 您的出生地點是廣東的哪裡?
□廣州 □深圳(寶安) □中 ft □珠海 □佛 ft □茂名 □肇慶 □惠州 □湛江
□江門 □河源 □韶關 □東莞 □汕尾 □陽江 □梅州 □清遠 □雲浮 □潮州
□汕頭 □揭陽 □不知道 (跳至b9a/b10a/b11a/b12a)

b7a. 您的出生地點是福建的哪裡?
□福州 □莆田 □三明 □南平 □龍巖 □寧德 □漳州 □泉州 □廈門
□不知道(跳至b9a/b10a/b11a/b12a)

b8a. 您的出生地點是其他國家中的哪裡？

□馬來西亞 □泰國 □其他國家

b9a. 您親生母親的出生地點是廣東的哪裡？

□廣州 □深圳(寶安) □中 ft □珠海 □佛 ft □茂名 □肇慶 □惠州 □湛江
□江門 □河源 □韶關 □東莞 □汕尾 □陽江 □梅州 □清遠 □雲浮 □潮州
□汕頭 □揭陽 □不知道 (跳至b12a)

b10a. 您親生母親的出生地點是福建的哪裡？

□福州 □莆田 □三明 □南平 □龍巖 □寧德 □漳州 □泉州 □廈門
□不知道(跳至b12a)

b11a. 您親生母親的出生地點是其他國家中的哪裡？

□馬來西亞 □泰國 □其他國家

b12a. 您在出生地居住到多少歲？(只回答其中一項)________歲

b12b. 或哪一年？ ____年

b13a. 在您十歲左右，您所居住的住所是什麼類型的？

□臨時房屋/籠屋 □公屋 □居屋 □私人樓 (業主) □私人樓(租住)
□船上 □木屋 □石屋 □鄉村屋 □其他 □不清楚

b14a. 您的最高教育程度是？

□未受正式教育 □私塾(卜卜齋) □小學 □中一至中三 □中四至中五
□中六或預科 □職業及其他進修學院 □大學及以上 □不清楚

b14b. 您親生父親的最高教育程度是？

□未受正式教育 □私塾(卜卜齋) □小學 □中一至中三 □中四至中五
□中六或預科 □職業及其他進修學院 □大學及以上 □不清楚

b14c. 您親生母親的最高教育程度是？

□未受正式教育 □私塾(卜卜齋) □小學 □中一至中三 □中四至中五
□中六或預科 □職業及其他進修學院 □大學及以上 □不清楚

第二部份：健康記錄

現在我們想了解您的個人患病記錄。

c1b. (除此次患病外) 請問您曾經患有癌症嗎？ □沒有 □有

c1c. 如果有，請選擇(可多選)

□肺癌 □肝癌 □胃癌 □結直腸癌 □食管癌 □血癌(白血病)
□胰腺癌 □腦瘤 □膀胱癌 □非霍奇金淋巴癌 □乳腺癌(女士填寫)
□子宮頸癌(女士填寫) □子宮癌(女士填寫) □卵巢癌(女士填寫) □前列腺癌(男士填寫)
□其他:___________________________

(病史一) 若曾經患有上述癌症，跳至以下問題 (若曾患有多種癌症請相應地回答下列問題)：

a. 患有_________(癌症) 的確診時間？(只回答其中一項)
b. 年齡：_______歲；
c. □不清楚
d. 或大概確診年齡段：

□0-4 歲 □5-9 歲 □10-14 歲 □15-19 歲 □20-24 歲 □25-29 歲
□30-34 歲 □35-39 歲 □40-44 歲 □45-49 歲 □50-54 歲 □55-59 歲
□60-64 歲 □65-69 歲 □70-74 歲 □75-79 歲 □80-84 歲 □85 歲或以上
□不清楚

e. 您曾經因此接受過化療嗎？ □沒有 □有
f. 您曾經因此接受過放射治療嗎？ □沒有 □有

(病史二) 若曾經患有上述癌症，跳至以下問題 (若曾患有多種癌症請相應地回答下列問題)：

a. 患有_________(癌症) 的確診時間？(只回答其中一項)
b. 年齡：_______歲；
c. 不清楚
d. 或大概確診年齡段：

□0-4 歲 □5-9 歲 □10-14 歲 □15-19 歲 □20-24 歲 □25-29 歲
□30-34 歲 □35-39 歲 □40-44 歲 □45-49 歲 □50-54 歲 □55-59 歲
□60-64 歲 □65-69 歲 □70-74 歲 □75-79 歲 □80-84 歲 □85 歲或以上
□不清楚

e. 您曾經因此接受過化療嗎？ □沒有 □有
f. 您曾經因此接受過放射治療嗎？ □沒有 □有

(病史三) 若曾經患有上述癌症，跳至以下問題 (若曾患有多種癌症請相應地回答下列問題)：

a. 患有_________(癌症) 的確診時間？(只回答其中一項)
b. 年齡：_______歲；
c. 不清楚
d. 或大概確診年齡段：

□0-4 歲 □5-9 歲 □10-14 歲 □15-19 歲 □20-24 歲 □25-29 歲
□30-34 歲 □35-39 歲 □40-44 歲 □45-49 歲 □50-54 歲 □55-59 歲
□60-64 歲 □65-69 歲 □70-74 歲 □75-79 歲 □80-84 歲 □85 歲或以上

□不清楚

e. 您曾經因此接受過化療嗎？ □沒有 □有

f. 您曾經因此接受過放射治療嗎？ □沒有 □有

c19a. 您曾經接受過外科手術嗎？ □沒有 □有
c19b. 如果有，請註明. (只回答其中一項)
c19c. 接受外科手術年齡:________歲
c19d. 或哪一年:_______年
c19e. 接受手術原因: □癌症 □創傷或骨科 □其他

c20a. (確診前) 醫生曾否告訴您患有以下非傳染性疾病嗎？ □沒有 □有
c20b. 如果有，請選擇(可多選)

□高血壓 □糖尿病 □中風 □心臟病 □慢性鼻竇炎 □鼻息肉 □塵肺(矽肺/石棉肺)
□胃/十二指潰瘍病 □長期鼻塞 □過敏性鼻炎(鼻敏感) □胃酸反流(倒流)症
□慢性阻塞性肺病 □高血脂(高膽固醇/高脂血症) □骨質疏鬆症 □囊性纖維症
□克隆氏症(克罗恩病，局限性腸炎) □脂肪肝

c20c. (確診前) 醫生曾否告訴您患有以下傳染性疾病？ □沒有 □有
c20d. 如果有，請選擇(可多選)

□傳染性單核細胞增多症(接吻病，腺熱) □肺炎 □中耳炎 □肝炎(跳至c21a)
□性傳染病 □生殖器疣(例如：尖銳濕疣) □愛滋病或愛滋病病毒感染

c21a. 是哪種類型肝炎?

□甲型肝炎 □乙型肝炎 □丙型肝炎 □不清楚

d1. 嬰兒期

d1a. 以下是關於您親生母親在懷有您時和您的嬰兒期餵養情況

d1b. 您的親生母親在懷有您期間是否有經歷過任何疾病？

□沒有(跳至d1d) □有 □不清楚(跳至d1d)

d1c. 如果是，請註明

□貧血 □高血壓 □糖尿病 □其他

d1d. 您母親在分娩您期間是否有經歷過任何意外狀況？

□沒有(跳至d2a) □有 □不清楚(跳至d2a)

d1e. 如果是，請註明

□胎位不正 □胎膜早破 □臍帶纏脖 □其他

d2a. 您母親分娩您時：

□自然產(跳至d2c) □剖腹產 □不清楚(跳至d2c)

d2b. 您是否緊急剖腹產？

□計劃剖腹產 □緊急剖腹產 □不清楚

d2c. 您是否單胎或多胞胎?

□單胎 □多胞胎 □不清楚

d2e. 您母親分娩您時，您是：

□早產______週(d2f)

□足月

□比預產期遲

□不清楚(跳至d4a)

d4a. 您出生時體重:________公斤; d4b. □不清楚

d5a. 請問您在嬰兒期是由母乳餵哺嗎？

□每次都是餵母乳，沒有用奶粉

□開始時是餵母乳，其後改用奶粉

□從出生開始到斷奶期，都是部分時間餵母乳，部分時間餵奶粉

□沒有餵母乳，全部時間都是餵奶粉

□不清楚(跳至e1a)

d5b. 您由母乳餵養總共有多少個月？ ________月 d5c. □不清楚

e1a. 家族癌症病歷記錄

我們希望了解您的家族癌症病史，請注意家庭成員指的是您和具有血緣關係的親戚，包括在世的和已經過世的親戚。請注意並不包括收養的和繼父母，繼子女，同父異母或同母異父的兄弟姐妹。請盡量回憶並提供可能多的信息，例如，您不能回憶家族成員確診癌症時的具體年齡，但您記得是40多歲，請回答40 多歲，但是如果您沒有任何記憶，請回答"不清楚"。

e1b. 以下家族成員曾經患有癌症嗎(不包括癌症復發和轉移)? □沒有 □有

e1c. 如果有，請選擇(可多選)

□母親 □父親 □兄弟 □姊妹 □祖父 □祖母
□外祖父 □外祖母 □女兒 □兒子 □其他(跳至e158aa)

以上任何一個家族成員曾經患有癌症時跳至以下問題，若同一個家人曾患有多種癌症，則每一種癌症需要重複一次。

(家庭成員一)

a. 您的(家庭成員)________曾經患有的癌症(不包括癌症復發和轉移) 是：(可多選)

□鼻咽癌 □肺癌 □肝癌 □胃癌 □結直腸癌 □食管癌 □血癌(白血病)
□胰腺癌 □腦瘤 □膀胱癌 □非霍奇金淋巴癌 □乳腺癌(女士填寫)
□子宮頸癌(女士填寫) □子宮癌(女士填寫) □卵巢癌(女士填寫) □前列腺癌(男士填寫)
□其他：__________________________________

a. 您的(家庭成員)患有上述__________(癌症)的確診時間：

b. 確診年齡：________歲；

c. □不清楚

d. 或確診時的大概年齡段：

□0-4 歲 □5-9 歲 □10-14 歲 □15-19 歲 □20-24 歲 □25-29 歲
□30-34 歲 □35-39 歲 □40-44 歲 □45-49 歲 □50-54 歲 □55-59 歲
□60-64 歲 □65-69 歲 □70-74 歲 □75-79 歲 □80-84 歲 □85 歲或以上
□不清楚

(家庭成員二)

a. 您的(家庭成員)________曾經患有的癌症(不包括癌症復發和轉移) 是：(可多選)

□鼻咽癌 □肺癌 □肝癌 □胃癌 □結直腸癌 □食管癌 □血癌(白血病)
□胰腺癌 □腦瘤 □膀胱癌 □非霍奇金淋巴癌 □乳腺癌(女士填寫)
□子宮頸癌(女士填寫) □子宮癌(女士填寫) □卵巢癌(女士填寫) □前列腺癌(男士填寫)
□其他：__________________________________

a. 您的(家庭成員)患有上述__________(癌症)的確診時間：

b. 確診年齡：________歲；

c. □不清楚

d. 或確診時的大概年齡段：

□0-4 歲 □5-9 歲 □10-14 歲 □15-19 歲 □20-24 歲 □25-29 歲
□30-34 歲 □35-39 歲 □40-44 歲 □45-49 歲 □50-54 歲 □55-59 歲
□60-64 歲 □65-69 歲 □70-74 歲 □75-79 歲 □80-84 歲 □85 歲或以上

□不清楚

e158aa. 您共有幾個其他家族成員曾患癌症?

□1 個　□2 個　□3 個　□4 個　□5 個

e158a. 您與曾經患有癌症的其他家族成員(1)的關係是：

□伯父　□叔父　□姑媽　□姑姐　□堂兄弟姊妹

□舅父　□姨媽　□細姨　□表兄弟姊妹

e158b. 您與曾經患有癌症的其他家族成員(2)的關係是：

□伯父　□叔父　□姑媽　□姑姐　□堂兄弟姊妹

□舅父　□姨媽　□細姨　□表兄弟姊妹

e158c. 您與曾經患有癌症的其他家族成員(3)的關係是：

□伯父　□叔父　□姑媽　□姑姐　□堂兄弟姊妹

□舅父　□姨媽　□細姨　□表兄弟姊妹

e158d. 您與曾經患有癌症的其他家族成員(4)的關係是：

□伯父　□叔父　□姑媽　□姑姐　□堂兄弟姊妹

□舅父　□姨媽　□細姨　□表兄弟姊妹

e158e. 您與曾經患有癌症的其他家族成員(5)的關係是：

□伯父　□叔父　□姑媽　□姑姐　□堂兄弟姊妹

□舅父　□姨媽　□細姨　□表兄弟姊妹

(其他家庭成員一)

aa. (其他家族成員)________共患有幾次癌症? □一次　□兩次　□三次

a. 您的(其他家庭成員)________第一次患有的癌症(不包括癌症復發和轉移) 是：

□鼻咽癌　□肺癌　□肝癌　□胃癌　□結直腸癌　□食管癌　□血癌(白血病)

□胰腺癌　□腦瘤　□膀胱癌 □非霍奇金淋巴癌

□乳腺癌(女士填寫)　□子宮頸癌(女士填寫)　□子宮癌(女士填寫)　□卵巢癌(女士填寫)

□其他：(b) ________________________________

c. 您的(其他家庭成員) 第一次患癌的確診年齡：________歲；

d. □不清楚

e. 或確診時的大概年齡段：

□0-4 歲　□5-9 歲　□10-14 歲　□15-19 歲　□20-24 歲　□25-29 歲

□30-34 歲　□35-39 歲　□40-44 歲　□45-49 歲　□50-54 歲　□55-59 歲

□60-64 歲　□65-69 歲　□70-74 歲　□75-79 歲　□80-84 歲　□85 歲或以上

□不清楚

f. 您的(其他家庭成員)________第二次患有的癌症(不包括癌症復發和轉移) 是：

□鼻咽癌　□肺癌　□肝癌　□胃癌　□結直腸癌　□食管癌　□血癌(白血病)

□胰腺癌　□腦瘤　□膀胱癌 □非霍奇金淋巴癌

□乳腺癌(女士填寫)　□子宮頸癌(女士填寫)　□子宮癌(女士填寫)　□卵巢癌(女士填寫)

□其他：(g) ________________________________

h. 您的(其他家庭成員) 第一次患癌的確診年齡：________歲；
i. □不清楚
j. 或確診時的大概年齡段：

□0-4 歲 □5-9 歲 □10-14 歲 □15-19 歲 □20-24 歲 □25-29 歲
□30-34 歲 □35-39 歲 □40-44 歲 □45-49 歲 □50-54 歲 □55-59 歲
□60-64 歲 □65-69 歲 □70-74 歲 □75-79 歲 □80-84 歲 □85 歲或以上
□不清楚

k. 您的(其他家庭成員)________第三次患有的癌症(不包括癌症復發和轉移) 是：
□鼻咽癌 □肺癌 □肝癌 □胃癌 □結直腸癌 □食管癌 □血癌(白血病)
□胰腺癌 □腦瘤 □膀胱癌 □非霍奇金淋巴癌
□乳腺癌(女士填寫) □子宮頸癌(女士填寫) □子宮癌(女士填寫) □卵巢癌(女士填寫)
□其他：(l) ________________________________
m. 您的(其他家庭成員) 第一次患癌的確診年齡：________歲；
n. □不清楚
o. 或確診時的大概年齡段：

□0-4 歲 □5-9 歲 □10-14 歲 □15-19 歲 □20-24 歲 □25-29 歲
□30-34 歲 □35-39 歲 □40-44 歲 □45-49 歲 □50-54 歲 □55-59 歲
□60-64 歲 □65-69 歲 □70-74 歲 □75-79 歲 □80-84 歲 □85 歲或以上
□不清楚

(其他家庭成員二)

aa. (其他家族成員)________共患有幾次癌症? □一次 □兩次 □三次
a. 您的(其他家庭成員)________第一次患有的癌症(不包括癌症復發和轉移) 是：
□鼻咽癌 □肺癌 □肝癌 □胃癌 □結直腸癌 □食管癌 □血癌(白血病)
□胰腺癌 □腦瘤 □膀胱癌 □非霍奇金淋巴癌
□乳腺癌(女士填寫) □子宮頸癌(女士填寫) □子宮癌(女士填寫) □卵巢癌(女士填寫)
□其他：(b) ________________________________
c. 您的(其他家庭成員) 第一次患癌的確診年齡：________歲；
d. □不清楚
e. 或確診時的大概年齡段：

□0-4 歲 □5-9 歲 □10-14 歲 □15-19 歲 □20-24 歲 □25-29 歲
□30-34 歲 □35-39 歲 □40-44 歲 □45-49 歲 □50-54 歲 □55-59 歲
□60-64 歲 □65-69 歲 □70-74 歲 □75-79 歲 □80-84 歲 □85 歲或以上
□不清楚

f. 您的(其他家庭成員)________第二次患有的癌症(不包括癌症復發和轉移) 是：
□鼻咽癌 □肺癌 □肝癌 □胃癌 □結直腸癌 □食管癌 □血癌(白血病)
□胰腺癌 □腦瘤 □膀胱癌 □非霍奇金淋巴癌
□乳腺癌(女士填寫) □子宮頸癌(女士填寫) □子宮癌(女士填寫) □卵巢癌(女士填寫)
□其他：(g) ________________________________
h. 您的(其他家庭成員) 第一次患癌的確診年齡：________歲；
i. □不清楚
j. 或確診時的大概年齡段：

□0-4 歲 □5-9 歲 □10-14 歲 □15-19 歲 □20-24 歲 □25-29 歲
□30-34 歲 □35-39 歲 □40-44 歲 □45-49 歲 □50-54 歲 □55-59 歲
□60-64 歲 □65-69 歲 □70-74 歲 □75-79 歲 □80-84 歲 □85 歲或以上
□不清楚

k. 您的(其他家庭成員)________第三次患有的癌症(不包括癌症復發和轉移) 是：
□鼻咽癌 □肺癌 □肝癌 □胃癌 □結直腸癌 □食管癌 □血癌(白血病)
□胰腺癌 □腦瘤 □膀胱癌 □非霍奇金淋巴癌
□乳腺癌(女士填寫) □子宮頸癌(女士填寫) □子宮癌(女士填寫) □卵巢癌(女士填寫)
□其他：(l) ________________________________
m. 您的(其他家庭成員) 第一次患癌的確診年齡：________歲；
n. □不清楚
o. 或確診時的大概年齡段：
□0-4 歲 □5-9 歲 □10-14 歲 □15-19 歲 □20-24 歲 □25-29 歲
□30-34 歲 □35-39 歲 □40-44 歲 □45-49 歲 □50-54 歲 □55-59 歲
□60-64 歲 □65-69 歲 □70-74 歲 □75-79 歲 □80-84 歲 □85 歲或以上
□不清楚

第三部分：生活習慣

以下問題是關於您在不同人生階段的飲食習慣，包括童年(6 歲至12 歲), 青少年(13 歲至18 歲), 成年(19 歲至30 歲)及10 年前。

為幫助您記起飲食頻率和份量，請參考照片中食物的份量。請以您該時期平均飲食量作答。這些資料非常重要，請盡量準確回答。如果任何不清楚之處，請詢問調查員。

fa1a. 人生階段: 6 歲至12 歲的飲食習慣

fa2c. 請問您的飲食習慣是否曾經受過文化大革命的影響？ □沒有 □有

	食物類別	人生階段1(6 歲至12 歲)									
		從來沒吃過	少於每月1 至2 次	每月1 至2 次	每週1-3 次	每週4-6 次	每日1-2 次	每日3 次或以上	平均每次吃的份量		
									細	中	大
fa3h	肉類食物(牛，豬，羊,雞，鴨，鵝等)(碗)										
fa4l	肝臟(豬潤，雞肝，鴨肝，鵝肝等) (碗)										
fa5h	魚(碗)										
fa6h	水果(碗)										
fa7h	蔬菜(碗)										
fa7a	鮮奶(杯)										
fa8a	奶粉(杯)										
fa9h	豆漿(杯)										

fa10a. 您吃哪種類的鹹魚? (梅香鹹魚肉質鬆軟，鹹中帶香，有濃烈而獨特的香味。實肉鹹魚肉質結實，成片，咸而鮮。若您不能分辨哪種鹹魚，請選擇所有鹹魚種類)

□沒有 □梅香鹹魚 □實肉鹹魚 □梅香及實肉鹹魚 □其他鹹魚

□所有鹹魚種類(包括鹹魚雞粒炒飯) □不清楚

	食物類別	人生階段1(6 歲至12 歲)									
		從來沒吃過	少於每月1 至2 次	每月1 至2 次	每週1-3 次	每週4-6 次	每日1-2 次	每日3 次或以上	平均每次吃的份量		
									細	中	大
fa11h	梅香鹹魚(湯匙)										
fa12h	實肉鹹魚(湯匙)										
fa13h	其他鹹魚(湯匙)										
fa14h	所有鹹魚種類(包括鹹魚雞粒炒飯, 魚香茄子飯, 鹹魚蒸肉餅, 鹹魚頭豆腐										

	湯，鹹魚仔等) (湯匙)										
fa15h	中式醃製的肉類(臘肉，臘鴨，臘腸，潤腸等)(碗)										
fa16h	經處理蛋類製品 (鹹蛋，皮蛋等) (隻)										
fa17h	醃製蔬菜(鹹酸菜，梅菜，酸蕎頭，菜脯，榨菜，雪菜，冬菜，醃白蘿蔔等)(湯匙)										
fa18h	醃製水果(話梅，杏脯，甘草欖，陳皮，嘉應子，芒果乾，八仙果，提子乾等) (碗)										

fb1a. 人生階段: 13 歲至18 歲的飲食習慣

	食物類別	人生階段2（13 歲到18 歲）						
		從來沒吃過	少於每月 1 至2 次	每月 1 至2 次	每週 1-3 次	每週 4-6 次	每日 1-2 次	每日3 次或以上
fb1b	煎炸食物(油器, 煎魚，煎豬扒，炸春卷，炸雞翼)							
fb1c	燒烤食物(叉燒，燒肉，燒雞/鴨/鵝)							

fb1d. 您有幾經常食用燒烤食物時, 會食用半燒焦或燒焦的部分嗎？ □沒有 □有

	食物類別	人生階段2（13 歲到18 歲）									
		從來沒吃過	少於每月1 至2 次	每月1 至2 次	每週1-3 次	每週4-6 次	每日1-2 次	每日3 次或以上	平均每次吃的份量		
									細	中	大
fb2h	肉類食物(牛，豬，羊等) (碗)										
fb3h	家禽(雞，鴨，鵝等) (碗)										
fb4h	肝臟(豬潤，雞肝，鴨肝，鵝肝等) (碗)										
fb5h	脂質魚(三文魚，鯖魚，吞拿魚，沙丁魚，鱒魚等) (碗)										
fb6h	非脂質魚(老虎斑，大眼雞，牙帶，鯉魚，桂花魚等) (碗)										
fb7h	甲殼，貝殼類(碗)										
fb8h	綠葉蔬菜(菠菜，生菜，芥蘭，菜心，白菜，通菜，椰菜，西蘭花等) (碗)										

fb9h	非綠葉蔬菜(青瓜，絲瓜，黃瓜等) (碗)										
fb10h	紅蘿蔔(碗)										
fb11h	番茄(碗)										
fb12h	柑橘類水果(橙，柑，柚，橘等) (碗)										
fb13h	其他水果(碗)										
fb14h	鮮奶(杯)										
fb15h	奶粉(杯)										
fb16h	其他奶類製品(雪糕，酸奶或芝士) (杯)										
fb17h	蛋(包括蛋黃) (隻)										
fb18h	豆腐(碗)										
fb19h	豆漿(杯)										
fb20h	豆卜(碗)										

fb21h. 您吃哪種類的鹹魚? (梅香鹹魚肉質鬆軟，鹹中帶香，有濃烈而獨特的香味。實肉鹹魚肉質結實，成片，咸而鮮。若您不能分辨哪種鹹魚，請選擇所有鹹魚種類)

□没有 □梅香鹹魚 □實肉鹹魚 □梅香及實肉鹹魚 □其他鹹魚

□所有鹹魚種類(包括鹹魚雞粒炒飯) □不清楚

	食物類別	人生階段2（13 歲到18 歲）									
		從來沒吃過	少於每月1 至2 次	每月1 至2 次	每週1-3次	每週4-6次	每日1-2次	每日3次或以上	平均每次吃的份量		
									細	中	大
fb22h	梅香鹹魚(湯匙)										
fb23h	實肉鹹魚(湯匙)										
fb24h	其他鹹魚(湯匙)										
fb25h	所有鹹魚種類(包括鹹魚雞粒炒飯，魚香茄子飯，鹹魚蒸肉餅，鹹魚頭豆腐湯，鹹魚仔等) (湯匙)										
fb26h	其他乾製海產(魷魚，蝦乾，瑤柱，蠔豉，海參，蝦米等) (湯匙)										
fb27h	醃製蔬菜(鹹酸菜，梅菜，酸蕎頭，菜脯，榨菜，雪菜，冬菜，醃白蘿蔔等) (湯匙)										
fb28h	醃製水果(話梅，杏脯，甘草欖，陳皮，										

	嘉應子，芒果乾，八仙果,提子乾等) (碗)									
fb29h	經處理蛋類製品(鹹蛋，皮蛋等) (隻)									
fb30h	中式醃製的肉類(臘肉，臘鴨，臘腸，潤腸等) (碗)									
fb31h	其他煙熏及經處理的肉製品(火腿，腸仔，餐肉等) (碗)									
fb32h	經發酵調味料(蝦醬，蟹醬，豉油，豆豉，腐乳) (湯匙)									
fb33h	綠/白茶類(茉莉花茶，龍井，壽眉) (杯)									
fb34h	烏龍茶類(鐵觀音，水仙等) (杯)									
fb35h	紅/黑茶類(磚茶，普洱等) (杯)									
fb36h	奶茶(杯)									
fb37h	咖啡(杯)									
fb38h	其他中國草藥飲品(涼茶，盒仔茶，中藥，廿四味，五花茶等) (碗)									

fc1aa. 人生階段: 19 歲至30 歲的飲食習慣

fc1a. 您在19 歲至30 歲時的飲食習慣與13 歲至18 歲時比較有沒有變化？ ▫沒有 ▫有

fc2a. 您在19 歲至30 歲時攝入肉類及家禽類食物習慣與13 歲至18 歲時有沒有變化？ ▫沒有 ▫有

	食物類別	人生階段3（19 歲到30 歲）								
		從來沒吃過	少於每月1 至2 次	每月1 至2 次	每週1-3次	每週4-6次	每日1-2次	每日3次或以上	平均每次吃的份量	
									細	中	大
fc3h	肉類食物(牛，豬，羊等) (碗)										
fc4h	家禽(雞，鴨，鵝等) (碗)										
fc5h	肝臟(豬潤，雞肝，鴨肝，鵝肝等) (碗)										

fc6a. 您在19 歲至30 歲時攝入魚，甲殼，殼類食物習慣與13 歲至18 歲時有沒有變化？▫沒有 ▫有

	食物類別	人生階段3（19 歲到30 歲）									
		從來沒吃過	少於每月1 至2 次	每月1 至2 次	每週1-3次	每週4-6次	每日1-2次	每日3次或以上	平均每次吃的份量		
									細	中	大

fc7h	脂質魚(三文魚，鯖魚，吞拿魚，沙丁魚，鱒魚等) (碗)										
fc8h	非脂質魚(老虎斑，大眼雞，牙帶，鯉魚，桂花魚等) (碗)										
fc9h	甲殼, 貝殼類(碗)										

fc10a. 您在19 歲至30 歲時攝入蔬菜類食物習慣與13 歲至18 歲時有沒有變化？ □沒有 □有

	食物類別	人生階段3（19 歲到30 歲）									
		從來沒吃過	少於每月1 至2 次	每月1 至2 次	每週1-3 次	每週4-6 次	每日1-2 次	每日3 次或以上	平均每次吃的份量		
									細	中	大
fc11h	綠葉蔬菜(菠菜，生菜，芥蘭，菜心，白菜，通菜，椰菜，西蘭花等) (碗)										
fc12h	非綠葉蔬菜(青瓜，絲瓜，黃瓜等) (碗)										
fc13h	紅蘿蔔(碗)										
fc14h	番茄(碗)										

fc15a. 您在19 歲至30 歲時攝入水果類食物習慣與13 歲至18 歲時比較有沒有變化？ □沒有 □有

	食物類別	人生階段3（19 歲到30 歲）									
		從來沒吃過	少於每月1 至2 次	每月1 至2 次	每週1-3 次	每週4-6 次	每日1-2 次	每日3 次或以上	平均每次吃的份量		
									細	中	大
fc16h	柑橘類水果(橙，柑，柚，橘等) (碗)										
fc17h	其他水果(碗)										

fc18a. 您在19 歲至30 歲時攝入奶製品或蛋習慣與13 歲至18 歲時比較有沒有變化？ □沒有 □有

	食物類別	人生階段3（19 歲到30 歲）									
		從來沒吃過	少於每月1 至2 次	每月1 至2 次	每週1-3 次	每週4-6 次	每日1-2 次	每日3 次或以上	平均每次吃的份量		
									細	中	大
fc19h	鮮奶(杯)										
fc20h	奶粉(杯)										
fc21h	其他奶類製品(雪糕，酸奶或芝士) (杯)										
fc22h	蛋(包括蛋黃) (隻)										

fc23a. 您在19 歲至30 歲時攝入豆製品類食物習慣與13 歲至18 歲時比較有沒有變化？□沒有 □有

	食物類別	人生階段3（19 歲到30 歲）								
		從來沒吃過	少於每月1 至2 次	每月1 至2 次	每週1-3 次	每週4-6 次	每日1-2 次	每日3 次或以上	平均每次吃的份量		
									細	中	大
fc24h	豆腐(碗)										
fc25h	豆漿(杯)										
fc26h	豆卜(碗)										

fc27a. 您在19 歲至30 歲時攝入鹹魚類食物習慣與13 歲至18 歲時比較有沒有變化？ ▫沒有 ▫有

	食物類別	人生階段3（19 歲到30 歲）								
		從來沒吃過	少於每月1 至2 次	每月1 至2 次	每週1-3 次	每週4-6 次	每日1-2 次	每日3 次或以上	平均每次吃的份量		
									細	中	大
fc28h	梅香鹹魚(湯匙)										
fc29h	實肉鹹魚(湯匙)										
fc30h	其他鹹魚(湯匙)										
fc31h	所有鹹魚種類(包括鹹魚雞粒炒飯, 魚香茄子飯, 鹹魚蒸肉餅, 鹹魚頭豆腐湯，鹹魚仔等) (湯匙)										

fc32a. 您在19 歲至30 歲時攝入其他醃製類食物習慣與13 歲至18 歲時比較有沒有變化？
▫沒有 ▫有

	食物類別	人生階段3（19 歲到30 歲）								
		從來沒吃過	少於每月1 至2 次	每月1 至2 次	每週1-3 次	每週4-6 次	每日1-2 次	每日3 次或以上	平均每次吃的份量		
									細	中	大
fc33h	其他乾製海產(魷魚，蝦乾，瑤柱，蠔豉，海參，蝦米等) (湯匙)										
fc34h	醃製蔬菜(鹹酸菜，梅菜，酸蕎頭，菜脯，榨菜，雪菜，冬菜，醃白蘿蔔等) (湯匙)										
fc35h	醃製水果(話梅，杏脯，甘草欖，陳皮，嘉應子，芒果乾，八仙果,提子乾等) (碗)										
fc36h	經處理蛋類製品(鹹蛋，皮蛋等) (隻)										

fc37h	中式醃製的肉類(臘肉，臘鴨，臘腸，潤腸等) (碗)										
fc38h	其他煙熏及經處理的肉製品(火腿，腸仔，餐肉等) (碗)										
fc39h	經發酵調味料(蝦醬，蟹醬，豉油，豆豉，腐乳等) (湯匙)										

fc40a.您在19 歲至30 歲時飲用茶或其他飲料習慣與13 歲至18 歲時比較有沒有變化？ □沒有 □有

	食物類別	人生階段3（19 歲到30 歲）									
		從來沒吃過	少於每月1 至2 次	每月1 至2 次	每週1-3次	每週4-6次	每日1-2次	每日3次或以上	平均每次吃的份量		
									細	中	大
fc41h	綠/白茶類(茉莉花茶，龍井，壽眉等) (杯)										
fc42h	烏龍茶類(鐵觀音，水仙等) (杯)										
fc43h	紅/黑茶類(磚茶，普洱等) (杯)										
fc44h	奶茶(杯)										
fc45h	咖啡(杯)										
fc46h	其他中國草藥飲品(涼茶，盒仔茶,中藥，廿四味，五花茶) (碗)										

fd1aa. 人生階段: 10 年前

fd1a. 您在10 年前的飲食習慣與19 歲至30 歲時比較有沒有變化？ □沒有 □有

fd2a. 您在10 年前時攝入肉類及家禽類食物習慣與19 歲至30 歲時有沒有變化？ □沒有 □有

	食物類別	人生階段4（10 年前）									
		從來沒吃過	少於每月1 至2 次	每月1 至2 次	每週1-3次	每週4-6次	每日1-2次	每日3次或以上	平均每次吃的份量		
									細	中	大
fd3h	肉類食物(牛，豬，羊等) (碗)										
fd4h	家禽(雞，鴨，鵝等) (碗)										
fd5h	肝臟(豬潤，雞肝，鴨肝，鵝肝等) (碗)										

fd6a. 您在10 年前時攝入魚，甲殼，貝殼類食物習慣與19 歲至30 歲時有沒有變化？ □沒有 □有

	食物類別	人生階段4（10 年前）									
		從來沒吃過	少於每月1 至2 次	每月1 至2 次	每週1-3 次	每週4-6 次	每日1-2 次	每日3 次或以上	平均每次吃的份量		
									細	中	大
fd7h	脂質魚(三文魚，鯖魚，吞拿魚，沙丁魚，鱒魚等) (碗)										
fd8h	非脂質魚(老虎斑，大眼雞，牙帶，鯉魚，桂花魚等) (碗)										
fd9h	甲殼，貝殼類(碗)										

fd10a. 您在10 年前時攝入蔬菜類食物習慣與19 歲至30 歲時有沒有變化？ □沒有 □有

	食物類別	人生階段4（10 年前）									
		從來沒吃過	少於每月1 至2 次	每月1 至2 次	每週1-3 次	每週4-6 次	每日1-2 次	每日3 次或以上	平均每次吃的份量		
									細	中	大
fd11h	綠葉蔬菜(菠菜，生菜，芥蘭，菜心，白菜，通菜，椰菜，西蘭花等) (碗)										
fd12h	非綠葉蔬菜(青瓜，絲瓜，黃瓜等) (碗)										
fd13h	紅蘿蔔(碗)										
fd14h	番茄(碗)										

fd15a. 您在10 年前時攝入水果類食物習慣與19 歲至30 歲時有沒有變化？ □沒有 □有

	食物類別	人生階段4（10 年前）									
		從來沒吃過	少於每月1 至2 次	每月1 至2 次	每週1-3 次	每週4-6 次	每日1-2 次	每日3 次或以上	平均每次吃的份量		
									細	中	大
fd16h	柑橘類水果(橙，柑，柚，橘等)(碗)										
fd17h	其他水果(碗)										

fd18a. 您在10 年前時攝入奶製品或蛋習慣與19 歲至30 歲時有沒有變化？ □沒有 □有

	食物類別	人生階段4（10 年前）									
		從來沒吃過	少於每月1 至2 次	每月1 至2 次	每週1-3 次	每週4-6 次	每日1-2 次	每日3 次或以上	平均每次吃的份量		
									細	中	大
fd19h	鮮奶(杯)										
fd20h	奶粉(杯)										
fd21h	其他奶類製品(雪糕，酸奶或芝士) (杯)										

fd22h	蛋(包括蛋黃) (隻)										

fd23a. 您在10 年前攝入豆製品類食物習慣與19 歲至30 歲時有沒有變化？ □沒有 □有

	食物類別	人生階段4（10 年前）									
		從來沒吃過	少於每月1 至2 次	每月1 至2 次	每週1-3次	每週4-6次	每日1-2次	每日3次或以上	平均每次吃的份量		
									細	中	大
fd24h	豆腐(碗)										
fd25h	豆漿(杯)										
fd26h	豆卜(碗)										

fd27a. 您在10 年前時攝入鹹魚類食物習慣與19 歲至30 歲時比較有沒有變化？ □沒有 □有

	食物類別	人生階段4（10 年前）									
		從來沒吃過	少於每月1 至2 次	每月1 至2 次	每週1-3次	每週4-6次	每日1-2次	每日3次或以上	平均每次吃的份量		
									細	中	大
fd28h	梅香鹹魚(湯匙)										
fd29h	實肉鹹魚(湯匙)										
fd30h	其他鹹魚(湯匙)										
fd31h	所有鹹魚種類(包括鹹魚雞粒炒飯，魚香茄子飯，鹹魚蒸肉餅，鹹魚頭豆腐湯，鹹魚仔等) (湯匙)										

fd32a. 您在10 年前時攝入其他醃製類食物習慣與19 歲至30 歲時比較有沒有變化？ □沒有 □有

	食物類別	人生階段4（10 年前）									
		從來沒吃過	少於每月1 至2 次	每月1 至2 次	每週1-3次	每週4-6次	每日1-2次	每日3次或以上	平均每次吃的份量		
									細	中	大
fd33h	其他乾製海產(魷魚，蝦乾，瑤柱，蠔豉，海參，蝦米等) (湯匙)										
fd34h	醃製蔬菜(鹹酸菜，梅菜，酸蕎頭，菜脯，榨菜，雪菜，冬菜，醃白蘿蔔等) (湯匙)										
fd35h	醃製水果(話梅，杏脯，甘草欖，陳皮，嘉應子，芒果乾，八仙果,提子乾等) (碗)										
fd36h	經處理蛋**類**製品(鹹										

	蛋，皮蛋等) (隻)										
fd37h	中式醃製的肉類(臘肉，臘鴨，臘腸，潤腸等) (碗)										
fd38h	其他煙熏及經處理的肉製品(火腿，腸仔，餐肉等) (碗)										
fd39h	經發酵調味料(蝦醬，蟹醬，豉油，豆豉，腐乳) (湯匙)										

fd40a. 您在10 年前時飲用茶或其他飲料習慣與19 歲至30 歲時比較有沒有變化？ □沒有 □有

	食物類別	人生階段4（10 年前）									
		從來沒吃過	少於每月1 至2 次	每月1 至2 次	每週1-3 次	每週4-6 次	每日1-2 次	每日3 次或以上	平均每次吃的份量		
									細	中	大
fd41h	綠/白茶類(茉莉花茶，龍井，壽眉等) (杯)										
fd42h	烏龍茶類(鐵觀音，水仙等) (杯)										
fd43h	紅/黑茶類(磚茶，普洱等) (杯)										
fd44h	奶茶(杯)										
fd45h	咖啡(杯)										
fd46h	其他中國草藥飲品(涼茶，盒仔茶,中藥，廿四味，五花茶) (碗)										

fe1a. 您是否曾經進食魚油/魚肝油/ omega-3 深海魚油? □否(跳至fe2a) □是
fe1b. 從幾歲開始進食魚油/魚肝油/ omega-3 深海魚油? _____歲
fe1c. 到幾歲? _____歲
fe1d. 進食魚油/魚肝油/ omega-3 深海魚油(1 粒/茶匙)的頻率是
□少於每月1 至2 次 □每月1 至2 次 □每週1-3 次 □每週4-6 次
□每天1-2 次 □每日3 次或以上

fe2a. 請問您有沒有進食維他命補充劑？(可多選) : □沒有(跳至g1a) □有
fe2b. 如果有，請選擇(可多選)
□綜合維生素補充劑 □維他命A 補充劑 □維他命B 補充劑
□維他命C 補充劑 □維他命D 補充劑 □維他命E 補充劑

fe3a. 從幾歲開始進食綜合維生素補充劑? _____歲

fe3b. 到幾歲? _____歲

fe3c. 進食綜合維生素補充劑(粒)的頻率是

□少於每月1 至2 次 □每月1 至2 次 □每週1-3 次 □每週4-6 次
□每天1-2 次 □每日3 次或以上

fe4a. 從幾歲開始進食維他命A 補充劑? _____歲

fe4b. 到幾歲? _____歲

fe4c. 進食維他命A 補充劑(粒)的頻率是

□少於每月1 至2 次 □每月1 至2 次 □每週1-3 次 □每週4-6 次
□每天1-2 次 □每日3 次或以上

fe5a. 從幾歲開始進食維他命B 補充劑? _____歲

fe5b. 到幾歲? _____歲

fe5c. 進食維他命B 補充劑(粒)的頻率是

□少於每月1 至2 次 □每月1 至2 次 □每週1-3 次 □每週4-6 次
□每天1-2 次 □每日3 次或以上

fe6a. 從幾歲開始進食維他命C 補充劑? _____歲

fe6b. 到幾歲? _____歲

fe6c. 進食維他命C 補充劑(粒)的頻率是

□少於每月1 至2 次 □每月1 至2 次 □每週1-3 次 □每週4-6 次
□每天1-2 次 □每日3 次或以上

fe7a. 從幾歲開始進食維他命D 補充劑? _____歲

fe7b. 到幾歲? _____歲

fe7c. 進食維他命D 補充劑(粒)的頻率是

□少於每月1 至2 次 □每月1 至2 次 □每週1-3 次 □每週4-6 次
□每天1-2 次 □每日3 次或以上

fe8a. 從幾歲開始進食維他命E 補充劑? _____歲

fe8b. 到幾歲? _____歲

fe8c. 進食維他命E 補充劑(粒)的頻率是

□少於每月1 至2 次 □每月1 至2 次 □每週1-3 次 □每週4-6 次
□每天1-2 次 □每日3 次或以上

g1a. 體型比例
在以下這個部分，我們希望了解您成長過程中的體型變化，請在合適的體型圖上選擇。

g1b. 請問以下哪一個畫像最符合您現在的體型？

g3a. 請問您現時的身高是? ______厘米(1 厘米=0.39 寸)
g3b. 請問您現時的體重是? ______公斤(1 公斤=2.2 磅)

g4a/g5a. 以下哪一個畫像最符合您10 年前的體型比例？

g6a. 請問您10 年前的體重是？ ______公斤(1 公斤=2.2 磅)

g7a/g8a. 以下哪一個畫像最符合您在19 歲至30 歲的體型比例？

g9a/g10a. 以下哪一個畫像最符合您在13 歲至18 歲的體型比例？

g11a/g12a. 以下哪一個畫像最符合您在6 歲至12 歲的體型比例？

h1a. 口腔衛生

h1b. 以下是關於您的口腔衛生的問題。

h1c. 蛀牙的數目是？

□沒有(跳至h1e)　□1-2　□3-4　□5 或以上

h1d. 您曾因為蛀牙而失去牙齒的數目是？

□沒有　□1-2　□3-4　□5 或以上

h1e. 您曾經患有牙周病嗎？ □沒有(跳至i1b)　□有

h1f. 您曾因為牙周病而失去牙齒的數目是？

□沒有　□1-2　□3-4　□5 或以上

i1a. 吸煙習慣

我們希望能夠了解您吸煙或接觸二手煙的情況。

i1b. 您曾經吸煙嗎？(連續6 個月每天至少吸1 支煙)

□從不吸煙(跳至i4)　□現在吸煙　□過去有吸煙習慣

i1c. 現在或戒煙前，您早上醒後幾耐會吸第一支煙？

□少於5 分鐘　□6-30 分鐘　□31-60 分鐘　□多於60 分鐘

i1d. 您從幾歲開始吸煙？ _____歲

i2b. 您幾歲戒煙？ _____歲

i2c. 您戒煙多久了？ _____年(如少於一年請填“0”)

i3b. 您在青少年(18 歲之前)平均每日吸幾多支煙？ ____支

i3c. 您在成年(18 歲之後) 平均每日吸幾多支煙？ _____支

i4. 二手煙

童年及青少年時期(6-18 歲)

i4a. 在18 歲之前，與您同住的人中有多少人吸煙？

□沒有(跳至i8b)　□一人　□二人　□三人

(同住吸煙人 1)

c. 同住吸煙人與您的關係是？

□父親 □母親 □祖父 □祖母 □外祖父 □外祖母 □兄弟姐妹 □其他

d. 每天接觸與您同住吸煙人多少分鐘？

□少於5 分鐘 □30-60 分鐘 □1-2 小時 □多於2 小時

e. 18 歲之前您與以上吸煙者同住多少年？ ______年

(同住吸煙人 2)

c. 同住吸煙人與您的關係是？

□父親 □母親 □祖父 □祖母 □外祖父 □外祖母 □兄弟姐妹 □其他

d. 每天接觸與您同住吸煙人多少分鐘？

□少於5 分鐘 □30-60 分鐘 □1-2 小時 □多於2 小時

e. 18 歲之前您與以上吸煙者同住多少年？ ______年

成年時期(18 歲後)

i8b. 在18 歲之後，與您同住的人中有多少人吸煙？

□沒有(跳至i12a) □一人 □二人 □三人

(同住吸煙人 1)

c. 同住吸煙人與您的關係是？

□父親 □母親 □祖父 □祖母 □外祖父 □外祖母 □兄弟姐妹 □其他

d. 每天接觸與您同住吸煙人多少分鐘？

□少於5 分鐘 □30-60 分鐘 □1-2 小時 □多於2 小時

e. 18 歲之後您與以上吸煙者同住多少年？ ______年

(同住吸煙人 2)

c. 同住吸煙人與您的關係是？

□父親 □母親 □祖父 □祖母 □外祖父 □外祖母 □兄弟姐妹 □其他

d. 每天接觸與您同住吸煙人多少分鐘？

□少於5 分鐘 □30-60 分鐘 □1-2 小時 □多於2 小時

e. 18 歲之後您與以上吸煙者同住多少年？ ______年

i12a. 我們希望能夠了解您的喝酒情況。

i12b. 您有沒有喝含酒精的飲料，如啤酒，葡萄酒，烈酒等？(至少每月一次，連續6 個月)

□完全沒飲或僅在特別場合(一年1-2 次) (跳至j1b) □現在有 □過去有

i12c. 您目前至少每月一次喝酒嗎？ □否 □有

i12d. 您幾歲開始喝酒? (至少每月一次)_

______________________歲 i13b. 您幾歲戒酒? _

______________________歲
i13c. 或您戒酒多少年?_______年
i14b. 您喝哪種類型的酒精飲料? (可多選)
□紅葡萄酒 □白葡萄酒 □啤酒 □烈酒(茅台，白蘭地，威士忌等)

	酒精飲料類別	平均每次吃的份量(杯)							
		從來沒吃過	少於每月1 次	每月 1 次	每週 1-3 次	每週 4-6 次	每日 1-2 次	每日3 次或以上	平均每次飲用的份量
i11h	紅葡萄酒(杯)<br>(中杯:100ml)								杯
i16aa	白葡萄酒(杯)<br>(中杯:100ml)								杯
i17b	啤酒(杯)<br>(中杯:100ml)								杯
i17c	啤酒(罐)<br>(中:375ml)								罐
i17e	啤酒(瓶)<br>(中:330ml)								瓶
i18h	烈酒(茅台，白蘭地，威士忌等) (杯) (小杯:10ml) (中杯:30ml)								杯

j.體育運動與陽光照射

b. 請問您有多經常進行戶外活動？（例如散步，去公園海灘，爬 ft，踢球，外出購物）
□少於每月1 至2 次 □每月1 至2 次 □每週1-3 次 □每週4-6 次 □每日

c. 平均每天在陽光下度過多少小時？
□少於1 小時 □2-4 小時 □5-7 小時 □8-10 小時

d. 您會避免陽光照射嗎？
□從來不會 □稍微會 □適度避免 □盡力去避免

e. 在陽光猛烈時，您會使用防曬措施嗎？（例如戴帽/穿長袖衣/撐傘/塗防曬霜）
□從來不會 □稍微會 □適度避免 □盡力去避免

j5a. 請根據圖片中1-4 的皮膚顏色，對照自己的皮膚顏色選擇您認為合適的號碼。

您覺得哪個號碼最能代表您的膚色？
j5b. 現時臉部膚色: □1 □2 □3 □4
j5c. 現時手背膚色: □1 □2 □3 □4
j5d. 10 年前手背膚色: □1 □2 □3 □4

j5e. 19 歲至30 歲手背膚色: □1 □2 □3 □4

j5f. 13-18 歲手背膚色: □1 □2 □3 □4

j5g. 6-12 歲手背膚色: □1 □2 □3 □4

第四部份：職業接觸

k1b. 請問在您的所有工作中有沒有接觸/吸入灰塵，化學氣體，燃燒產生的煙霧，酸/鹼?
□沒有(跳至k6b) □有 □不適用(跳至k6b)

k1c. 如果有，請選擇(可多選):
□灰塵(跳至k2c) □化學氣體(跳至k3c) □燃燒產生的煙霧(跳至k4c) □酸/鹼(跳至k5c)

灰塵

k2c. 請問您工作中有沒有吸入下列灰塵？(可多選)
□木塵 □金屬塵 □紡織塵 □皮革塵 □石棉塵 □水泥塵
□粉筆塵 □煤塵 □土塵 □香塵 □其他
k2d. 在接觸灰塵時，您的工作是屬於以下哪一個行業? ________________________
k2e. 與上面灰塵接觸的工作職位名稱是？ ________________________
k2f. 灰塵接觸有多少年？ ________年
k2g. 請說明您接觸上述灰塵的程度？
□輕微(少許感覺難受) □中等 □嚴重(感覺很難受)

化學氣體

k3c. 請問您工作中有沒有吸入下列化學氣體？(可多選)
□木頭防腐劑 □甲醛 □有機溶劑 □殺蟲劑/農藥 □苯類 □染料 □油料 □其他
k3d. 在接觸化學氣體時，您的工作是屬於以下哪一個行業? ________________________
k3e. 與上面吸入化學氣體的工作職位名稱是？ ________________________
k3f. 接觸化學氣體有多少年?________年
k3g. 請說明您接觸上述化學氣體的程度？
□輕微(少許感覺刺鼻) □中等 □嚴重(感覺很刺鼻)

燃燒產生的煙霧

k4c. 請問您工作中有沒有吸入下列燃燒產生的煙霧嗎？(可多選)
□柴油 □汽油 □煤 □木柴 □焦油 □天然氣 □汽車廢氣 □焊接煙氣
□其他
k4d. 在接觸燃燒產生的煙霧時，您的工作是屬於以下哪一個行業? ________________________
k4e. 與上面燃燒產生的煙霧接觸的工作職位名稱是？ ________________________
k4f. 接觸燃燒產生的煙霧有多少年?________年
k4g. 請說明您接觸上述燃燒產生的煙霧的程度？
□輕微(少許感覺刺鼻) □中等 □嚴重(感覺很刺鼻)

酸/鹼

k5c. 請問您工作中有沒有暴露於下列酸/鹼嗎？(可多選)
□鉻酸 □硫酸 □鹽酸 □硝酸 □濃鹼 □氨氣 □其他
k5d. 在接觸酸/鹼時，您的工作是屬於以下哪一個行業? ________________________
k5e. 與上面接觸於酸/鹼的工作職位名稱是？ ________________________
k5f. 接觸酸/鹼有多少年?________年

k5g. 請說明您接觸上述酸/鹼的程度？
□輕微(少許感覺刺鼻) □中等 □嚴重(感覺很刺鼻)

pc10.二手煙- 工作場所

k6b. 18 歲之前, 您的工作場所有沒有人抽煙? □沒有(跳至k7b) □有 □不適用(跳至k7b)
k6c. 平均每個工作天暴露於二手煙的時間有多久?
□少於5 分鐘 □30-60 分鐘 □1-2 小時 □多於2 小時
k6d. 於有人吸煙的場所工作多少年?_______年

k7b. 18 歲之後, 您的工作場所有沒有人抽煙? □沒有(跳至l1b) □有 □不適用(跳至l1b)
k7c. 平均每個工作天暴露於二手煙的時間有多久?
□少於5 分鐘 □30-60 分鐘 □1-2 小時 □多於2 小時
k7d. 於有人吸煙的場所工作多少年?_______年

l1a.現在我們想了解您住所的情況

b.______歲住所
c. 住所使用的燃料類型? （可多選）
□煤氣 □火水 □柴 □電 □煤 □生物質 □石油氣 □其他 □不清楚
d. 住所使用的油類型? （可多選）
□調和油 □動物油 □芥花子油 □花生油 □大豆油 □橄欖油
□其他 □不清楚
e. 住所幾經常炒餸?
□沒有 □每月 □每星期1-3 次 □每星期4-6 次 □每日1 次 □每日2-3 次
f. 居住住所最主要的水源是?
□管道系統(市政供水) □水井 □河水或運河水 □不清楚
g. 住所幾經常會接觸蚊香？
□每日 □每星期 □每月 □很少 □沒有
h. 住所幾經常會點香?
□每日 □逢初一/十五 □偶爾（逢年過節） □沒有

m1a. 工作收入

m1b. (確診前) 您的就業狀況是?
□自僱 □受僱 □退休 □家庭主婦 □學生 □待業

m1c. (確診前) 您的個人每月平均收入是(HK$)?
□沒有收入 □少於$15,000 □$15,000-24,999 □$25,000-39,999 □$40,000 或以上

m1d. (確診前) 您每月總家庭總平均收入是(HK$)?
□沒有收入 □少於$15,000 □$15,000-24,999 □$25,000-39,999 □$40,000 或以上

第五部分：問卷調查方式偏好研究

z1a. 你有否使用過台式電腦、筆記本電腦、智能手機或平板電腦？
□未用過　□間中使用（偶然）　□經常使用（每天）

z1b. 你會傾向選擇使用什麼工具回答調查問卷？
□紙張和筆　□平板電腦　□沒有意見

z1c. 你會傾向選擇由誰回答調查問卷？
□自己　□調查員　□調查員和自己　□沒有意見

z1d. 你對使用平板電腦回答調查問卷的態度？
□非常不喜歡　□不喜歡　□沒有意見　□喜歡　□非常喜歡

z1e. 你選擇平板電腦回答調查問卷的原因是？（可多選）
□便於展示　□內容清晰　□易於使用：容易回答　□較短的填表時間
□私密性好　□數據安全　□能簡單和舒適地掌控

z1f. 你在使用時有否遇到任何困難/問題？

____

____

z1g. 任何意見或改進建議（如上）：

____

____

問卷調查結束

我們的問卷調查到此為止。我們非常感謝您的參與。

請調查員記錄禮券簽收收據。調查員和調查對象簽署一份禮券簽收收據。

調查員向調查對象提供超市禮券。

第六部分：調查員問卷質量評估

o1b/o1c. 以下哪一個畫像最符合調查對象的體型比例？

在以下這個部分，調查員將可以看到一張膚色量度圖畫，請根據圖片中1-4 的膚色，並對照調查對象的膚色選擇。

o1d. 調查對象現時臉部膚色: □1 □2 □3 □4
o1e. 調查對象現時手背膚色: □1 □2 □3 □4

o2a. 以下問題想知道主要由<調查對象/調查員/其他人>閱讀問卷。

o2e. 第1-4 部份(家族基本資料，病人健康記錄，母親懷孕及餵養，家族癌症病史):
□調查對象 □調查員 □其他
o2ea. 第5 部份(飲食頻率問卷- 6 歲至12 歲): □調查對象 □調查員 □其他 o2eb.
第5 部份(飲食頻率問卷- 13 歲至18 歲): □調查對象 □調查員 □其他
o2ec. 第5 部份(飲食頻率問卷- 19 歲至30 歲): □調查對象 □調查員 □其他
o2ed. 第5 部份(飲食頻率問卷- 10 年前): □調查對象 □調查員 □其他
o2ee. 第6-9 部份(體型比例, 口腔衛生, 吸煙, 喝酒): □調查對象 □調查員 □其他
o2ef. 第10-13 部份(陽光照射, 職業接觸, 住所情況, 工作收入): □調查對象 □調查員 □其他

o2f. 主要應答人：
□調查對象 □調查對象父母 □調查對象子女 □調查對象兄弟姐妹
□其他 □調查員

o2fa. 主要填表人：(可多選)
□調查對象 □調查對象父母 □調查對象子女 □調查對象兄弟姐妹
□其他 □調查員

o2g. 調查對象回答問題的可靠性: □非常不可靠 □未必可靠 □可靠 □非常可靠

o2ga. 非常不可靠/不可靠的原因: ______________________________

o2gb. 其他: ______________________________

o2h. 請寫下您對這次訪問的任何意見或補充資料

____

____

o2i. 請記錄您可能對面試有任何意見

____

____

調查員簽名: ____________

Nasopharyngeal Carcinoma Case-Control Study Questionnaire
Version 14 (13.08.2017)

Privacy Statement

- All data will be kept strictly confidential and anonymous.
- Your personal information will only be accessible to relevant researchers.
- All applicable measures will be taken to ensure the security of collected data and personal information.
- The results may be published in local and/or international journals without disclosure of any participant's identity.
- Your personal identity will not be disclosed in any situation.

Instructions

- Please read and answer each question carefully. There are no right or wrong answers. Please try your best to recall your habits and answer the questions correspondingly.
- Your answers are very important to nasopharygneal carcinoma patients and the prevention of nasopharygneal carcinoma.
- Please refer to the Interviewer if you have any questions.

Contents

PART 1： Demographics 43

PART 2： Health Record 45

PART 3： Lifestyle 51

PART 4： Occupational Exposures 69

PART 5： Survey preference format 71

PART 6： Interviewer assessment 72

****To be filled in by interviewer****

z. Subject Identity

z1. Recruitment Date and Time: _____________(dd/mm/yyyy) ____________ (hh/mm)

z2. Subject Name: _____________________ (Chinese)
_____________________ (English)

z3. Subject Group: □Case (Please skip z11 to z15) □Control (Please skip z.9, z.10, z.18 to z.21)

z4. Age: _____

z5. Sex: □Male □Female

z6. AoE/Epid Number： ________________

z7. Contact Number: _____________________

z8. Current Residential District:

Hong Kong Island: □Central and Western District □ Eastern District
□ Southern District □ Wan Chai District

Kowloon: □Kowloon City District □Kwun Tong District □Sham Shui Po District
□Wong Tai Sin District □Yau Tsim Mong District

New Territories: □Islands District □Kwai Tsing District □North District
□Sai Kung District □Sha Tin District □Tai Po District
□Tsuen Wan District □Tuen Mun District □Yuen Long District

Others: □Macau □Mainland

z9. Date of NPC Diagnosis: _____________(dd/mm/yyyy)

z10. Is this the first NPC diagnosis for the subject (ICD-10, C11)? □No □Yes

z11. Out/in-patient: □Outpatient □Inpatient

z12. Specialist Outpatient Clinic: □Medicine □Orthopedics □Ophthalmology □Surgery

z13. Inpatient Department: □Medicine □Orthopedics □Ophthalmology □Surgery

z14. Outpatient Referral Date: _____________(dd/mm/yyyy)

z15. Inpatient Referral Date: _____________(dd/mm/yyyy)

z16. AoE Consent Status:

z17. Consent to biopsy: □No □Yes

z18. Consent to blood sample: □No □Yes

z19. Consent to saliva sample: □No □Yes

z20. Epi study Consent Status:

z21. Consent to Questionnaire: □No □Yes □Ineligible

z22. Consent to Blood Sample: □No □Yes

z23. Consent to Saliva Sample: □No □Yes □N/A □AoE

z24 (QEH only) Consent to Buccal Swabs: □No □Yes □N/A

z25. Consent to Parent Questionnaire: □No □Yes

z26. Reasons for Questionnaire Refusal (Multiple responses allowed):
□Discharge from hospital before giving consent to questionnaire □Questionnaire takes too much time
□Severe illness □Negative response to questionnaire □Negative impression on hospital
□Confidentiality concerms □Language/hearing impairment □Visual impairment
□Mental impairment □No reasons □Others

z27. Others: ______________________________________

z28. Reasons for Blood Collection Refusal (Multiple responses allowed):
□Discharge from hospital before giving consent to questionnaire □Questionnaire takes too much time
□Severe illness □Negative response to questionnaire □Negative impression on hospital
□Confidentiality concerms □Language/hearing impairment □Visual impairment
□Mental impairment □No reasons □Others

z29. Others: ______________________________________

z30. Reasons for Saliva Collection Refusal (Multiple responses allowed):
□Discharge from hospital before giving consent to questionnaire □Questionnaire takes too much time
□Severe illness □Negative response to questionnaire □Negative impression on hospital
□Confidentiality concerms □Language/hearing impairment □Visual impairment
□Mental impairment □No reasons □Others

z31. Others: ______________________________________

z32. (QEH only) Reasons for Buccal Swab Refusal (Multiple responses allowed):
□Discharge from hospital before giving consent to questionnaire □Questionnaire takes too much time
□Severe illness □Negative response to questionnaire □Negative impression on hospital
□Confidentiality concerms □Language/hearing impairment □Visual impairment
□Mental impairment □No reasons □Others

z33. Others: ______________________________________

z34. Reasons for Parent Consent/Contact Refusal (Multiple responses allowed):
□Memory problem □Deceased □Severe illness □Negative response to questionnaire
□Negative impression on hospital □Confidentiality concerms □Language/hearing impairment
□Visual impairment □Mental impairment □No reasons □Others

z35. Others: ______________________________________

z36. Blood Sample Collection Date: _____________(dd/mm/yyyy)

z37. EDTA: □1 □2

z38. Clotted Blood: □1 □2

z39. Saliva Sample Collection Date: _____________(dd/mm/yyyy)

z40. Saliva: □1 □2

z41. (QEH only) Buccal Swabs Sample Collection Date: _____________(dd/mm/yyyy)

z42. Buccal Swab: □1 □2 □3

z43. Ineligible Subject

z44. Reasons for ineligibility:
□Dementia □Severe illness □Hearing problem □Unable to give consent
□Diagnosis not within 1 year □History of NPC □Pregnant □Others

****To be filled in by subject**** _______________________

PART 1： Demographics

This part's questions are about your family. Please select the answers that best describes your family.

b1a. Number of Biological Siblings (including living and deceased siblings but not including yourself):
b1b. ___Elder brother b1c. ___Elder sister b1d. ___Younger brother b1e. ___Younger sister b1f. N/A

b2a. Marital Status: □Single (Never been married) □Married □Divorced/Seperated □Widowed

b3a. Numbers of Biological Off-springs (including living and deceased off-springs):
b3b. ___.Son b3c. ___Daughter b3d. N/A

b4a. Your dialect / mother tongue is (Multiple responses allowed)?
□Tanka dialect □Chaozhou dialect □Hakka dialect □Minnan Dialect
□Cantonese (Hong Kong) □Cantonese (Guangzhou) □Taishanese (Siyi)
□Cantonese (Zhongshan) □Cantonese (Dongguan) □Wu-Hua (Wuzhou)
□Others Cantonese dialect □Hunan dialect □Mandarin □Guangxi dialect □Others

b4b. Your mother's dialect / mother tongue is (Multiple responses allowed)?
□Tanka dialect □Chaozhou dialect □Hakka dialect □Minnan Dialect
□Cantonese (Hong Kong) □Cantonese (Guangzhou) □Taishanese (Siyi)
□Cantonese (Zhongshan) □Cantonese (Dongguan) □Wu-Hua (Wuzhou)
□Others Cantonese dialect □Hunan dialect □Mandarin □Guangxi dialect □Others

b5a. Your birthplace was?
□Hong Kong (Jump to b5b) □Macau (Jump to b5b) □Guangxi Province (Jump to b5b)
□Guangdong Province (Jump to b6a) □Fujian Province(Jump to b7a)
□Hunan Province (Jump to b5b) □Other Chinese provinces (Jump to b5b)
□Other countries (Jump to b8a) □Do not know (Jump to b5b)

b5b. Your biological mother's birthplace was?
□Hong Kong (Jump to b12a) □Macau (Jump to b12a) □Guangxi Province (Jump to b12a)
□Guangdong Province (Jump to b9a) □Fujian Province (Jump to b10a)
□Hunan Province (Jump to b12a) □Other Chinese provinces (Jump to b12a)
□Other countries (Jump to b11a) □Do not know (Jump to b12a)

b6a. Your birthplace was in which part of Guangdong Province?
□Guangzhou □Shenzhen (Baoan) □Zhongshan □Zhuhai □Foshan
□Maoming □Zhaoqing □Huizhou □Zhanjiang □Jiangmen □Heyuan
□Shaoguan □Dongguan □Shanwei □Yangjiang □Meizhou □Qingyuan
□Yunfu □Chaozhou □Shantou □Jieyang □Do not know (Jump to b9a/b10a/b11a)

b7a. Your birthplace was in which part of Fujian Province?
□Fuzhou □Putian □Sanming □Nanping □Longyan □Ningde
□Zhangzhou □Quanzhou □Xiamen □Do not know (Jump to b9a/b10a/b11a/b12a)

b8a. Your birthplace was in which country? □Malaysia □Thailand □Other countries

b9a. Mother's birthplace was in which part of Guangdong Province?

□Guangzhou □Shenzhen (Baoan) □Zhongshan □Zhuhai □Foshan
□Maoming □Zhaoqing □Huizhou □Zhanjiang □Jiangmen □Heyuan
□Shaoguan □Dongguan □Shanwei □Yangjiang □Meizhou □Qingyuan
□Yunfu □Chaozhou □Shantou □Jieyang □Do not know (Jump to b12a)

b10a. Mother's birthplace was in which part of Fujian Province?
□Fuzhou □Putian □Sanming □Nanping □Longyan □Ningde
□Zhangzhou □Quanzhou □Xiamen □Do not know (Jump to b12a)

b11a. Mother's birthplace was in which country? □Malaysia □Thailand □Other countries

(Please answer either one question)
b12a. How long have you lived in your birthplace? Until ______ years old
b12b. Or until which year _______

b13a. Which housing type best describes the accommodation you lived in when you were 10 years old ?
□Temporary housing/cage-home □Public housing □Home Ownership Scheme housing
□Private housing (owner) □Private housing (rent) □Boat □Shanty house (wood)
□Stone house □Village house □Others □Do not know

b14a. Your highest education level is?
□No formal education □Old-style private school □Primary school
□Middle school (Form 1 to 3) □High school (Form 4 to 5) □Matriculation (Form 6 to 7)
□Technical/Vocational institute □University degree or above □Do not know

b14b. Your biological father's highest education level is?
□No formal education □Old-style private school □Primary school
□Middle school (Form 1 to 3) □High school (Form 4 to 5) □Matriculation (Form 6 to 7)
□Technical/Vocational institute □University degree or above □Do not know

b14c. Your biological mother's highest education level is?
□No formal education □Old-style private school □Primary school
□Middle school (Form 1 to 3) □High school (Form 4 to 5) □Matriculation (Form 6 to 7)
□Technical/Vocational institute □University degree or above □Do not know

PART 2： Health Record

Now we want to know about your past medical history.

c1b. (Excluding current NPC diagnosis) Have you ever been diagnosed with any cancers? □No □Yes

c1c. If yes, please select (Multiple responses allowed):

□Lung cancer □Liver cancer □Gastric cancer □Colorectal cancer □Esophageal cancer
□Blood cancer (leukemia) □Pancreatic cancer □Brain cancer □Bladder cancer
□Non-Hodgkin lymphomas □Breast cancer (female) □Cervical cancer (female)
□Cancer of the uterus (female) □Ovarian cancer (female) □Prostate cancer (male)
□Others: ____________________________

(Medical history 1) If ever diagnosed with any above cancer, please answer the following questions (If ever diagnosed with more than one cancer, please answer the following questions correspondingly):

a. When were you diagnosed with this cancer? (Please answer either one option)
b. Age: _______ years old;
c. □Unclear
d. At which age group were you diagnosed with cancer?
□0-4 years old □5-9 years old □10-14 years old □15-19 years old □20-24 years old
□25-29 years old □30-34 years old □35-39 years old □40-44 years old □45-49yearsold
□50-54 years old □55-59 years old □60-64 years old □65-69 years old □70-74 years old
□75-79 years old □80-84 years old □85 years old or above □Unclear
e. Have you ever received chemotherapy? □No □Yes
f. Have you ever received radiotherapy? □No □Yes

(Medical history 2) If ever diagnosed with any above cancer, please answer the following questions (If ever diagnosed with more than one cancer, please answer the following questions correspondingly):

a. When were you diagnosed with this cancer? (Please answer either one option)
b. Age: _______ years old;
c. □Unclear
d. At which age group were you diagnosed with cancer?
□0-4 years old □5-9 years old □10-14 years old □15-19 years old □20-24 years old
□25-29 years old □30-34 years old □35-39 years old □40-44 years old □45-49yearsold
□50-54 years old □55-59 years old □60-64 years old □65-69 years old □70-74 years old
□75-79 years old □80-84 years old □85 years old or above □Unclear
e. Have you ever received chemotherapy? □No □Yes
f. Have you ever received radiotherapy? □No □Yes

(Medical history 3) If ever diagnosed with any above cancer, please answer the following questions (If ever diagnosed with more than one cancer, please answer the following questions correspondingly):

a. When were you diagnosed with this cancer? (Please answer either one option)
b. Age: _______ years old;
c. □Unclear
d. Or at which age group were you diagnosed with cancer?
□0-4 years old □5-9 years old □10-14 years old □15-19 years old □20-24 years old
□25-29 years old □30-34 years old □35-39 years old □40-44 years old □45-49yearsold
□50-54 years old □55-59 years old □60-64 years old □65-69 years old □70-74 years old
□75-79 years old □80-84 years old □85 years old or above □Unclear

e. Have you ever received chemotherapy? □No □Yes
f. Have you ever received radiotherapy? □No □Yes

c19a. Have you ever undergone any surgery? □No □Yes
c19b. If yes, please state (Please answer either one option):
c19c. Surgery age: ____ years old
c19d. Or which year? _________
c19e. Reason for surgery: □Cancer □Injury □Others

c20a. Prior to NPC diagnosis, have you ever told by a doctor that you had the following non-infectious diseases?
□No □Yes
c20b. If yes, please specify (Multiple choices allowed):
□Hypertesnsion □Diabetes mellitus □Stroke □Coronary heart disease
□Chronic Sinusitis □Nasal Polyps □Pneumoconiosis (Silicosis/Asbestosis)
□Peptic ulcer disease □Chronic nasal congestion □Allergic Rhinitis
□Gastroesophageal reflux □Chronic obstructive pulmonary disease □Osteoporosis
□Cystic fibrosis □Crohn's disease □Fatty liver disease
c20c. Prior to NPC diagnosis, have you ever told by a doctor that you had the following infectious diseases?
□No □Yes
c20d. If yes, please specify (Multiple choices allowed):
□Infectious mononucleosis (kissing disease) □Pneumonia □Otitis media □Hepatitis (Jump to c21a)
□Sexually Transmitted disease □Genital warts (e.g. condyloma acuminatum) □AIDS or HIV infection
c21a. Which type of hepatitis? □Hepatitis A □Hepatitis B □Hepatitis C □Do not know

d1. Infancy
d1a. Below are some questions about your mother's pregnancy with you and breastfeeding habits.

d1b. While your mother was pregnant with you, did your mother experience any health difficulty?
□No (Jump to d1d) □Yes □Do not know (Jump to d1d)

d1c. If yes, please specify: □Anemia □Hypertension □Gestational diabetes □Others

d1d. While your mother was pregnant with you, did your mother experience any childbirth complications?
□No (Jump to d2a) □Yes □Do not know (Jump to d2a)

d1e. If yes, please specify:
□Malposition □Premature rupture of membranes □Umbilical cord entanglement □Others

d2a. Birth delivery mode:
□Natural birth (Jump to d2c) □Caesarean section □Unknown (Jump to d2c)

d2b. Was it an emergency caesarean section?
□Planned caesarean section □Emergency caesarean section □Do not know

d2c. Was it a single birth or multiple births? □Single birth □Multiple births □Do now know

d2e. Your birth was:
□Premature birth_____weeks (d2f)

□Full term birth
□Postmature birth
□Do not know (Jump to d4a)

d4a. Your birth weight: ______ kg; d4b. □Unclear

d5a. Were you breastfed as an infant?
□Always breastfed, without formula fed
□Initially breastfed, then formula fed
□Sometimes breastfed, sometimes formula fed
□No breastfed, always formula fed
□Do not know (Jump to e1a)
d5b. How many months were you breastfed? ___months d5c. □Do not know
e1a. Family Cancer History

We would like to know more about your family cancer history. Please note that the family members here refer to your direct blood relatives, including living and deceased members, but not including adopted offsprings, legal parents and siblings. Please try your best to recall and provide as much information as you can. For example, you do not remember the age of the family member when he/she was diagnosed with cancer, but you remember it is around 40 years old. Then please answer around 40 years old. If you do not remember the details, please select "Unclear".

e1b. Has any of your family members ever been diagnosed with cancer, excluding recurrence and metastasis?
□No □Yes

e1c. If yes, please specify (Multiple responses allowed):
□Mother □Father □Brother □Sister □Paternal grandfather □Paternal grandmother
□Maternal grandfather □Maternal grandmother □Daughter □Son □Others (Jump to e158aa)

If any of your family members has been ever diagnosed with any above cancer, please answer the following questions. If the family member has been ever diagnosed with more than one cancer, please specify the conditions of each cancer.

(Family member 1)
a. Which cancer has your family member been diagnosed with (excluding recurrence and metastasis)?
(Multiple responses allowed)
□Nasopharyngeal carcinoma cancer □Lung cancer □Liver cancer □Gastric cancer
□Colorectal cancer □Esophageal cancer □Blood cancer (leukemia) □Pancreatic cancer
□Brain cancer □Bladder cancer □Non-Hodgkin lymphomas □Breast cancer (female)
□Cervical cancer (female) □Cancer of the uterus (female) □Ovarian cancer (female)
□Prostate cancer (male) □Others: ______________________________

a. When was the family member diagnosed with this cancer? (Please answer either one option)
b. Age: _______ years old;
c. □Unclear
d. Or at which age group were you diagnosed with cancer?
□0-4 years old □5-9 years old □10-14 years old □15-19 years old □20-24 years old
□25-29 years old □30-34 years old □35-39 years old □40-44 years old □45-49yearsold
□50-54 years old □55-59 years old □60-64 years old □65-69 years old □70-74 years old

□75-79 years old □80-84 years old □85 years old or above □Unclear

(Family member 2)

a. Which cancer has your family member been diagnosed with (excluding recurrence and metastasis)? (Multiple responses allowed)

□Nasopharyngeal carcinoma cancer □Lung cancer □Liver cancer □Gastric cancer
□Colorectal cancer □Esophageal cancer □Blood cancer (leukemia) □Pancreatic cancer
□Brain cancer □Bladder cancer □Non-Hodgkin lymphomas □Breast cancer (female)
□Cervical cancer (female) □Cancer of the uterus (female) □Ovarian cancer (female)
□Prostate cancer (male) □Others: ______________________________

a. When was the family member diagnosed with this cancer? (Please answer either one option)
b. Age: _______ years old;
c. □Unclear
d. Or at which age group were you diagnosed with cancer?
□0-4 years old □5-9 years old □10-14 years old □15-19 years old □20-24 years old
□25-29 years old □30-34 years old □35-39 years old □40-44 years old □45-49yearsold
□50-54 years old □55-59 years old □60-64 years old □65-69 years old □70-74 years old
□75-79 years old □80-84 years old □85 years old or above □Unclear

e158aa. How many of your other family members were diagnosed with cancer?
□1 □2 □3 □4 □5

e158a. Your relationship with other family member (1):
□Father's older brother □Father's younger brother □Father's older sister
□Father's younger sister □Father's brother's offspring □Mother's brother □Mother's older sister
□Mother's younger sister □Father's sister's offspring or mother's sibling's offspring

e158b. Your relationship with other family member (2):
□Father's older brother □Father's younger brother □Father's older sister
□Father's younger sister □Father's brother's offspring □Mother's brother □Mother's older sister
□Mother's younger sister □Father's sister's offspring or mother's sibling's offspring

e158c. Your relationship with other family member (3):
□Father's older brother □Father's younger brother □Father's older sister
□Father's younger sister □Father's brother's offspring □Mother's brother □Mother's older sister
□Mother's younger sister □Father's sister's offspring or mother's sibling's offspring

e158d. Your relationship with other family member (4):
□Father's older brother □Father's younger brother □Father's older sister
□Father's younger sister □Father's brother's offspring □Mother's brother □Mother's older sister
□Mother's younger sister □Father's sister's offspring or mother's sibling's offspring

e158e. Your relationship with other family member (5):
□Father's older brother □Father's younger brother □Father's older sister
□Father's younger sister □Father's brother's offspring □Mother's brother □Mother's older sister
□Mother's younger sister □Father's sister's offspring or mother's sibling's offspring

(Other family member 1)

aa. How many times was other family member diagnosed with cancer? □Once □Twice □Three times

a. Which cancer has your other family member been diagnosed with for the first time (excluding recurrence and metastasis) (Multiple responses allowed) ?

□Nasopharyngeal carcinoma cancer □Lung cancer □Liver cancer □Gastric cancer
□Colorectal cancer □Esophageal cancer □Blood cancer (leukemia) □Pancreatic cancer
□Brain cancer □Bladder cancer □Non-Hodgkin lymphomas □Breast cancer (female)
□Cervical cancer (female) □Cancer of the uterus (female) □Ovarian cancer (female)
□Prostate cancer (male) □Others: (b)_______________________________

c. When was the family member diagnosed with this cancer? Age: _______ years old;

d. □Unclear

e. Or at which age group were you diagnosed with cancer?

□0-4 years old □5-9 years old □10-14 years old □15-19 years old □20-24 years old
□25-29 years old □30-34 years old □35-39 years old □40-44 years old □45-49yearsold
□50-54 years old □55-59 years old □60-64 years old □65-69 years old □70-74 years old
□75-79 years old □80-84 years old □85 years old or above □Unclear

f. Which cancer has your other family member been diagnosed with for the second time (excluding recurrence and metastasis) (Multiple responses allowed) ?

□Nasopharyngeal carcinoma cancer □Lung cancer □Liver cancer □Gastric cancer
□Colorectal cancer □Esophageal cancer □Blood cancer (leukemia) □Pancreatic cancer
□Brain cancer □Bladder cancer □Non-Hodgkin lymphomas □Breast cancer (female)
□Cervical cancer (female) □Cancer of the uterus (female) □Ovarian cancer (female)
□Prostate cancer (male) □Others: (g)_______________________________

h. When was the family member diagnosed with this cancer? Age: _______ years old;

i. □Unclear

j. Or at which age group were you diagnosed with cancer?

□0-4 years old □5-9 years old □10-14 years old □15-19 years old □20-24 years old
□25-29 years old □30-34 years old □35-39 years old □40-44 years old □45-49yearsold
□50-54 years old □55-59 years old □60-64 years old □65-69 years old □70-74 years old
□75-79 years old □80-84 years old □85 years old or above □Unclear

k. Which cancer has your other family member been diagnosed with for the third time (excluding recurrence and metastasis) (Multiple responses allowed) ?

□Nasopharyngeal carcinoma cancer □Lung cancer □Liver cancer □Gastric cancer
□Colorectal cancer □Esophageal cancer □Blood cancer (leukemia) □Pancreatic cancer
□Brain cancer □Bladder cancer □Non-Hodgkin lymphomas □Breast cancer (female)
□Cervical cancer (female) □Cancer of the uterus (female) □Ovarian cancer (female)
□Prostate cancer (male) □Others: (l)_______________________________

m. When was the family member diagnosed with this cancer? Age: _______ years old;

n. □Unclear

o. Or at which age group were you diagnosed with cancer?

□0-4 years old □5-9 years old □10-14 years old □15-19 years old □20-24 years old
□25-29 years old □30-34 years old □35-39 years old □40-44 years old □45-49yearsold
□50-54 years old □55-59 years old □60-64 years old □65-69 years old □70-74 years old
□75-79 years old □80-84 years old □85 years old or above □Unclear

(Other family member 2)

aa. How many times was other family member diagnosed with cancer? □Once □Twice □Three times

a. Which cancer has your other family member been diagnosed with for the first time (excluding recurrence and metastasis) (Multiple responses allowed) ?

□Nasopharyngeal carcinoma cancer □Lung cancer □Liver cancer □Gastric cancer
□Colorectal cancer □Esophageal cancer □Blood cancer (leukemia) □Pancreatic cancer
□Brain cancer □Bladder cancer □Non-Hodgkin lymphomas □Breast cancer (female)
□Cervical cancer (female) □Cancer of the uterus (female) □Ovarian cancer (female)
□Prostate cancer (male) □Others: (b)______________________________

c. When was the family member diagnosed with this cancer? Age: _______ years old;

d. □Unclear

e. Or at which age group were you diagnosed with cancer?

□0-4 years old □5-9 years old □10-14 years old □15-19 years old □20-24 years old
□25-29 years old □30-34 years old □35-39 years old □40-44 years old □45-49yearsold
□50-54 years old □55-59 years old □60-64 years old □65-69 years old □70-74 years old
□75-79 years old □80-84 years old □85 years old or above □Unclear

f. Which cancer has your other family member been diagnosed with for the second time (excluding recurrence and metastasis) (Multiple responses allowed) ?

□Nasopharyngeal carcinoma cancer □Lung cancer □Liver cancer □Gastric cancer
□Colorectal cancer □Esophageal cancer □Blood cancer (leukemia) □Pancreatic cancer
□Brain cancer □Bladder cancer □Non-Hodgkin lymphomas □Breast cancer (female)
□Cervical cancer (female) □Cancer of the uterus (female) □Ovarian cancer (female)
□Prostate cancer (male) □Others: (g)______________________________

h. When was the family member diagnosed with this cancer? Age: _______ years old;

i. □Unclear

j. Or at which age group were you diagnosed with cancer?

□0-4 years old □5-9 years old □10-14 years old □15-19 years old □20-24 years old
□25-29 years old □30-34 years old □35-39 years old □40-44 years old □45-49yearsold
□50-54 years old □55-59 years old □60-64 years old □65-69 years old □70-74 years old
□75-79 years old □80-84 years old □85 years old or above □Unclear

k. Which cancer has your other family member been diagnosed with for the third time (excluding recurrence and metastasis) (Multiple responses allowed) ?

□Nasopharyngeal carcinoma cancer □Lung cancer □Liver cancer □Gastric cancer
□Colorectal cancer □Esophageal cancer □Blood cancer (leukemia) □Pancreatic cancer
□Brain cancer □Bladder cancer □Non-Hodgkin lymphomas □Breast cancer (female)
□Cervical cancer (female) □Cancer of the uterus (female) □Ovarian cancer (female)
□Prostate cancer (male) □Others: (l)______________________________

m. When was the family member diagnosed with this cancer? Age: _______ years old;

n. □Unclear

o. Or at which age group were you diagnosed with cancer?

□0-4 years old □5-9 years old □10-14 years old □15-19 years old □20-24 years old
□25-29 years old □30-34 years old □35-39 years old □40-44 years old □45-49yearsold
□50-54 years old □55-59 years old □60-64 years old □65-69 years old □70-74 years old
□75-79 years old □80-84 years old □85 years old or above □Unclear

PART 3：Lifestyle

This part's questions are about your dietary hibts in the four stages of your life, including childhood (6 years old to 12 years old), adolescence (13 years old to 18years old), adulthood (19 years old to 30 years old) and 10 years ago.

fa1a.Lifecourse 1: childhood dietary habits during 6 - 12 years old

fa2c. Was your dietary habits influenced by the Cultural Revolution? □No □Yes

	Food	Lifecourse 1 (6 -12 years old)									
		Never consume	Less than once a month	Once to twice a month	1 to 3 times a week	4 to 6 times a week	Once to twice a day	More than 3 times a day	Average consumption portion		
									Small	Medium	Large
fa3h	Red and white meat (beef, pork, lamb, chicken, duck, goose, etc.) (Bowl)										
fa4l	Liver (pig, chicken, duck, goose liver, etc.) (Bowl)										
fa5h	Fish(Bowl)										
fa6h	Fruits (Bowl)										
fa7h	Vegetables (Bowl)										
fa7a	Fresh milk (Glass)										
fa8a	Milk powder (Glass)										
fa9h	Soy milk (Glass)										

fa10a. Which type of salted fish did you consume?

("Mouldy salted fish" refers to Cantonese salted fish, which is softer and salted freshly caught. "Firmed salted fish" is made from fish frozen before being salted and is almost translucent after drying. If you are unable to tell the type of salted fish, please select all types of salted fish.)

□No □Mouldy salted fish □Firm salted fish □Mouldy and firm salted fish

□Other salted fish □All types of salted fish □Do not know

	Food	Lifecourse 1 (6 -12 years old)									
		Never consume	Less than once a month	Once to twice a month	1 to 3 times a week	4 to 6 times a week	Once to twice a day	More than 3 times a day	Average consumption portion		
									Small	Medium	Large
fa11h	Mouldy salted fish (Spoon)										
fa12h	Firm salted fish (Spoon)										

fa13h	Other salted fish (Spoon)										
fa14h	All types of salted fish (Spoon)										
fa15h	Preserved meat (Bowl)										
fa16h	Preserved eggs										
fa17h	Preserved vegetables (Spoon)										
fa18h	Preserved fruits (Bowl)										

fb1a. Lifecourse 2: Adolescence dietary habits during 13 - 18 years old

	Food	Lifecourse 2 (13 -18 years old)						
		Never consume	Less than once a month	Once to twice a month	1 to 3 times a week	4 to 6 times a week	Once to twice a day	More than 3 times a day
fb1b	How often did you consume pan-fried food (pan-fried fish, pan-fried pork chop, deep-fried spring roll, deep fried chicken wings, etc.)?							
fb1c	How often did you consume barbeque meat (roast [illegible]							

fb1d. When you consume barbeque meat, did you consume the half-burnt and/or burnt part? □No □Yes

	Food	Lifecourse 2 (13 -18 years old)									
		Never consume	Less than once a month	Once to twice a month	1 to 3 times a week	4 to 6 times a week	Once to twice a day	More than 3 times a day	Average consumption portion		
									Small	Medium	Large
fb2h	Red meat (beef, pork, lamb, etc.) (Bowl)										
fb3h	White meat (chicken, duck, goose, etc.) (Bowl)										
fb4h	Liver (pig, chicken, duck, goose liver, etc.) (Bowl)										
fb5h	Oily fish (salmon, mackerel, tuna, sardines, trout, etc.) (Bowl)										

fb6h	Non oily fish (brown marbled grouper, green grouper, mangrove snapper, big eye, hairtail, etc.) (Bowl)										
fb7h	Shellfish (Bowl)										
fb8h	Green leafy vegetables (spinach, lettuce, Chinese kale or collard mustard, Chinese flowering cabbage, etc.) (Bowl)										
fb9h	Non-leafy vegetables (cucumber, zucchini, etc.) (Bowl)										
fb10h	Carrots (Bowl)										
fb11h	Tomatoes (Bowl)										
fb12h	Citrus fruits (orange, mandarin orange, pomelo, tangerine, etc.) (Bowl)										
fb13h	Other fruits (Bowl)										
fb14h	Fresh milk (Glass)										
fb15h	Milk powder (Glass)										
fb16h	Diary products (ice cream, yogurt, and cheese) (Glass)										
fb17h	Eggs (including egg yolks)										
fb18h	Tofu (Bowl)										
fb19h	Soy milk (Glass)										
fb20h	Bean curd (Bowl)										

fb21h. Which type of salted fish did you consume?
(“Mouldy salted fish” refers to Cantonese salted fish, which is softer and salted freshly caught. “Firmed salted fish” is made from fish frozen before being salted and is almost translucent after drying. If you are unable to tell the type of salted fish, please select all types of salted fish.)

□No □Mouldy salted fish □Firm salted fish □Mouldy and firm salted fish
□Other salted fish □All types of salted fish □Do not know

	Food	Lifecourse 2 (13 -18 years old)									
		Never consume	Less than once a month	Once to twice a month	1 to 3 times a week	4 to 6 times a week	Once to twice a day	More than 3 times a day	Average consumption portion		
									Small	Medium	Large
fb22h	Mouldy salted fish (Spoon)										

fb23h	Firm salted fish (Spoon)										
fb24h	Other salted fish (Spoon)										
fb25h	All types of salted fish (Spoon)										
fb26h	Dried seafood (Spoon)										
fb27h	Preserved vegetables (Spoon)										
fb28h	Preserved fruits (Bowl)										
fb29h	Preserved eggs										
fb30h	Preserved meat (Bowl)										
fb31h	Processed meat (Ham, sausage, luncheon meat, etc.) (Bowl)										
fb32h	Condiments (Spoon)										
fb33h	Green and white tea (Glass)										
fb34h	Oolong tea (Glass)										
fb35h	Red/black tea (Glass)										
fb36h	Milk tea (Glass)										
fb37h	Coffee (Glass)										
fb38h	Chinese herbal tea (Bowl)										

fc1aa. Lifecourse 3: Adulthood dietary habits during 19 - 30 years old

fc1a. Were there any changes to your dietary habits during 19-30 years old in comparison with those during 13-18 years old?

□No □Yes

fc2a. Were there any changes to your dietary habits of red and white meat during 19-30 years old in comparison with those during 13-18 years old?

□No □Yes

	Food	Lifecourse 3 (19 -30 years old)									
		Never consume	Less than once a month	Once to twice a month	1 to 3 times a week	4 to 6 times a week	Once to twice a day	More than 3 times a day	Average consumption portion		
									Small	Medium	Large
fc3h	Red meat (beef, pork, lamb, etc.) (Bowl)										
fc4h	White meat (chicken, duck, goose, etc.) (Bowl)										
fc5h	Liver (pig, chicken, duck, goose liver, etc.) (Bowl)										

fc6a. Were there any changes to your dietary habits of seafood and shellfish during 19-30 years old in comparison with those during 13-18 years old?

□No □Yes

	Food	Lifecourse 3 (19 -30 years old)									
		Never consume	Less than once a month	Once to twice a month	1 to 3 times a week	4 to 6 times a week	Once to twice a day	More than 3 times a day	Average consumption portion		
									Small	Medium	Large
fc7h	Oily fish (salmon, mackerel, tuna, sardines, trout, etc.) (Bowl)										
fc8h	Non oily fish (brown marbled grouper, green grouper, mangrove snapper, big eye, hairtail, etc.) (Bowl)										
fc9h	Shellfish (Bowl)										

fc10a. Were there any changes to your dietary habits of vegetables during 19-30 years old in comparison with those during 13-18 years old?

□No □Yes

	Food	Lifecourse 3 (19 -30 years old)									
		Never consume	Less than once a month	Once to twice a month	1 to 3 times a week	4 to 6 times a week	Once to twice a day	More than 3 times a day	Average consumption portion		
									Small	Medium	Large

fc11h	Green leafy vegetables (spinach, lettuce, Chinese kale or collard mustard, Chinese flowering cabbage, etc.) (Bowl)										
fc12h	Non-leafy vegetables (cucumber, zucchini, etc.) (Bowl)										
fc13h	Carrots (Bowl)										
fc14h	Tomatoes (Bowl)										

fc15a. Were there any changes to your dietary habits of fruits during 19-30 years old in comparison with those during 13-18 years old?

□No □Yes

	Food	Lifecourse 3 (19 -30 years old)									
		Never consume	Less than once a month	Once to twice a month	1 to 3 times a week	4 to 6 times a week	Once to twice a day	More than 3 times a day	Average consumption portion		
									Small	Medium	Large
fc16h	Citrus fruits (orange, mandarin orange, pomelo, tangerine, etc.) (Bowl)										
fc17h	Other fruits (Bowl)										

fc18a. Were there any changes to your dietary habits of diary products and egges during 19-30 years old in comparison with those during 13-18 years old?

□No □Yes

	Food	Lifecourse 3 (19 -30 years old)									
		Never consume	Less than once a month	Once to twice a month	1 to 3 times a week	4 to 6 times a week	Once to twice a day	More than 3 times a day	Average consumption portion		
									Small	Medium	Large
fc19h	Fresh milk (Glass)										
fc20h	Milk powder (Glass)										
fc21h	Diary products (ice cream, yogurt, and cheese) (Glass)										
fc22h	Eggs (including egg yolks)										

fc23a. Were there any changes to your dietary habits of soy products and eggs during 19-30 years old in comparison with those during 13-18 years old?

□No □Yes

	Food	Lifecourse 3 (19 -30 years old)									
		Never consume	Less than once a month	Once to twice a month	1 to 3 times a week	4 to 6 times a week	Once to twice a day	More than 3 times a day	Average consumption portion		
									Small	Medium	Large
fc24h	Tofu (Bowl)										
fc25h	Soy milk (Glass)										
fc26h	Bean curd (Bowl)										

fc27a. Were there any changes to your dietary habits of salted fish during 19-30 years old in comparison with those during 13-18 years old?

□No □Yes

	Food	Lifecourse 3 (19 -30 years old)									
		Never consume	Less than once a month	Once to twice a month	1 to 3 times a week	4 to 6 times a week	Once to twice a day	More than 3 times a day	Average consumption portion		
									Small	Medium	Large
fc28h	Mouldy salted fish (Spoon)										
fc29h	Firm salted fish (Spoon)										
fc30h	Other salted fish (Spoon)										
fc31h	All types of salted fish (Spoon)										

fc32a. Were there any changes to your dietary habits of preserved food during 19-30 years old in comparison with those during 13-18 years old?

□No □Yes

	Food	Lifecourse 3 (19 -30 years old)									
		Never consume	Less than once a month	Once to twice a month	1 to 3 times a week	4 to 6 times a week	Once to twice a day	More than 3 times a day	Average consumption portion		
									Small	Medium	Large
fc33h	Dried seafood (Spoon)										
fc34h	Preserved vegetables (Spoon)										
fc35h	Preserved fruits (Bowl)										
fc36h	Preserved eggs										
fc37h	Preserved meat (Bowl)										

fc38h	Processed meat (Ham, sausage, luncheon meat, etc.) (Bowl)										
fc39h	Condiments (Spoon)										

fc40a. Were there any changes to your dietary habits of beverages during 19-30 years old in comparison with those during 13-18 years old?

□No □Yes

	Food	Lifecourse 3 (19 -30 years old)									
		Never consume	Less than once a month	Once to twice a month	1 to 3 times a week	4 to 6 times a week	Once to twice a day	More than 3 times a day	Average consumption portion		
									Small	Medium	Large
fc41h	Green and white tea (Glass)										
fc42h	Oolong tea (Glass)										
fc43h	Red/black tea (Glass)										
fc44h	Milk tea (Glass)										
fc45h	Coffee (Glass)										
fc46h	Chinese herbal tea (Bowl)										

fd1aa. Lifecourse 4: Adulthood dietary habits 10 years ago

fd1a. Were there any changes to your dietary habits 10 years ago in comparison with those during 19-30 years old?

□No □Yes

fd2a. Were there any changes to your dietary habits of red and white meat 10 years ago in comparison with those during 19-30 years old?

□No □Yes

	Food	Lifecourse 4 (10 years ago)									
		Never consume	Less than once a month	Once to twice a month	1 to 3 times a week	4 to 6 times a week	Once to twice a day	More than 3 times a day	Average consumption portion		
									Small	Medium	Large

fd3h	Red meat (beef, pork, lamb, etc.) (Bowl)										
fd4h	White meat (chicken, duck, goose, etc.) (Bowl)										
fd5h	Liver (pig, chicken, duck, goose liver, etc.) (Bowl)										

fd6a. Were there any changes to your dietary habits of seafood and shellfish 10 years ago in comparison with those during 19-30 years old?

□No □Yes

	Food	Lifecourse 4 (10 years ago)									
		Never consume	Less than once a month	Once to twice a month	1 to 3 times a week	4 to 6 times a week	Once to twice a day	More than 3 times a day	Average consumption portion		
									Small	Medium	Large
fd7h	Oily fish (salmon, mackerel, tuna, sardines, trout, etc.) (Bowl)										
fd8h	Non oily fish (brown marbled grouper, green grouper, mangrove snapper, big eye, hairtail, etc.) (Bowl)										
fd9h	Shellfish (Bowl)										

fd10a. Were there any changes to your dietary habits of vegetables 10 years ago in comparison with those during 19-30 years old?

□No □Yes

	Food	Lifecourse 4 (10 years ago)									
		Never consume	Less than once a month	Once to twice a month	1 to 3 times a week	4 to 6 times a week	Once to twice a day	More than 3 times a day	Average consumption portion		
									Small	Medium	Large
fd11h	Green leafy vegetables (spinach, lettuce, Chinese kale or collard mustard, Chinese flowering cabbage, etc.) (Bowl)										
fd12h	Non-leafy vegetables (cucumber, zucchini, etc.) (Bowl)										

fd13h	Carrots (Bowl)										
fd14h	Tomatoes (Bowl)										

fd15a. Were there any changes to your dietary habits of fruits 10 years ago in comparison with those during 19-30 years old?

□No □Yes

	Food	Lifecourse 4 (10 years ago)									
		Never consume	Less than once a month	Once to twice a month	1 to 3 times a week	4 to 6 times a week	Once to twice a day	More than 3 times a day	Average consumption portion		
									Small	Medium	Large
fd16h	Citrus fruits (orange, mandarin orange, pomelo, tangerine, etc.) (Bowl)										
fd17h	Other fruits (Bowl)										

fd18a. Were there any changes to your dietary habits of diary products and egges during 19-30 years old in comparison with those during 13-18 years old?

□No □Yes

	Food	Lifecourse 4 (10 years ago)									
		Never consume	Less than once a month	Once to twice a month	1 to 3 times a week	4 to 6 times a week	Once to twice a day	More than 3 times a day	Average consumption portion		
									Small	Medium	Large
fd19h	Fresh milk (Glass)										
fd20h	Milk powder (Glass)										
fd21h	Diary products (ice cream, yogurt, and cheese) (Glass)										
fd22h	Eggs (including egg yolks)										

fd23a. Were there any changes to your dietary habits of soy products and eggs during 19-30 years old in comparison with those during 13-18 years old?

□No □Yes

	Food	Lifecourse 4 (10 years ago)									
		Never consume	Less than once a month	Once to twice a month	1 to 3 times a week	4 to 6 times a week	Once to twice a day	More than 3 times a day	Average consumption portion		
									Small	Medium	Large
fd24h	Tofu (Bowl)										
fd25h	Soy milk (Glass)										

fd26h	Bean curd (Bowl)										

fd27a. Were there any changes to your dietary habits of salted fish during 19-30 years old in comparison with those during 13-18 years old?

□No □Yes

	Food	Lifecourse 4 (10 years ago)									
		Never consume	Less than once a month	Once to twice a month	1 to 3 times a week	4 to 6 times a week	Once to twice a day	More than 3 times a day	Average consumption portion		
									Small	Medium	Large
fd28h	Mouldy salted fish (Spoon)										
fd29h	Firm salted fish (Spoon)										
fd30h	Other salted fish (Spoon)										
fd31h	All types of salted fish (Spoon)										

fd32a. Were there any changes to your dietary habits of preserved food during 19-30 years old in comparison with those during 13-18 years old?

□No □Yes

	Food	Lifecourse 4 (10 years ago)									
		Never consume	Less than once a month	Once to twice a month	1 to 3 times a week	4 to 6 times a week	Once to twice a day	More than 3 times a day	Average consumption portion		
									Small	Medium	Large
fd33h	Dried seafood (Spoon)										
fd34h	Preserved vegetables (Spoon)										
fd35h	Preserved fruits (Bowl)										
fd36h	Preserved eggs										
fd37h	Preserved meat (Bowl)										
fd38h	Processed meat (Ham, sausage, luncheon meat, etc.) (Bowl)										
fd39h	Condiments (Spoon)										

fd40a. Were there any changes to your dietary habits of beverages during 19-30 years old in comparison with those during 13-18 years old?

□No □Yes

	Food	Lifecourse 4 (10 years ago)									
		Never consume	Less than once a month	Once to twice a month	1 to 3 times a week	4 to 6 times a week	Once to twice a day	More than 3 times a day	Average consumption portion		
									Small	Medium	Large
fd41h	Green and white tea (Glass)										
fd42h	Oolong tea (Glass)										
fd43h	Red/black tea (Glass)										
fd44h	Milk tea (Glass)										
fd45h	Coffee (Glass)										
fd46h	Chinese herbal tea (Bowl)										

fe1a. Did you take any fish oil/fish liver oil/omega-3 deep-sea fish oil supplements?
□No(Jump to fe2a) □Yes
fe1b. When did you start taking the above supplements? From_____years old
fe1c. When did you stop taking the above supplements? Until_____years old
fe1d. How frequent did you take the above supplements?
□Less than once a month □Once to twice a month □1 to 3 times a week □4 to 6 times a week
□Once or twice a day □More than 3 times a day

fe2a. Do you take any vitamin supplements? □No(Jump to g1a) □Yes
fe2b. If yes, please specify (Multiple responses allowed):
□Multi-vitamin supplements □Vitamin A supplements □Vitamin B supplements
□Vitamin C supplements □Vitamin D supplements □Vitamin E supplements

fe3a. When did you start taking multi-vitamin supplements? From______years old
fe3b. When did you stop taking multi-vitamin supplements? Until______years old
fe3c. How frequent did you take your multi-vitamin supplements?
□Less than once a month □Once to twice a month □1 to 3 times a week □4 to 6 times a week
□Once or twice a day □More than 3 times a day

fe4a. When did you start taking Vitamin A supplements? From______years old
fe4b. When did you stop taking Vitamin A supplements? Until______years old
fe4c. How frequent did you take your Vitamin A supplements?
□Less than once a month □Once to twice a month □1 to 3 times a week □4 to 6 times a week
□Once or twice a day □More than 3 times a day

fe5a. When did you start taking Vitamin B supplements? From______years old
fe5b. When did you stop taking Vitamin B supplements? Until______years old
fe5c. How frequent did you take your Vitamin B supplements?

□Less than once a month □Once to twice a month □1 to 3 times a week □4 to 6 times a week
□Once or twice a day □More than 3 times a day

fe6a. When did you start taking Vitamin C supplements? From______years old
fe6b. When did you stop taking Vitamin C supplements? Until______years old
fe6c. How frequent did you take your Vitamin C supplements?
□Less than once a month □Once to twice a month □1 to 3 times a week □4 to 6 times a week
□Once or twice a day □More than 3 times a day

fe7a. When did you start taking Vitamin D supplements? From______years old
fe7b. When did you stop taking Vitamin D supplements? Until______years old
fe7c. How frequent did you take your Vitamin D supplements?
□Less than once a month □Once to twice a month □1 to 3 times a week □4 to 6 times a week
□Once or twice a day □More than 3 times a day

fe8a. When did you start taking Vitamin E supplements? From______years old
fe8b. When did you stop taking Vitamin E supplements? Until______years old
fe8c. How frequent did you take your Vitamin E supplements?
□Less than once a month □Once to twice a month □1 to 3 times a week □4 to 6 times a week
□Once or twice a day □More than 3 times a day

g1a. Body Figure

Next we would like to know the changes of your body figures during your lifecourse. Please select the drawing that best describes your body figure accordingly.

g1b. Which body figure drawing best represents your current size and shape?

g3a. Your current height is? ______ cm(1cm =0.39 in)
g3b. Your current weight is? ______ kg (1kg=2.2 lb)

g4a/g5a. Which body figure drawing best represents your size and shape 10 years ago?

g6a. Your weight 10 years ago is? ______ kg (1 kg=2.2lb)

g7a/g8a. Which body figure drawing best represents your size and shape during19-30 years old?

g9a/g10a. Which body figure drawing best represents your size and shape during 13-18 years old？

g11a/g12a. Which of the below figure drawings best represents your size and shape during 6-12 years old？

h1a. Oral Hygiene

h1b. Below are the questions on your oral hygiene.

h1c. How many dental caries do you have?

□No(Jump to h1e) □1-2 □3-4 □5 or above

h1d. Have many adult teeth were extracted due to dental caries?

□No(Jump to h1e) □1-2 □3-4 □5 or above

h1e. Have you ever had periodontal disease? □No(Jump to i1b) □Yes

h1f. Have many adult teeth were extracted due to periodontal disease?

□No(Jump to h1e) □1-2 □3-4 □5 or above

i1a. Active smoking

We would like to know about your active and passive smoking situation.

i1b. Have you ever smoked? (Smoked at least 1 cigarette for six months or longer)

□Never smoke(Jump to i4) □Current smoker □Ex-smoker

i1c. Currently or before you quit, how soon after you wake up do you smoke your first cigarette?

□Less than 5 minutes □6-30 minutes □31-60 minutes □More than 60 minutes

i1d. At what age did you start smoking? _____ years old

i2b. At what age did you stop smoking? _____ years old

i2c. How many years have you quit smoking? _____years (Please fill in “0” for less than a year)

i3b. During childhood and adolescence (before 18 years old), how many cigarettes did you usually smoke per day? ________sticks

i3c. During adulthood (after 18 years old), how many cigarettes did you usually smoke per day? _____sticks

i4. Passive smoking

Childhood and adolescence (6-18years old)
i4a. Before 18 years old, how many family members you live with smoked?
□None (Jump to i8b) □1 □2 □3

(Family member 1)

c. What was his/her relationship to you?
□Father □Mother □Paternal grandfather □Paternal grandmother
□Maternal grandfather □Maternal grandmother □Siblings □Others

d. How long were you expose to second-hand smoke every day?
□Less than 5 minutes □30-60minutes □1-2 hours □More than 2 hours

e. Before 18 years old, how many years did you live with the family member who smoke? ______years

(Family member 2)

c. What was his/her relationship to you?
□Father □Mother □Paternal grandfather □Paternal grandmother
□Maternal grandfather □Maternal grandmother □Siblings □Others

d. How long were you expose to second-hand smoke every day?
□Less than 5 minutes □30-60minutes □1-2 hours □More than 2 hours

e. Before 18 years old, how many years did you live with the family member who smoke? ______years

Adulthood (after 18 years old)

i8b. After 18 years old, how many family members you live with smoked?
□None (Jump to i12a) □1 □2 □3

(Family member 1)

c. What was his/her relationship to you?
□Father □Mother □Paternal grandfather □Paternal grandmother
□Maternal grandfather □Maternal grandmother □Siblings □Others

d. How long were you expose to second-hand smoke every day?
□Less than 5 minutes □30-60minutes □1-2 hours □More than 2 hours

e. Before 18 years old, how many years did you live with the family member who smoke? ______years

(Family member 2)

c. What was his/her relationship to you?
□Father □Mother □Paternal grandfather □Paternal grandmother
□Maternal grandfather □Maternal grandmother □Siblings □Others

d. How long were you expose to second-hand smoke every day?

□Less than 5 minutes □30-60minutes □1-2 hours □More than 2 hours

e. Before 18 years old, how many years did you live with the family member who smoke? ______years

i12a. We would like to know about your drinking situation.

i12b. Have you ever consumed alcoholic beverages, such as beer, wine or other spirits at least once a month for six months or long?

□ Never or only on special occasions (once or twice a year) (Jump to j1b)

□Current drinking □Ex-drinking

i12c. Do you currently consume any alcohol beverages at least once a month？ □No □Yes

i12d. How old were you when you started drinking alcohol at least once a month? ______years old

i13b. At what age did you stop drinking? ______years old

i13c. How long have you stopped drinking alcohol? ______years

i14b. Which type of alcoholic drinks do you consume? (Multiple responses allowed)

□Red wine □White wine □Beer □Hard liquor

	Alcoholic drinkis	Average consumption portion(Glass)							
		Never consume	Less than once a	Once a month	1 to 3 times a week	4 to 6 times a week	Once to twice a day	More than 3 times a day	Average consumption
i11h	Red wine (Glass)<br>(Medium Glass 100ml)								Glass
i16aa	White wine (Glass)<br>(Medium Glass 100ml)								Glass
i17b	Beer (Glass)<br>(Medium Glass 100ml)								Glass
i17c	Beer (Can)<br>(Medium Can 375ml)								Can
i17e	Beer (Bottle)<br>(Medium Bottle 330ml)								Bottle
i18h	Hard liquor(Shot)<br>(Small Shot 10ml)<br>(Medium Shot:30ml)								Shot

j5a. According to the skintones below, please select a complexion that best matches your skintone.

Which complexion best reprents your skin tone?

j5b. current face skintone: □1 □2 □3 □4

j5c. current hand skintone: □1 □2 □3 □4

j5d. hand skintone 10 years ago: □1 □2 □3 □4

j5e. hand skintone during 19-30 years old: □1 □2 □3 □4

j5f. hand skintone during 13-18 years old: □1 □2 □3 □4

j5g. hand skintone during 6-12 years old: □1 □2 □3 □4

PART 4：Occupational Exposures

k1b. Have you ever been exposed to dust, chemicals, fumes, acid or alkali in your work?
□No (Jump to k6b) □Yes □N/A (Jump to k6b)

k1c. If yes, please specify (Multiple responses allowed):
□Dust (Jump to k2c) □Chemicals (Jump to k3c)
□Fumes (Jump to k4c) □Acid or alkali (Jump to k5c)

Dust
k2c. Which dust were you exposed to (Multiple responses allowed)?
□Wood dust □Metal dust □Cotton dust □Leather dust □Asbestos dust
□Cement dust □Chalk dust □Coal dust □Ground dust □Incense burning □Others
k2d. Which job industry were you in when exposured to dust? _______________________
k2e. What was your job title? _______________________
k2f. How many years? _______years
k2g. To what extent have you been exposed to dust?
□Mild discomfort □Moderate discomfort □Severe discomfort

Chemical
k3c. Which chemicals were you exposed to (Multiple responses allowed)?
□Wood preservatives □Formaldehyde □Organic solvents □Pesticide
□Benzene □Dye □Paint □Others
k3d. Which job industry were you in when exposured to chemicals? _______________________
k3e. What was your job title? _______________________
k3f. How many years? _______years
k3g. To what extent have you been exposed to chemicals?
□Mild discomfort □Moderate discomfort □Severe discomfort

Fume
k4c. Which fumes were you exposed to (Multiple responses allowed)?
□Diesel □Gasoline □Coal □Firewood □Tar
□Natural gas □Exhaust gas □Welding fume □Others
k4d. Which job industry were you in when exposured to fumes? _______________________
k4e. What was your job title? _______________________
k4f. How many years? _______years
k4g. To what extent have you been exposed to fumes?
□Mild discomfort □Moderate discomfort □Severe discomfort

Acid/alkalik5c. Which acid or alkaliwere you exposed to (Multiple responses allowed)?
□Chromic acid □Sulphuric acid □Hydrochloric acid □Nitrate
□Concentrated alkaline solutions □Ammonia □Others
k5d. Which job industry were you in when exposured to acid or alkali? _______________________
k5e. What was your job title? _______________________
k5f. How many years? _______years
k5g. To what extent have you been exposed to acid or alkali?
□Mild discomfort □Moderate discomfort □Severe discomfort

pc10. Passive smoking at workplace

k6b. Do people smoke at your workplace before 18 years old?
□No (Jump to k7b) □Yes □N/A (Jump to k7b)
k6c. How long were you exposed to passive smoking at your workplace everyday?
□Less than 5 minutes □30-60 minutes □1-2 hours □More than 2 hours
k6d. How many years were you exposed to passive smoking at workplace? ______years

k7b. Do people smoke at your workplace after 18 years old?
□No (Jump to l1b) □Yes □N/A (Jump to l1b)
k7c. How long were you exposed to passive smoking at your workplace everyday?
□Less than 5 minutes □30-60 minutes □1-2 hours □More than 2 hours
k7d. How many years were you exposed to passive smoking at workplace? ______years

l1a. We would like to know about your residence.

b. Residence during ____years old
c. Type of fuel (Multiple responses allowed):
□Gas □Kerosene □Wood □Electricity □Coal □Biomass
□Liquefied Petroleum Gas □Others □Do not know
d. Type of cooking oil (Multiple responses allowed):
□Mixed oil □Animal fat □Rapeseed □Peanut oil □Soy bean oil
□Olive oil □Others □Do not know
e. How often does your household cook at home?
□Never □Monthly □1-3 times a week □4-6 times a week □Daily □2-3times a day
f. Primary source of drinking water:
□Pipeline systems (from central, municipal supply) □Wells □River or canal water
□Do not know
g. How often does your household have hosquito repellant?
□None □Very rarely □Monthly □Weekly □Daily
h. How often does your household have incense buring?
□None □Occasional (during festivals) □Every first day/fifteen in lunar canlendar □Daily

m1a. Income
m1b. Prior to your NPC diagnosis, what was your employment?
□Self-employed □Employed □Retired □Housewive □Student □Unemployed

m1c. Prior to your NPC diagnosis, what was your average monthly income (HK$)?
□No income □ Less than$15,000 □$15,000-24,999 □$25,000-39,999 □$40,000 or above

m1d. Prior to your NPC diagnosis, what was the average monthly household income (HK$)?
□No income □ Less than$15,000 □$15,000-24,999 □$25,000-39,999 □$40,000 or above

PART 5： Survey preference format

z1a. Have you ever used desktop computer, laptop, smartphone or tablet computer?
□No experience □Some experience (occasional user) □Extensive experience (daily user)

z1b. Which way do you prefer to answering the questionnaire?
□Paper and pencil □Tablet computer □No preference

z1c. Who do you prefer to answering the questionnaire?
□Yourself □Interviewer □Jointly by interviewer and yourself □No preference

z1d.What is your attitude towards using tablet computer to answer the questionnaire?
□Very unfavorable □ Unfavorable □Neutral □Favorable □Very favorable

z1e.What are your reasons for choosing tablet computer to answer the questionnaire (Multiple responses allowed)?
□Easy to present questionnaire □Clear Content □Easy to use to answer questions
□Short response time □Good data privacy □High Data security □Easy to hold the tablet computer

z1f. Did you encounter any difficulties when using the table computer?
____

z1g. Any comments or suggestions:
____

End of Questionnaire

- This is the end of the questionnaire. We are very grateful for your participation.
- Interviwer marks the receipt of supermarket coupon. Interviewer and the subject sign for the receivance of supermarket coupon.
- Interviwer distributes the supermarket coupon to the subject.

PART 6： Interviewer assessment

o1b/o1c. Which body figure drawing best represents subject's current size and shape?

According to the skintones below, please select a complexion that best matches the subjectintones.

o1d. subject's current face skintone: □1 □2 □3 □4
o1e. subject's current hand skintone: □1 □2 □3 □4

o2a. We would like to know who answer the questions of each part.

o2e. Section 1-4 (Demographics, health record, mother pregnancy and breastfeeding and family cancer history):
□Subject □Interviewer □Others
o2ea. Section 5 (Dietary habits during 6-12 years old):
□Subject □Interviewer □Others
o2eb. Section 5 (Dietary habits during 13-18 years old):
□Subject □Interviewer □Others
o2ec. Section 5 (Dietary habits during 19-30 years old):
□Subject □Interviewer □Others
o2ed. Section 5 (Dietary habits 10 years ago):
□Subject □Interviewer □Others
o2ee. Section 6-9 (Body figure, oral health, active and passive smoking and drinking):
□Subject □Interviewer □Others
o2ef. Section 10-13 (Exposure to sunlight, occupational exposure, residential exposure and income):
□Subject □Interviewer □Others
o2f. Main respondent to the questionnaire:
□Subject □Subject's parents □Subject's children □Subject's siblings
□Others □Interviewer

o2fa. Questionnaire was mainly administered by (Multiple responses allowed):
□Subject □Subject’s parents □Subject’s children □Subject’s siblings
□Others □Interviewer

o2g. Interviewer’s assessment of subject’s response reliability:
□Unreliable □Questionable □Reliable □High quality
o2ga. Unreliable Reasons: ____
o2gb. Others: ____

o2h. Any comments on the questionnaire:
____
____
____
___

o2i. Any comments on the interview:
____
____
____
___

Interviewer signature: ____________

* Images of body figure and skin tone are available from ZMM on request.
